# Engineering Zr–W Mixed Oxide Heterojunctions from UiO-66-Type MOFs Templates: Bridging DFT and Mechanistic Insights into Organic Abatement

**DOI:** 10.1021/acsomega.6c08746

**Published:** 2026-09-16

**Authors:** Cyrille Ghislain Fotsop, Alexandra Lieb, Franziska Scheffler

**Affiliations:** 9376 Otto-von-Guericke-University Magdeburg, Chemical Institute, Chair for Industrial Chemistry, Universitätsplatz 2, Magdeburg 39106, Germany

## Abstract

The release of harmful chemicals into the environment is still increasing. This study aims to develop highly efficient and stable hybrid oxide photocatalysts (t/m-ZrO_2_@WO_3_-X) through hydrothermally assisted pyrolysis routes, based on (Zr/W)­UiO-66-type MOFs template, suitable for the abatement of organic dyes such as methylene blue (MB) and indigo carmine (IC), using a mathematical approach. XRD, TGA, FTIR, XPS, EDX, SEM, photoluminescence, UV–vis DRS, N_2_ sorption, Raman analysis, electrochemical impedance spectroscopy (EIS), and cyclic voltammetry analyses demonstrated the formation of t-ZrO_2_, m-ZrO_2_ and monoclinic WO_3_ oxides. Under optimal conditions, the degradation yields of IC and MB were found to be 95.11% and 92.82%, respectively, as determined by mathematical modeling with an R^2^ value of 97.70%. The impact of different types of water (tap, river, and surface) and anions and cations on the degradation efficiency was evaluated. DFT and Monte Carlo studies revealed that the LUMO was distributed between the benzene and pyrrole rings, while the HOMO was oriented toward the pyrrole ring. Analysis of Fukui indices for radicals suggests that *N*-methylated sites are most susceptible to attack by •O_2_^–^, h^+^ and •OH radicals produced by photolysis. Analysis of radical scavengers suggests that h^+^, •O_2_^–^ and •OH species are responsible for dye breakdown. LC-MS analysis reveals dye mineralization through pyrrole ring cleavage, followed by carbonyl group oxidation and intermediate formation. The toxicity of IC and MB was assessed using the fathead minnow LC50 (96 h), oral rat LD50 (mg/kg), Daphnia magna LC50 (48 h), bioaccumulation factor, mutagenicity, and developmental toxicity. The presence of WO_3_ in the hybrid oxides plays a significant role in photon absorption and the efficient separation of photoinduced electrons. The materials proved to be stable after five cycles of use.

## Graphical abstract

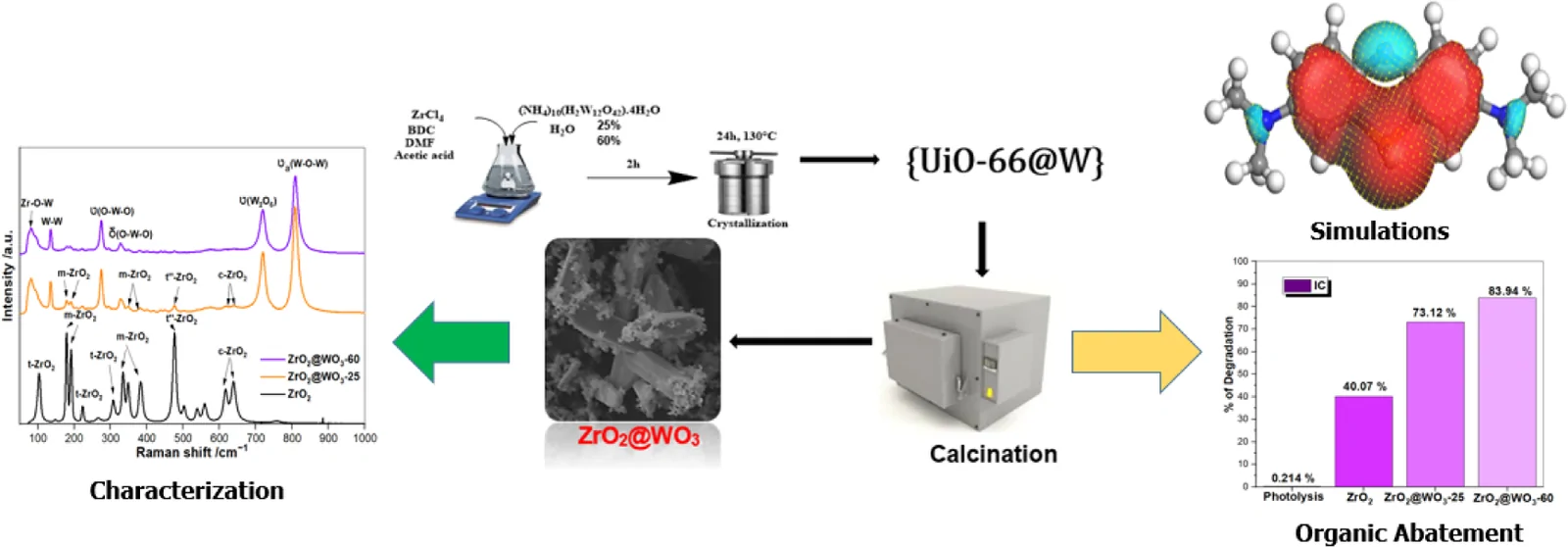

## 1 Introduction

The growth of the textile industry and the ongoing and increasing use of dyes undoubtedly have a negative impact on the environment.^1^ The most common and widely used synthetic dyes are methylene blue (MB), rhodamine B (RhB), methyl orange (MO) and indigo carmine (IC). These dyes are used in various industries, including textiles, food, cosmetics, printing, paper, pharmaceuticals, and paints, due to their low cost, remarkable color properties, and stability. Wastewater containing these dyes is likely to release these pollutants into the environment. Due to their resistance to biodegradation, the gradual accumulation of these dyes has harmful effects on both aquatic ecosystems and human health.^2^ It is therefore important to propose effective, sustainable solutions for continuously treating wastewater containing dyes. To this end, wastewater treatment involves methods such as membrane filtration, absorption and biological treatment. While these methods are effective, they have significant limitations, such as producing secondary sludge and incurring high maintenance costs.^3^ Furthermore, advanced oxidation processes such as photo-Fenton processes,^4,5^ and photocatalysis have attracted considerable interest due to their ease of implementation and ability to generate superoxide radicals (•O_2_^–^), holes (h^+^) and hydroxyl radicals (•OH), enabling the complete mineralization of pollutants in solution.^6–8^ Photocatalysis relies on the excitation of a semiconductor when irradiated with energy-bearing radiation, enabling electrons to move from the valence to conduction band.^4,9,10^ This process allows an electron to move from the highest occupied molecular orbital (HOMO) to the lowest unoccupied molecular orbital (LUMO), generating electrons (e^–^) at the LUMO level and holes (h^+^) at the HOMO level,.^11,12^ These electrons and holes are necessary for redox reactions on the photocatalyst surface, leading to pollutant degradation. Several semiconductors have been studied as photocatalysts, including TiO_2_,^13^ Fe_2_O_3_,^14^ Cu–Fe_2_O_3_,^4^ CuO/Fe_2_O_3_,^15^ ZnFe_2_O_4_/ZnO,^16^ SrTiO_3_/CeO_2_,^17^ Bi_2_O_3_/BiOCl,^18^ ZrO_2_ ^1^ and Cu@Cu_2_O@CuO.^19^ The efficiency of a semiconductor depends closely on its properties, such as bandgap width, crystal phase, morphology, specific surface area and charge recombination efficiency.

Among these metal oxides, zirconium dioxide (ZrO_2_) has attracted the most attention, as both zirconium (Zr) and titanium (Ti) belong to Group 4 of the periodic table. Unlike titanium dioxide (TiO_2_), ZrO_2_ is insoluble in water, making it the preferred choice as a photocatalyst for this study. ZrO_2_ is classified as a semiconductor due to its high thermal and chemical stability, suitable wide bandgap (ranging from 3.1 to 5.2 eV) and high visible light absorption efficiency. Its ability to store electrons and generate oxygen vacancies and holes after light absorption,^1^ combined with its reducing power and variable oxidation states, as well as its different crystal forms: monoclinic (m-), tetragonal­(t-) and cubic­(c-), makes it suitable for catalytic applications.^20^ Several authors have highlighted the influence of crystalline phases and structural defects on the photocatalytic performance of ZrO_2_. The presence of oxygen vacancies, combined with the coexistence of t-, m- and c-ZrO_2_ phases, promotes the formation of electron and hole traps while enhancing the separation of photogenerated charges.^21^ Thus, Shu et al. (2013)^22^ employed the hydrothermal method to synthesize star-shaped, tetrahedral ZrO_2_ nanostructures for the photodegradation of methyl orange (MO). The authors observed the complete degradation of MO within 1 h at various pH levels. However, these nanomaterials’ stability and reusability were not evaluated, raising questions about their long-term applicability. Basahel et al. (2015)^1^ investigated the impact of mesoporous ZrO_2_ nanocrystal phases on the photocatalytic degradation of the MO dye. They found that the m-ZrO_2_ phase showed the best photocatalytic performance, which was comparable to that of the c-ZrO_2_ and t-ZrO_2_ phases. This was due to the presence of oxygen vacancies in the ZrO_2_, which were associated with the coexistence of monoclinic phases.^1^

However, the photocatalytic performance of ZrO_2_ can be improved by combining it with other semiconductors to reduce its wide bandgap. Combining nanostructures, such as carbon nanotubes, with hierarchical ZrO_2_ architectures has proven effective for the photocatalytic degradation of dyes.^1,23^ Carbon-based materials, such as graphene (GR), are often used as support materials. For instance, ZrO_2_/GR has been employed for the degradation of MO dye, as demonstrated by Rani et al. (2014)^24^ and for the degradation of RB and MB dyes in wastewater, as shown by Rani et al. (2016).^3^ Furthermore, tungsten trioxide (WO_3_) is classified as an n-type semiconductor. It offers several advantages over other metal oxides, such as ZnO, SnO_2_, CdO and TiO_2_, including high thermal and chemical stability, efficient absorption of visible radiation at around 450 nm,^25^ high visible light absorption capacity and a suitable bandgap ranging from 2.5 to 2.8 eV.^26^ These properties, combined with its strong oxidizing power and variable oxidation states, enable it to generate holes (oxygen vacancies),^27^ enhance the recombination of photogenerated electron-hole pairs and improve electron transfer kinetics.^25,28^ Consequently, combining WO_3_ with other metal oxide semiconductors has proven interesting for catalytic applications. For instance, Li et al. (2019)^29^ synthesized WO_3_/Bi_2_MoO_6_ using an electrospinning-calcination-solvothermal approach to degrade RhB dye and tetracycline. They observed that, due to the simultaneous presence of WO_3_ and Bi_2_MoO_6_, photogenerated h^+^ and •O_2_^–^ were the dominant species during the degradation process, comparable to pure WO_3_.^29^ In another study, Abbas et al. (2020) developed Fe_2_Zr_2_-x­(WxO_7_)-type nanofluorites for the degradation of MB dye under visible light. They observed that the cubic crystalline phase of undoped iron fluorite (Fe_2_Zr_2_O_7_) changed upon the addition of m-ZrO_2_ and rhombohedral-Fe_2_O_3_ phases.^26^ Although the monoclinic phase of WO_3_ is the most thermodynamically stable, its crystal structure varies depending on the synthesis conditions and temperature. Various methods have proven effective for the synthesis of WO_3_ nanoparticles, including coprecipitation,^25^ ion-exchange-assisted pyrolysis,^30^ the sol-gel process and chemical precipitation.^25^ However, hydrothermally assisted pyrolysis routes remain largely unexplored. This study presents a novel approach that combines the hydrothermal route with pyrolysis to develop highly efficient, stable heterojunction photocatalysts derived from (Zr/W) UiO-66 for degrading IC and MB dyes under visible light irradiation using an advanced mathematical approach commonly known as response surface methodology (RSM). The center-composite design (CCD) is one of the most widely used RSM designs due to its accuracy, reproducibility, and ability to handle complex processes.^31^ The CCD has the advantage of simultaneously considering all the factors involved and providing information for an in-depth analysis of each experiment. The choice of ZrO_2_@WO_3_ was driven by the complementary properties of these two semiconductors. ZrO_2_ offers high thermal and chemical stability, is insoluble in water, and can generate oxygen vacancies that act as charge-trapping sites and promote charge separation. However, its relatively wide bandgap limits its utilization of visible light. WO_3_ was therefore coupled with ZrO_2_ due to its narrower bandgap (2.5–2.8 eV), strong visible-light absorption, high chemical stability and oxidation ability. The ZrO_2_@WO_3_ heterostructure is therefore expected to combine ZrO_2_’s structural stability and defect-mediated charge separation with WO_3_‘s visible-light response and redox properties, thereby reducing charge recombination and improving photocatalytic efficiency. Compared with many emerging photocatalysts, this oxide-based system offers the additional advantages of chemical stability, water resistance, relatively simple synthesis and potential reusability. Despite the existence of several ZrO_2_- and WO_3_-based photocatalysts, challenges remain regarding charge separation and a clear understanding of the photocatalytic mechanism.

This study aims to develop ZrO_2_@WO_3_-X photocatalytic materials using hydrothermally assisted pyrolysis route based on (Zr/W) UiO-66 MOF template materials. The aim is to convert the precursor into hybrid oxides with different crystalline phases (tetragonal, monoclinic, and hexagonal) as well as high specific surface areas. The originality of this work lies in the simple and reproducible experimental approach, the evaluation of the morphological, structural, and photocatalytic properties of the materials for the abatement of IC and MB in aqueous medium, the use of the CCD mathematical approach to evaluate these properties, the identification of vulnerable sites susceptible to radical attack using DFT and Monte Carlo (MC) methods. Furthermore, the photodegradation mechanism is studied through electron-hole quenching and compares the materials’ activity in real water samples with a view to potential applications in the sustainable treatment of industrial wastewater. The synthesized hybrid materials were characterized using X-ray diffraction (XRD), X-ray energy dispersive analysis (EDX), thermogravimetric analysis (TGA), infrared spectroscopy (FTIR), X-ray photoelectron spectroscopy (XPS), transmission electron microscopy (TEM), ultraviolet–visible (UV–vis) spectroscopy (DRS), photoluminescence (PL) analysis, nitrogen (N_2_) sorption analysis, cyclic voltammetry, Raman and electrochemical impedance (EIS) spectroscopy.

## 2 Experimentation

### 2.1 Reagents

The reagents used in this study are terephthalic acid (BDC), N,N-dimethylformamide (DMF, 99%), zirconium tetrachloride (ZrCl4, 99%), acetic acid (AC, 99%), ammonium metatungstate hydrate ((NH_4_)_6_H_2_W_12_O_40_·H_2_O, 99.5%), methanol (97%), ethanol (98%), 1,4-benzoquinone (p-BQ, 98%), disodium ethylenediaminetetraacetic acid (EDTA, 2Na, 99%), tert-butanol (*t*-BuOH, 99%), indigo carmine (IC), hydrogen peroxide (HP, 35 wt %), sodium carbonate (Na_2_CO_3_, 99%), sodium nitrate (NaNO_3_, 99%), sodium chloride (NaCl, 99%), sodium sulfate (Na_2_SO_4_, 97%), calcium chloride dihydrate (CaCl_2_·2H_2_O, 99%), potassium chloride (KCl, 98.5%), magnesium chloride hexahydrate (MgCl_2_·2H_2_O, 98%), and methylene blue (MB). All were of technical grade (Sigma-Aldrich, St. Louis, USA) and distilled water was used without further purification during the synthesis of (Zr)-UiO-66 and (Zr/W)-UiO-66.

### 2.2 Synthesis of (Zr)-UiO-66 and (Zr/W)-UiO-66

These materials were synthesized via solvothermal synthesis. For (Zr)-UiO-66, 7 mmol of BDC and 7 mmol of ZrCl_4_ were separately dissolved in 40 and 45 mL of DMF, respectively, for 50 min. These solutions were then combined and 5 mL of acetic acid was added, after which the mixture was stirred continuously at room temperature for 50 min. The resulting solution was transferred to a Teflon-lined autoclave, heated to 130 °C for 24 h, and then the white product was centrifuged and washed with DMF and methanol. Finally, the product was dispersed in methanol for 24 h to exchange the intra-framework DMF. This mixture was then centrifuged, rinsed with methanol and dried at 80 °C for 19 h. The final material obtained was designated (Zr)-UiO-66. For the synthesis of (Zr/W)-UiO-66, tungsten was incorporated in situ via a one-step solvothermal method during the reaction. The protocol was the same, but two different tungsten concentrations were used: 25% and 60%. (NH_4_)_6_H_2_W_12_O_40_·H_2_O was dissolved in 8 ml of water and added to the BDC and ZrCl_4_ solution, followed by the addition of 5 mL of acetic acid. After heating at 130 °C for 24 h, centrifugation and drying, the resulting products were designated (Zr/W)-UiO-66(25%) and (Zr/W)-UiO-66(60%).

#### 2.2.1 Synthesis of ZrO_2_, ZrO_2_@WO_3_-25 and ZrO_2_@WO_3_-60

The ZrO_2_, ZrO_2_@WO_3_-25 and ZrO_2_@WO_3_-60 samples were obtained through the thermal decomposition of Zr-UiO-66 and Zr/W-UiO-66 samples within a muffle furnace. 2 g of each sample were placed in aluminum crucibles and heated at 5 K/min within a temperature range of 25–1100 °C. The resulting samples were labeled ZrO_2_, ZrO_2_@WO_3_-25 and ZrO_2_@WO_3_-60 for the Zr-UiO-66%, Zr/W-UiO-66–25% and Zr/W-UiO-66–60%, respectively. The experimental procedure is shown in Scheme 1.

**Scheme 1:**
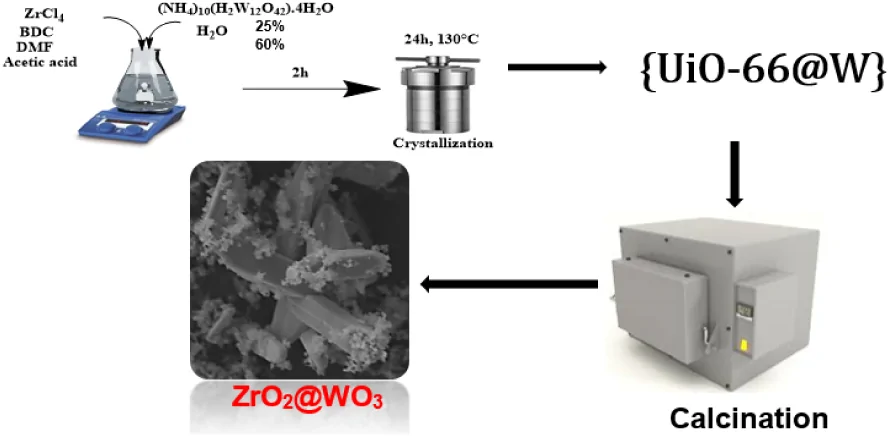
Synthesis of ZrO_2_@WO_3_

#### 2.2.2 Photocatalytic Activity of ZrO_2_, ZrO_2_@WO_3_-25 and ZrO_2_@WO_3_-60

Photocatalytic activity was evaluated in terms of the degradation of IC and MB in ZrO_2_, ZrO_2_@WO_3_-25 and ZrO_2_@WO_3_-60. Experiments were conducted in a photochemical reactor containing 70 mL of an IC and MB solution (10–20 mg/L) at a pH of 4–8 and with 30–70 mg of photocatalyst. The adsorption-desorption equilibrium of the dyes on the photocatalysts was achieved by stirring the solution in the dark for 30 min prior to placing it in the photochemical reactor. The distance between the lamp and the reactor was fixed at 35 cm, after irradiating the solution at room temperature using a xenon lamp equipped with a light source simulating sunlight (495 W/m^2^) for a set period, 8 mL of the solution were sampled at regular intervals. These samples were then filtered and analyzed using a UV–visible spectrophotometer at maximum wavelengths (λ_max_) of 610 and 666 nm for IC and MB, respectively. The degradation rate (R%) of IC and MB was determined using eq 1 The same procedure was used to evaluate the influence of parameters that may affect the photocatalytic activity of ZrO_2_, ZrO_2_@WO_3_-25, and ZrO_2_@WO_3_-60.

1 $$R\left(\%\right)=\frac{A_{0}-A_{t}}{A_{0}}\times 100$$

Where, A_0_ and A_t_ are the initial and the equilibrium IC and MB absorbance.

#### 2.2.3 Degradation Kinetics

First- and second-order kinetic models were used to evaluate the degradation kinetics of the IC and MB dyes. The linear forms are given by eqs 2 and 3, respectively.^9^

2 $$\ln\frac{C_{0}}{C_{t}}=k_{1}t$$

3 $$\frac{1}{C_{t}}-\frac{1}{C_{0}}=k_{2}t$$

Where C_0_ (mg/L) is the initial dye concentration (mg/L) and Ct (mg/L) is the irradiated dye concentration (mg/L) at time t, *k*_1_ and *k*_2_ are the first- and second-order rate constants (min^–1^), and t is time (min).

#### 2.2.4 Mathematical Modeling of the Optimization of Degradation by the ZrO_2_@WO_3_-25 and ZrO_2_@WO_3_-60 Hybrid Oxides

The central composite design (CCD) mathematical model^31^ was used to optimize the degradation of IC and MB dyes on the hybrid oxide in order to evaluate the synergistic effects of the factors involved in this process simultaneously. Design-Expert 13 and Statgraphics Centurion 19 Supplemental software were used to generate the experimental matrix. The following factors were used: A: amount of catalysts (30–70 mg); B: time (50–110 min); C: pH (4–8); and D: dye concentration (10–20 mg/L) (see Table 1). eq 4 represents the number of experiments (N) to be generated from the model.

4 $$N=n_{c}+2n+2^{n}=24+2\left(4\right)+4=28$$

**Table 1:** Experimental Independent Variable

Factor	Units	Coded variable	Variables levels		
Mass of composite	mg	A	30.0	50.0	70.0
Time	min	B	50.0	80.0	110.0
pH	/	C	4.0	6.0	8.0
Concentration	mg/L	D	10.0	15.0	20.0

Where N is the total number of experiments and n_c_ is the number of central points, 2n and 2^n^ are the number of trials in the star designs and the factorial design, respectively. Table 3 presents the results of all 28 experiments.

#### 2.2.5 Model Validation and Analysis of Variance

An empirical model based on second-order polynomial regression (eq 5) was developed to predict the expected response (percentage degradation), *η*%).^32^

5 $$\eta =b_{0}+\overset{n}{\underset{i=1}{\sum }}b_{i}X_{i}+\overset{n}{\underset{i=1}{\sum }}b_{ii}X_{i}^{2}+\overset{n}{\underset{i=1}{\sum }}\overset{n}{\underset{j>1}{\sum }}b_{ij}X_{i}X_{j}$$

*η* is the degradation effiency, bo and bi are the constant of the quadratic and linear coefficients of the interaction variable, bii and bij are the coefficients of quadratic and interaction between the variables, xi and xj are the coded variables of factors during the degradation. The validation of the model was based on the regression coefficient (R^2^) and the adjusted coefficient of determination (R^2^_adj_), as well as the p-value derived from the variance data (ANOVA) during the degradation of IC and MB dyes by ZrO_2_@WO_3_-25 and ZrO_2_@WO_3_-60, with a 95% confidence interval. Design-Expert 13 software was used to generate 2D and 3D contour plots considering the simultaneous interactions of the four factors.

#### 2.2.6 DFT Study

The Material Studio software (BIOVIA, Inc.) was used for the Monte Carlo molecular modeling. The IC and MB dye structures were drawn and optimized using the DMol3 module and the GGA/PW91 functional. The localization of nucleophiles and electrophiles was determined by calculating the energies of the least (LUMO) and most (HOMO) occupied molecular orbitals, as well as the energy gap (E_gap_).^33,34^ The Fukui indices for electrophilic (*f* ^–^), nucleophilic (*f* ^*+*^) and radical (*f* ^*0*^) attacks were then determined.^35^

#### 2.2.7 Electrochemical Measurements

For the electrochemical measurements, a conventional three-electrode system consisting of a working electrode (bare GCE), an auxiliary electrode (platinum), and a reference electrode (Ag/AgCl (3 M KCl)) connected to the PalmSens3 potentiostat, was used during the measurement. PSTrace software, incorporated into the potentiostat was used to record electrochemical experimental curves, and Origin 9.0 software was used to plot the curves. 5 mL of 5 mM [Fe­(CN)_6_]^3–^ /^4 –^ (0.1 M KCl, pH 7.0) was used as the electrolyte for the EIS and CV measurements. CV was recorded at a potential ranging from −0.4 to 0.8 V, and EIS was recorded at a potential of 0.4 V in the frequency range from 1.0 kHz to 100.0 kHz.

#### 2.2.8 Characterization Method

See Supporting Information.

## 3 Results and Discussion

### 3.1 XRD, FTIR, and N_2_ Sorption Analysis of Zr-UiO-66, Zr/W-UiO-66, ZrO_2_, ZrO_2_@WO_3_-25 and ZrO_2_@WO_3_-60

XRD analyses of Zr-UiO-66 and Zr/W-UiO-66 were performed to confirm the synthesis of the precursor materials (see Figure 1a). The formation of Zr-UiO-66 is confirmed by the presence of standard reference peaks at 2θ = 7 and 9, which indicate the formation of 3D structures interconnected by Zr_6_O_4_(OH)_4_ and BDC bonds with narrow 6 Å windows, similar to the simulated material.^36^ Similar observations were reported by Nguena et al. in 2025.^37^ However, the precursor samples (Zr/W-UiO-66–25 and 60), which are doped with 25% and 60% tungsten, respectively, also exhibit peaks similar to those of Zr-UiO-66. However, it can be seen that the intensity of the Bragg diffraction peaks decreases with increasing tungsten concentration due to variation in tungsten’s electron density in the Zr/W-UiO-66–25% and 60% samples. These observations demonstrate the validity of the method used to incorporate tungsten in situ into the 3D lattice of the Zr-UiO-66 material, forming W–O bonds on Zr_6_O_4_(OH)_4_.^38^ Figure 1b shows the XRD patterns obtained after thermal degradation. These patterns reveal the formation of tetragonal ZrO_2_ (t-ZrO_2_) at 2θ = 24.5° and 35.5°,^39^ and monoclinic ZrO_2_ (m-ZrO_2_) at 2θ = 28.5° and 32.5°. They also show ZrO_2_@WO_3_-25 and ZrO_2_@WO_3_-60, as evidenced by Bragg diffraction peaks that correspond to the hexagonal WO_3_ phases at 2θ = 17.5° and 27.5°,^40,41^ as well as the monoclinic WO_3_ phase at 2θ = 23–24°.^40,42^ Doping the Zr-UiO-66 material with tungsten induces a structural transformation of the ZrO_2_ from the monoclinic to the tetragonal phase due to the stabilization of the tetragonal phase. The sample doped with 60% tungsten exhibits more intense WO_3_ peaks, indicating high crystallinity and strong tungsten dispersion in the doped samples. This correlates with the XRD analyses presented in Figure 1a.

**Figure 1:**
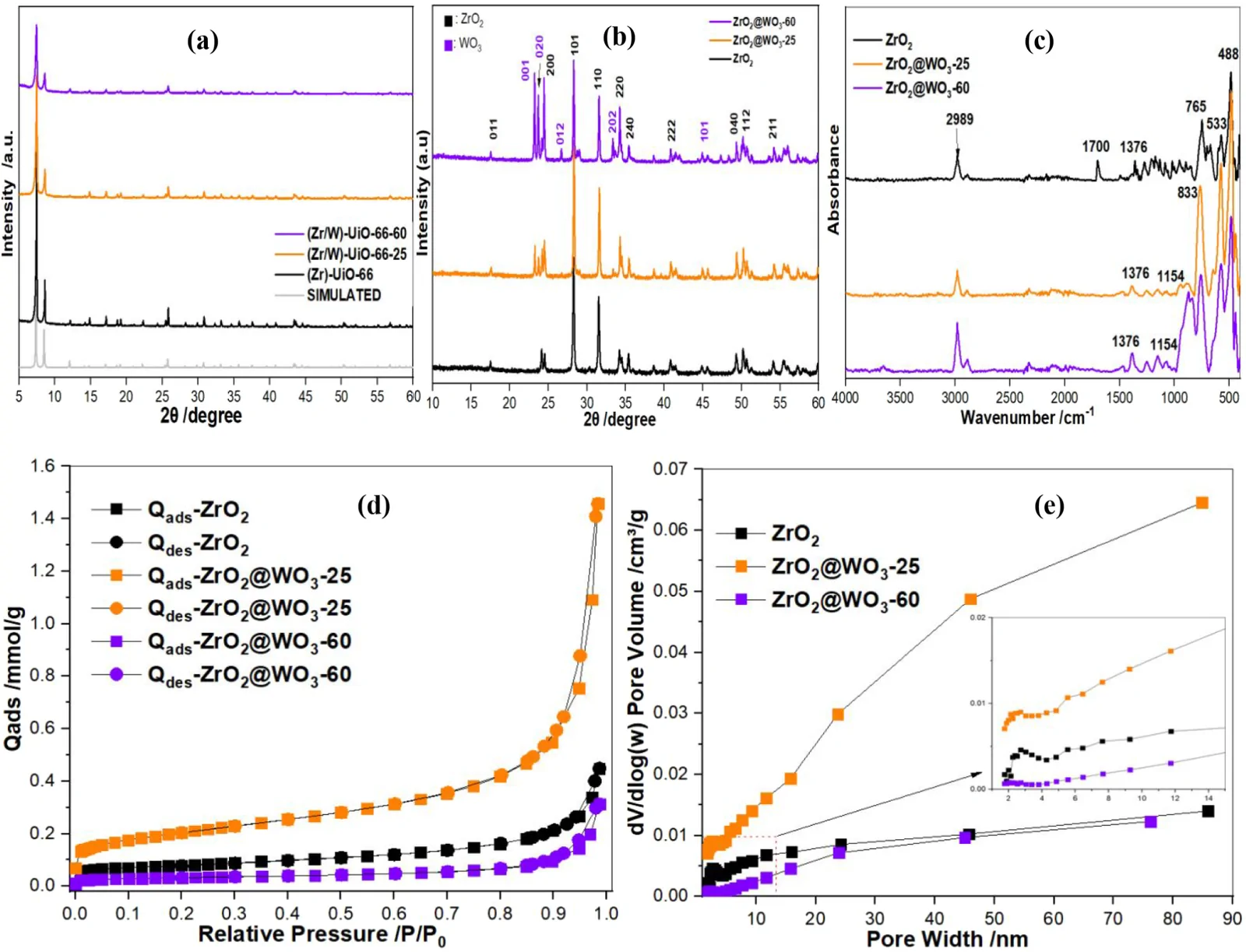
XRD patterns of Zr-UiO-66 and Zr/W-UiO-66 (a), ZrO_2_ ZrO_2_@WO_3_-25 and ZrO_2_@WO_3_-60 samples (b), FTIR spectra (c), N_2_ sorption isotherm (d), and pore size distribution (e).

Figure 1c shows the FTIR spectra obtained from the ZrO_2_, ZrO_2_@WO_3_-25 and ZrO_2_@WO_3_-60 samples. For ZrO_2_, the formation of tetragonal and monoclinic oxides can be seen from the presence of vibrational bands at 765 and 533 cm^–1^, which correspond to the Zr–O and Zr–O–C vibrational modes.^43,44^ The carbonyl group of the BDC linker is evident from the band at 1700 cm^–1^. The stretching vibrations of the O–H bonds in the absorbed water molecules are visible at 2989 cm^–1^ in all three samples. The 1376 cm^–1^ band present in all samples is attributable to the Zr–O–W and O–Zr–W stretching modes of the hybrid oxides.^45,46^ The formation of these hybrid oxides is confirmed by new absorption bands appearing at 1154 and 833 cm^–1^. These bands are more intense in the ZrO_2_@WO_3_-60 sample and are attributable to the O–W–O and W=O bonds present in hexagonal and monoclinic WO_3_, respectively.^25^ The intense bands at 488 and 533 cm^–1^ are due to the adsorption bands of the Zr–O, W=O and Zr–O–W bonds in the tetragonal phases, which indicates high tungsten dispersion with strong bonding in the doped samples. These vibrational modes indicate the formation of hybrid oxides containing well-dispersed, uniform WO_3_ nanoparticles, as confirmed by XRD and SEM analyses presented in the following section. Figure 1(d and e) show the nitrogen adsorption/desorption isotherms and the pore size distribution of the BHJ, respectively. As shown in Figure 1d, all of the obtained isotherms can be classified as type III, according to the IUPAC classification.^40^ The presence of a hysteresis loop indicates the presence of mesoporous and microporous features within the samples, while the absence of monolayer coverage at low relative pressures (P/P_0_ < 0.2) suggests weak adsorbate-adsorbent interactions. Nevertheless, favorable interactions between N_2_ molecules and the most active sites on the oxide surfaces contribute to the observed adsorption behavior. The BET surface areas obtained were: 6.15 m^2^ /g for the ZrO_2_ sample; 15.93 m^2^ /g for the ZrO_2_@WO_3_-25 sample; and 2.53 m^2^ /g for the ZrO_2_@WO_3_-60 sample. The total pore volume was found to be 0.009837, 0.026539, and 0.005081 cm^3^ /g for the ZrO_2_, ZrO_2_@WO_3_-25 and ZrO_2_@WO_3_-60 samples, respectively, and the micropore volume was found to be 0.000754, 0.000992, and 0.000290 cm^3^ /g for the ZrO_2_, ZrO_2_@WO_3_-25 and ZrO_2_@WO_3_-60 samples, respectively. The ZrO_2_@WO_3_-60 sample exhibited the lowest BET surface area and total pore volume, likely due to the high W concentration, which obstructs the pores of the hybrid oxides by preventing N_2_ molecule penetration. The pore distribution shows a maximum pore volume with a pore width of approximately 3 nm (see Figure 1e). Nevertheless, the values obtained are consistent with those reported in previous studies.^41^

### 3.2 SEM/EDX Analysis of ZrO_2_, ZrO_2_@WO_3_-25 and ZrO_2_@WO_3_-60 Samples

Figure 2a, b and c show the results of SEM analyses of the ZrO_2_, ZrO_2_@WO_3_-25 and ZrO_2_@WO_3_-60 oxide samples. Figure 2a shows the formation of uniform ZrO_2_ nanoparticles,^42^ which is responsible for the formation of the Zr-UiO-66 material through bonding between the −COOH groups of the BDC linker and the Zr_6_O_4_(OH)_4_ clusters. However, Figure 2b and 2c show that the ZrO_2_@WO_3_-25 and ZrO_2_@WO_3_-60 samples have different morphologies to ZrO_2_, with homogeneous ZrO_2_ dispersion in the samples and hexagonal particles. This confirms the formation of ZrO_2_@WO_3_ hydride oxides.^41,45^ The hexagonal and monoclinic WO_3_ phases are observable due to the presence of more visible particles in Figure 2b. These observations indicate good tungsten dispersion in the doped samples and the formation of Zr–O–W bonds.^43^ Adding W to the cavities of the three-dimensional Zr-UiO-66 lattice inhibits the growth of octahedral units.^38^ This could explain the homogeneous distribution of ZrO_2_ in the ZrO_2_@WO_3_-25 and ZrO_2_@WO_3_-60 samples. EDX and EDX mapping analyses (Figure 2d) of the ZrO_2_@WO_3_-25 sample and Figure S1 of the ZrO_2_@WO_3_-60 sample, performed after heat treatment, show the presence of C, O and Zr in the Zr-UiO-66 samples and of C, O, Zr and W in the Zr/W-UiO-66 samples. This confirms the presence of W in the obtained samples, the presence of carbon stems from the decomposition of BDC into ash. The elemental compositions obtained from the EDX analysis are approximately 4% C, 32% O and 64% Zr for pristine ZrO_2_; 3% C, 20% O, 58% Zr and 19% W for ZrO_2_@WO_3_-25; and 4% C, 22% O, 46% Zr and 28% W for ZrO_2_@WO_3_-60 (see Figure S1). The increase in tungsten concentration is confirmed by the high W content of ZrO_2_@WO_3_-60, which is comparable to that of ZrO_2_@WO_3_-25. EDX mapping analysis shows that tungsten is uniformly dispersed throughout the ZrO_2_ using the in situ approach and the solvothermal method.

**Figure 2:**
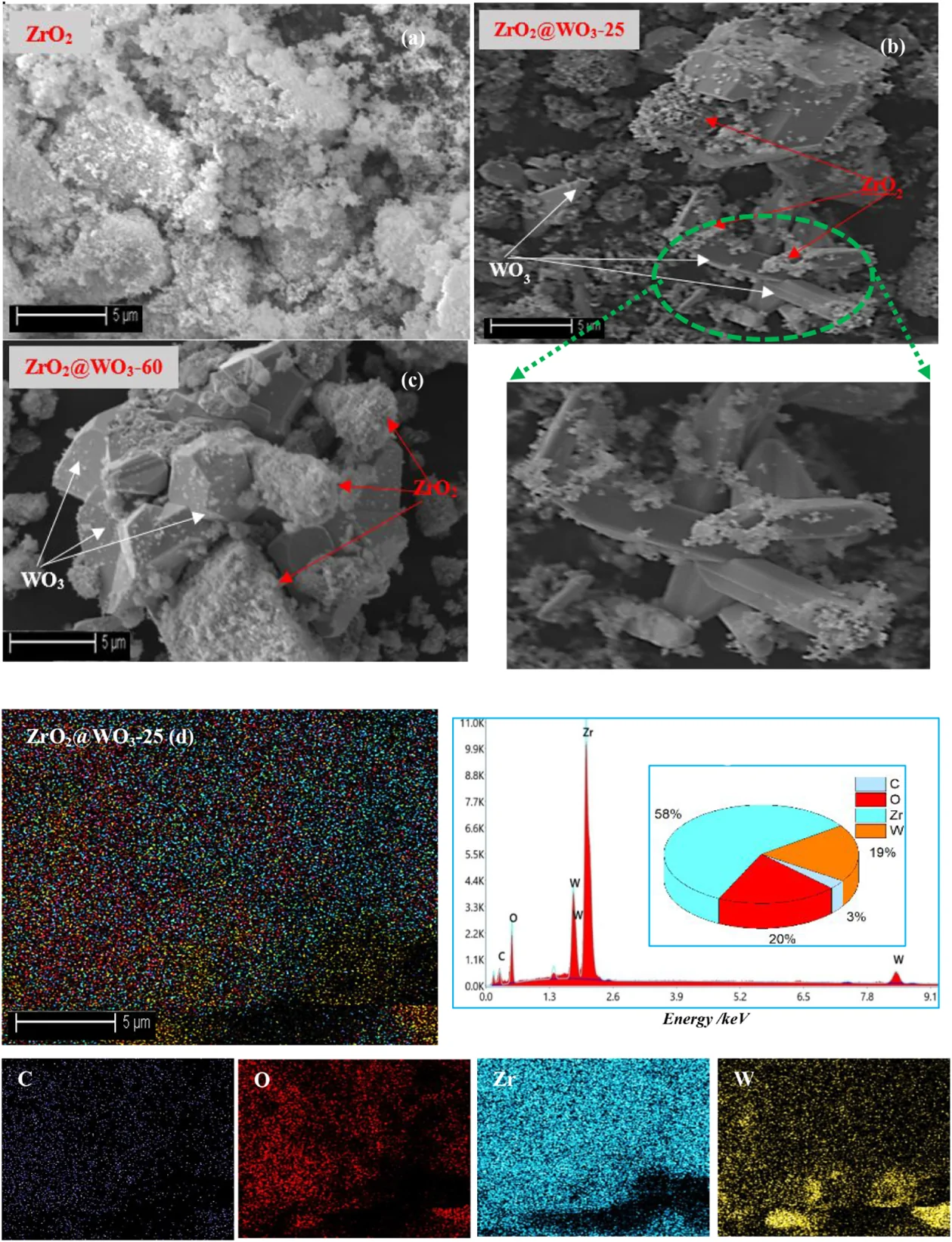
SEM analysis of ZrO_2_ (a), ZrO_2_@WO_3_-25 (b), ZrO_2_@WO_3_-60 (c), EDX/mapping and EDX of ZrO_2_@WO_3_-25 (d).

### 3.3 XPS Analysis of ZrO_2_, ZrO_2_@WO_3_-25 and ZrO_2_@WO_3_-60 and TGA/DTG Analysis

Figure 3 shows the XPS spectra of ZrO_2_, ZrO_2_@WO_3_-25 and ZrO_2_@WO_3_-60. The full ZrO_2_ spectrum shows the presence of C 1s, O 1s and Zr 3s, p and d. The presence of C 1s is due to the decomposition of the BDC linker. In addition to W 4­(d and f), the XPS spectra of ZrO_2_@WO_3_-25 and ZrO_2_@WO_3_-60 show the elements observed in ZrO_2_. The simultaneous presence of Zr and O in all spectra confirms the formation of ZrO_2_@WO_3_ hydride oxides.^39^ The Zr 3s spectrum exhibits peaks at 434, 438, 442, and 443 eV, respectively, which are attributable to Zr–O, Zr, Zr–O–W and Zr–C. The Zr 3d spectrum exhibits peaks at 182, 187, 192, and 193 eV, which are attributable to Zr–C, Zr–O, Zr 3d_3_/_2_ and Zr 3d_5_/_2_ and confirm the presence of the tetragonal (t′-ZrO_2_) and monoclinic (m-ZrO_2_) phases in the ZrO_2_ nanoparticles.^44^ The W 4d spectrum exhibits groups at ∼252 eV (W 4d5), ∼258 eV (O–W 4d5), ∼442 eV (O–W 4d3), and ∼443 eV (W 4d3). The W 4f spectrum exhibits peaks at ∼32, ∼37, ∼42 and ∼43 eV, which are attributable to Zr­(4p)–O-W, W­(4p)-O, O–W 4f and Zr–O–W 4f, respectively. These observations indicate the good dispersion of tungsten in the doped samples and the formation of Zr–O–W bonds containing the hexagonal and monoclinic WO_3_ phases.^45^

**Figure 3:**
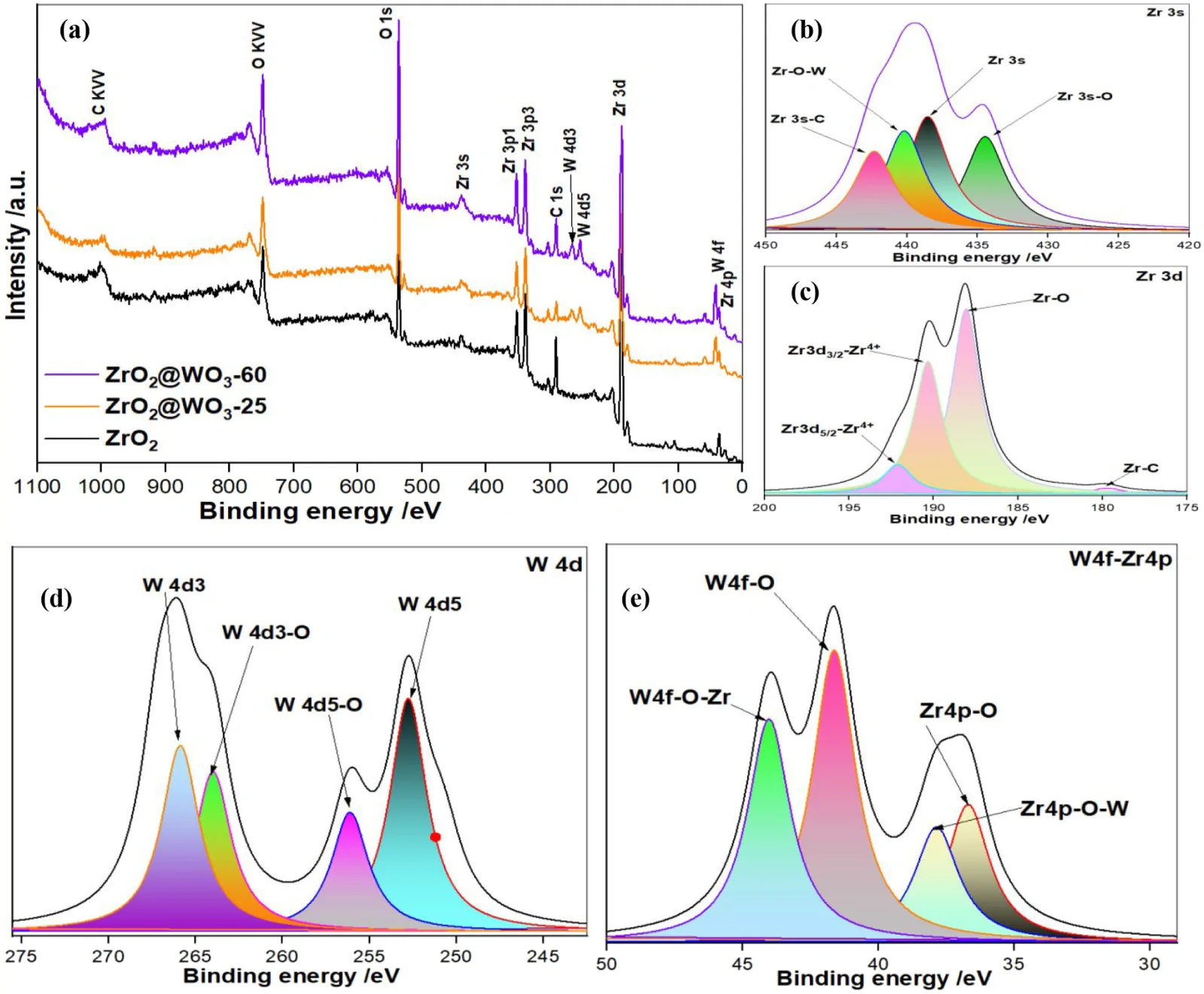
XPS survey spectrum of ZrO_2_, ZrO_2_@WO_3_-25 and ZrO_2_@WO_3_-60 (a), Zr 3s (b) and 3p (c), W 4d (d) and 4f (e).

Thermogravimetric analysis was performed on the Zr-UiO-66 and Zr/W-UiO-66 precursor materials. Figure S2 shows the results obtained. It can be seen that the TGA profile of the thermal decomposition of Zr-UiO-66 occurs in two distinct steps with temperature ranges of 50–390 °C and 390–750 °C for steps 1 and 2, respectively, which are comparable to the three steps observed in Zr/W-UiO-66 with temperature ranges of 50–390 °C, 390–750 °C and 750–1000 °C for steps 1, 2 and 3, respectively.^46^ The first mass loss in the 50–390 °C range in the Zr-UiO-66 profile is due to the removal of absorbed molecules, intra-framework DMF and solvent. The second mass loss in the 390–750 °C range is due to the destruction of the carboxylate ligand that connects the zirconium clusters, as well as the dehydration of Zr_6_O_4_(OH)_4_. The formation of secondary Zr_6_O_6_ subunits and their transformation into ZrO_2_ becomes observable at temperatures above 650 °C.^50,51^ For Zr/W-UiO-66 samples, steps 1 and 2 correspond to the loss of solvent molecules and the dehydroxylation of Zr_6_O_4_(OH)_4_, which is similar to what was observed in the Zr-UiO-66 sample. However, the third mass loss in the 750–1000 °C range is due to the decomposition of W_12_O_40_ into WO_3_, as well as the formation of Zr–O–W hybrid oxides. The presence of W in the Zr/W-UiO-66 samples becomes evident at this final stage of mass loss. The difference in mass loss between the Zr/W-UiO-66–25 and Zr/W-UiO-66–60 samples is due to the difference in W weight between the two samples. Each mass loss event is accompanied by framework destruction and complete combustion of the DBC carbon linkers.^23,50^

### 3.4 Raman, EIS, UV–vis DRS, and Photoluminescence (PL) Analysis

The Raman analyses are shown in Figure 4a. The ZrO_2_ Raman spectrum exhibits several peaks characteristic of the monoclinic, cubic and tetragonal phases with varying degrees of tetragonality. The peak at 610–650 cm^–1^ is attributable to the cubic phase (c-ZrO_2_). The monoclinic phase (m-ZrO_2_) can be identified by Raman shifts at 350–400 cm^–1^ and 160–190 cm^–1^, which are characteristic of zirconium nanoparticles. The tetragonal phases (t-ZrO_2_) are observable between 100 and 310 cm^–1^, and the bands at 488 cm^–1^ are characteristic of tetragonal phases with varying degrees of tetragonality (t′-ZrO_2_). Similar observations have been reported in the literature.^41^ For instance, Borik et al. (2017) demonstrated the presence of t″-ZrO_2_ phases based on peaks observed at 490 cm^–1^.^47^ The Raman spectra of the ZrO_2_@WO_3_-25 and ZrO_2_@WO_3_-60 samples reveal several Raman bands resulting from the bonding of tungsten to bridging oxygen and zirconium.^39^ The weak bands at 85 cm^–1^ are attributable to low-symmetry WO_3_ and Zr–O–W phases, while the bands at 150 cm^–1^ are due to collective rotation of tetragonal W–W units.^45^ The bands appearing at 290–320 cm^–1^ are attributable to the υ­(O–W–O) deformation and δ­(W–O–W) bending modes. The δ­(O–W–O) stretching modes have Raman bands at 340 cm^–1^. The intense bands at 750 and 815 cm^–1^ are characteristic of the tetragonal and monoclinic phases of crystalline WO_3_, and correspond to the stretching modes of the bridging oxygen atoms. These bands are attributable to the υ­(W_2_O_6_) and υ­(W–O–W) stretching modes.^48^ The presence of these Raman bands is due to the distortion of the Zr–O–W phases present in the hybrid oxides.^39^ The low intensity of the tetragonal (t′-ZrO_2_) and monoclinic (m-ZrO_2_) phases in ZrO_2_@WO_3_-60 can be explained by the high concentration of W.

**Figure 4:**
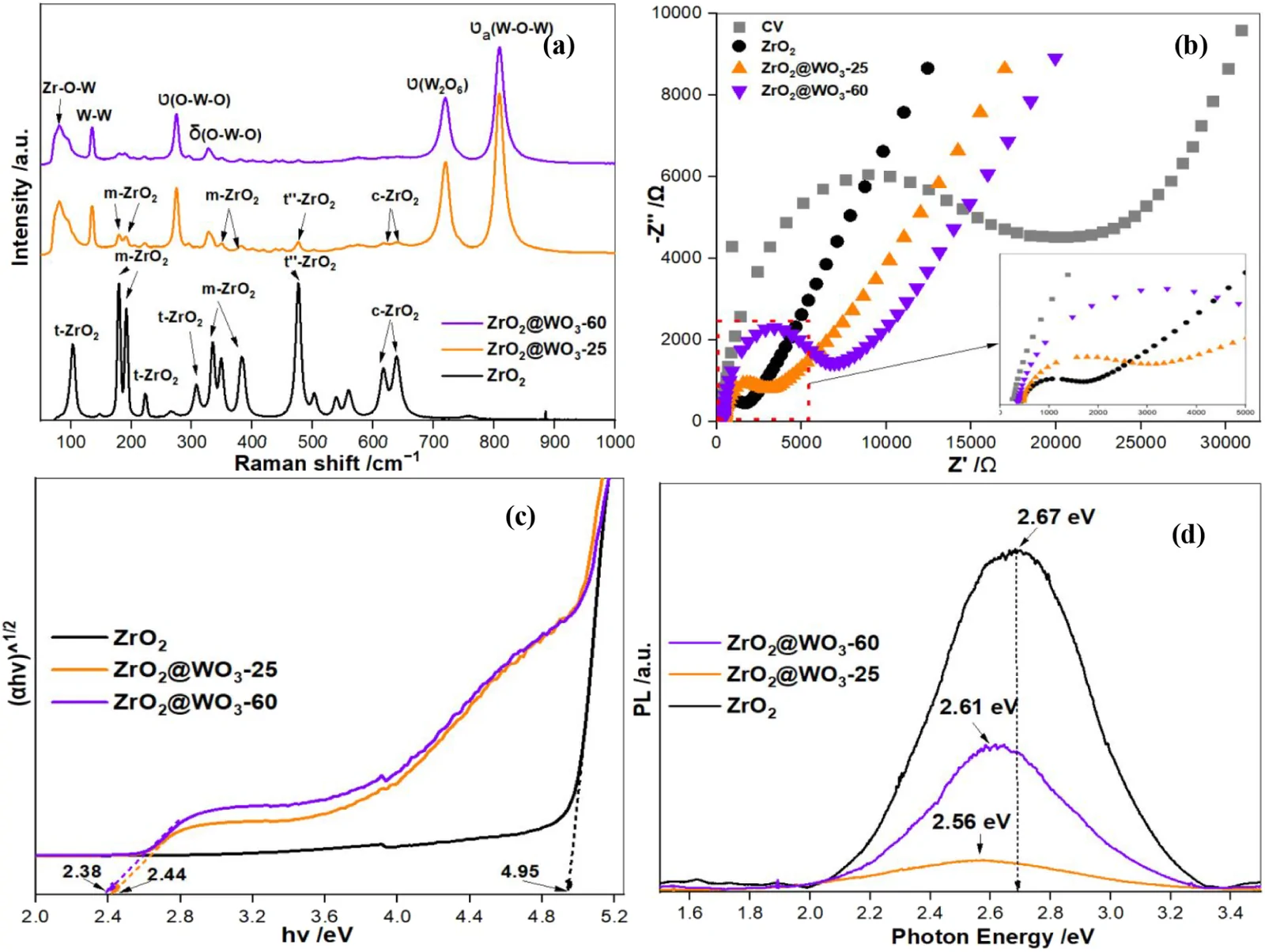
Raman (a) EIS (b), UV–vis DRS (c) and photoluminescence (PL) (d) analysis.

Electrochemical impedance spectroscopy (EIS) was performed to evaluate the dynamic charge transfer performance of the samples. The Nyquist plot is shown in Figure 4b, where Z′ represents the real part of the impedance and Z″ the imaginary part. ZSimpWin software was used to determine the transfer resistance (R_ct_) based on the radius of the semicircles corresponding to charge transfer at the ZrO_2_, ZrO_2_@WO_3_-25 and ZrO_2_@WO_3_-60 oxide interfaces, as well as the bare glassy carbon (CE) electrode.^9^ The values obtained were 18214.28 Ω for the CE, 1066.67 Ω for ZrO_2_, 2533.33 Ω for ZrO_2_@WO_3_-25 and 5400.44 Ω for ZrO_2_@WO_3_-60. While the hybrid oxides exhibit higher R_ct_ values than ZrO_2_, the high R_ct_ value of ZrO_2_@WO_3_-60 is comparable to that of ZrO_2_@WO_3_-25, which can be attributed to the high tungsten concentration. Lower R_ct_ values indicate faster charge transfer from the sample to the oxide-electrolyte interface, which could limit recombination and enhance photocatalytic reactions.^49^

Figure 4c shows the bandgap energy (E_g_) of the ZrO_2_, ZrO_2_@WO_3_-25 and ZrO_2_@WO_3_-60 samples. These were calculated using Tauc’s equation (αhν = (hν – E_g_)^n^), where α, h, ν, E_g_ and n represent the absorption coefficient, Planck’s constant, frequency, bandgap and constant, respectively. For directly allowed transitions, n = 0.5. The obtained bandgap energies were 4.95 eV for ZrO_2_, 2.44 eV for ZrO_2_@WO_3_-25, and 2.38 eV for ZrO_2_@WO_3_-60, as determined by fitting a tangent to the linear portion of the curve plotted from the Tauc equation. ZrO_2_ is known to have a bandgap energy ranging from 4.0 to 5.5 eV,^41^ due to surface deformation and increased quantum confinement of the sample.^23^ The bandgap value obtained for ZrO_2_ falls within this range and is close to the value reported by Vinayagam et al. in 2022 (4.9 eV) for zirconium nanoparticles. The ZrO_2_, ZrO_2_@WO_3_-25 and ZrO_2_@WO_3_-60 samples exhibit lower E_g_ values than the ZrO_2_ sample due to the presence of W. Surakasi et al. (2022) obtained an E_g_ value of 2.4 eV for WO_3_. Samples with bandgap values between 1.3 and 3.5 eV are known to exhibit better energy efficiency.^30^ The low Eg values obtained indicate that the synthesized hybrid oxides exhibit better photocatalytic performance. This suggests that the ZrO_2_@WO_3_-25 and ZrO_2_@WO_3_-60 samples can generate the free radicals necessary for redox reactions.^50^

Figure 4d shows the photoluminescence (PL) spectrum recorded at an excitation wavelength of 523 nm. Three emission bands are observed, corresponding to energies of 2.67 eV (ZrO_2_), 2.56 eV (ZrO_2_@WO_3_-25) and 2.61 eV (ZrO_2_@WO_3_-60), and to wavelengths of 464, 475, and 484 nm, respectively. This difference is explained by the energy gap between the emission spectra, which is likely due to oxygen vacancies present between the conduction and valence bands. These modify the electronic structure of the materials and influence the radiative recombination of photogenerated charge carriers. Similar results were obtained by Vinayagam et al. (2022).^41^ The ZrO_2_@WO_3_-25 and ZrO_2_@WO_3_-60 samples exhibit small PL emission spectra due to the presence of tungsten. These observations demonstrate that, at high tungsten concentrations, tungsten induces structural modification of monoclinic ZrO_2_, stabilizing the tetragonal phase.

### 3.5 Analysis of Variance for the Degradation (R) of IC and MB

A central composite design was used for mathematical modeling to optimize the factors affecting the degradation of IC and MB dyes. ANOVA analyses, presented inTable [Table tbl2]a–b for IC and MB dyes respectively, account for composite mass (factor A), time (factor B), pH (factor C) and concentration (factor D). The ANOVA table for IC degradation on ZrO_2_@WO_3_-60 (Table 2a) shows that each factor (A, B, C and D) significantly influences IC degradation due to P-values <0.05.^13^ Furthermore, the combined effect of the factors exhibits the same results. The effects of the factors presented in the ANOVA tables account for R^2^ = 97.70% and R^2^_adj_ = 95.22%, as well as degrees of freedom and P-values at a 95% confidence level. Significant correlations between factors A, B, C and D, with p-values <0.05, as well as R^2^ and R^2^_adj_ close to unity and a perfectly orthogonal design, indicate the reliability of the adopted mathematical model. The model shows that the degradation efficiency of IC and MB dyes is significantly affected by composite mass, time, pH and concentration. These observations are supported by F-ratios greater than 5 for the individual (A, B, C and D) and combined (AA, BB, CC and DD) factors.^51^ Similar behaviors are observed for the MB dye using ZrO_2_@WO_3_-25 (see Table 2b). The R^2^ statistic indicates that the model explains 98.73% of the variability in degradation. The adjusted R^2^ (97.38%) is more suitable for comparing models with different numbers of independent variables. The ZrO_2_–W–25 and ZrO_2_–W–60 photocatalysts can generate the •O_2_ and •OH free radicals required for redox reactions due to oxygen gaps in the conduction and valence bands of the oxides. All these factors were necessary for the effective degradation of the IC and MB dyes.

**Table 2:** Analysis of Variance (ANOVA)

Source/ZrO_2_@WO_3_-60 (a)	Sum of Squares	Df	Mean Square	F-Ratio	P-Value
**A: Mass of composite**	165.01	1	165.01	29.20	0.0001
**B: Time**	396.39	1	396.39	70.14	0.0000
**C: pH**	299.31	1	299.31	52.96	0.0000
**D: Concentration**	349.01	1	349.01	61.76	0.0000
**AA**	0.05	1	0.05	0.01	0.9231
**AB**	78.67	1	78.67	13.92	0.0025
**AC**	35.28	1	35.28	6.24	0.0267
**AD**	117.29	1	117.29	20.75	0.0005
**BB**	9.516	1	9.51	1.8	0.2170
**BC**	30.86	1	30.86	5.46	0.0361
**BD**	90.92	1	90.92	16.09	0.0015
**CC**	473.19	1	473.19	83.73	0.0000
**CD**	47.68	1	47.68	8.44	0.0123
**DD**	47.35	1	47.35	8.38	0.0125
**Total error**	73.46	13	5.64		
**Total (corr.)**	3195.21	27			
R^2^ = 97.70					
R^2^ (adjusted) = 95.22					

Source/ZrO_2_@WO_3_-25 (b)	Sum of Squares	Df	Mean Square	F-Ratio	P-Value
**A: Mass of composite**	224.51	1	224.51	43.97	0.0000
**B: Time**	1339.55	1	1339.55	262.36	0.0000
**C: pH**	27.75	1	27.75	5.44	0.0365
**D: Concentration**	279.18	1	279.18	54.68	0.0000
**AA**	0.39	1	0.39	0.08	0.7841
**AB**	8.35	1	8.35	1.64	0.2233
**AC**	56.77	1	56.77	11.12	0.0054
**AD**	0.94	1	0.94	0.18	0.6748
**BB**	944.11	1	944.11	184.91	0.0000
**BC**	5.47	1	5.47	1.07	0.3193
**BD**	135.61	1	135.61	26.56	0.0002
**CC**	50.08	1	50.08	9.81	0.0079
**CD**	28.52	1	28.52	5.58	0.0344
**DD**	0.18	1	0.18	0.04	0.8543
**Total error**	66.37	13	5.12		
**Total (corr.)**	5262.84	27			
R^2^ = 98.73					
R^2^ (adjusted) = 97.38					

#### 3.5.1 Model Validation and Regression Coefficients

Pareto charts (Figure 5a and 5b) were used to highlight the statistically significant effects of the individual and combined variables (A, B, C and D). Figure 5a and 5b show that, while composite mass and time have a positive effect on the degradation performance of IC and MB dyes, concentration and pH have a negative effect. However, the combined effect of these factors is more negative than positive. High composite levels allow for better dye adsorption onto the material’s surface, while creating oxygen voids that are responsible for light absorption and electron transfer from the conduction band to the valence band.^52^ This induces efficient recombination of photo-generated electron-hole pairs. Similarly, longer reaction times enhance the photocatalyst’s photocatalytic activity; longer reaction times allow for optimal absorption of visible light during the irradiation period of the material’s surface and the dyes, thanks to an increased energy supply to the system during adsorption and increased adsorption kinetics.^53^ In contrast, pH tends to reduce degradation efficiency. At basic pH values, the dye molecules are anionic, leading to electrostatic repulsion between the OH^–^ ions of the ZrO_2_@WO_3_-25/60 hybrid oxides and the anionic dyes. Regression models adjusted for the linear and quadratic effects of each factor on the degradation efficiency of IC and MB dyes on ZrO_2_@WO_3_-25/60 hybrid oxides are represented by polynomial equations,^31^ (eqs 6 and 7 for IC and MB, respectively).

6 $$R\left(\%\right)=-120.006+1.03951\times A+0.377012\times B+39.7126\times C+5.84984\times D+0.000364221\times A^{2}-0.00369583\times A\times B-0.037125\times A\times C-0.027075\times A\times D+0.0021341\times B^{2}-0.0231458\times B\times C-0.0158917\times B\times D-3.38608\times C^{2}+0.172625\times C\times D-0.171372\times D^{2}$$

7 $$R\left(\%\right)=-121.573+0.22805\times A+3.86112\times B+12.1707\times C-0.473511\times D+0.000983877\times A^{2}+0.00120417\times A\times B-0.0470937\times A\times C+0.002425\times A\times D-0.0212572\times B^{2}+0.00975\times B\times C-0.0194083\times B\times D-1.10161\times C^{2}+0.1335\times C\times D+0.010542\times D^{2}$$

**Figure 5:**
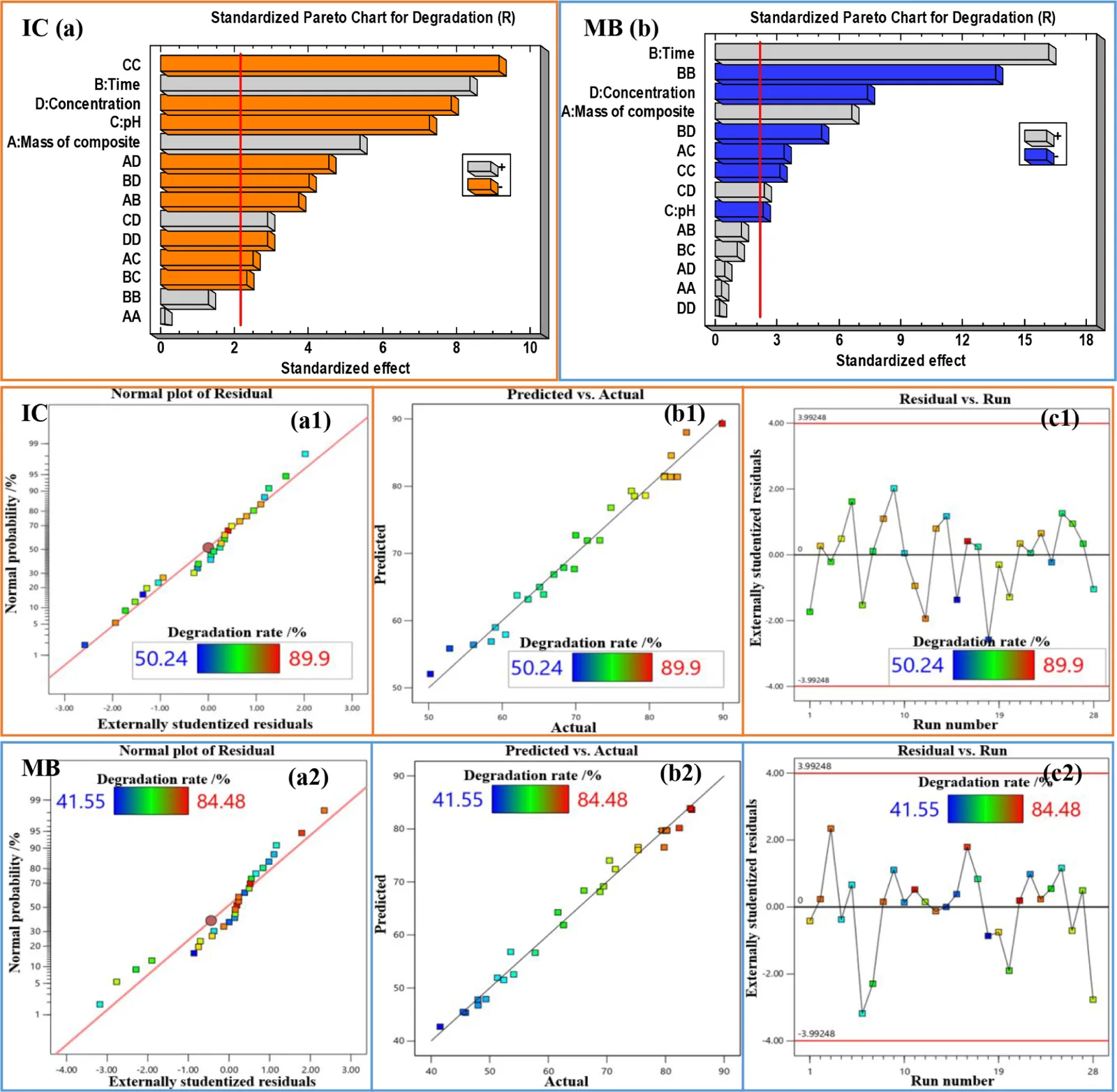
Pareto chart showing the effect of factors and interactions (a and b), normal plot of residuals (a1 and a2), predicted vs. actual (b1 and b2), and residual vs. run (c1 and c2) for the degradation (%) of IC and MB, respectively.

Table 3 shows the degradation efficiency (observed value, adjusted value and residual) for each experimental condition and dye. The mathematical model shows that an IC degradation rate of 95.11% is achieved under optimal conditions: a composite mass of 59.9 mg, a time of 110 minutes, a pH of 6.06 and a concentration of 10 ppm. For MB, the degradation rate is 92.82% under optimal conditions: a composite mass of 65.1 mg, a time of 109 minutes, a pH of 5.96 and a concentration of 10 ppm. The model’s validity is confirmed by the agreement between the experimentally obtained degradation yield for IC (95.11%) and the model’s prediction (97.01%), as well as for MB (92.82%) and the model’s prediction (96.02%) under the experimental conditions. These observations are supported by the low values of the residuals of the degradation yield (see Table 3), as well as by the correlation between the normal probability distribution of the residuals see Figure 5a1 and a2: predicted vs. actual (**b1 and b2**), and residual vs. stroke (**c1 and c2**)). The effect of the factor is also evident in Figures S3 and S4: residual vs. predicted; residual vs. composite mass; Cook’s distance; leverage vs. run; and DFBETAS for the intercept vs. run. These figures relate to the degradation of the IC and MB, respectively.

**Table 3:** Experimental Design Matrix and Degradation Rate

Factor					Degradation (R) %					
					MB			IC		
Run	A	B	C	D	Observed	Fitted	Residual	Observed	Fitted	Residual
**1**	50.0	80.0	6.0	15.0	79.42	79.6809	–0.2609	83.20	81.3936	1.8064
**2**	30.0	50.0	4.0	10.0	49.36	47.9117	1.4483	60.46	57.9356	2.5244
**3**	70.0	50.0	4.0	10.0	53.59	56.8126	–3.2226	74.79	76.8112	–2.0212
**4**	30.0	110.0	4.0	10.0	66.08	68.3726	–2.2926	77.56	79.3012	–1.7412
**5**	70,0	110.0	4.0	10.0	82.35	80.1634	2.1866	89.90	89.3068	0.5932
**6**	30.0	50.0	8.0	10.0	45.88	45.3559	0.5241	50.24	52.0751	–1.8351
**7**	70.0	50.0	8.0	10.0	48.02	46.7217	1.2983	65.09	65.0106	0.0794
**8**	30.0	110.0	8.0	10.0	68.83	68.1567	0.6733	68.37	67.8856	0.4844
**9**	70.0	110.0	8.0	10.0	71.46	72.4126	–0.9526	73.27	71.9512	1.3188
**10**	50.0	80.0	6.0	15.0	80.06	79.6809	0.3791	83.82	81.3936	2.4264
**11**	30.0	50.0	4.0	20.0	41.55	42.7026	–1.1526	52.87	55.859	–2.989
**12**	70.0	50.0	4.0	20.0	54.09	52.5734	1.5166	65.62	63.9045	1.7155
**13**	30.0	110.0	4.0	20.0	52.41	51.5184	0.8916	69.81	67.6895	2.1205
**14**	70.0	110.0	4.0	20.0	61.65	64.2792	–2.6292	67.03	66.8651	0.1649
**15**	30.0	50.0	8.0	20.0	45.49	45.4867	0.0033	58.51	56.9034	1.6066
**16**	70.0	50.0	8.0	20.0	48.01	47.8226	0.1874	59.08	59.009	0.071
**17**	30.0	110.0	8.0	20.0	57.76	56.6426	1.1174	63.53	63.179	0.351
**18**	70.0	110.0	8.0	20.0	62.61	61.8684	0.7416	56.09	56.4145	–0.3245
**19**	50.0	80.0	6.0	15.0	80.04	79.6809	0.3591	82.89	81.3936	1.4964
**20**	30.0	80.0	6.0	15.0	75.33	76.5428	–1.2128	77.99	78.5115	–0.5215
**21**	70.0	80.0	6.0	15.0	84.48	83.6061	0.8739	82.97	84.5671	–1.5971
**22**	50.0	50.0	6.0	15.0	51.32	51.9228	–0.6028	79.47	78.6215	0.8485
**23**	50.0	110.0	6.0	15.0	69.44	69.1761	0.2639	85.04	88.0071	–2.9671
**24**	50.0	80.0	4.0	15.0	79.77	76.5161	3.2539	71.56	71.9271	–0.3671
**25**	50.0	80.0	8.0	15.0	70.44	74.0328	–3.5928	62.02	63.7715	–1.7515
**26**	50.0	80.0	6.0	10.0	84.22	83.8828	0.3372	82.11	81.5126	0.5974
**27**	50.0	80.0	6.0	20.0	75.33	76.0061	–0.6761	69.99	72.7059	–2.7159
**28**	50.0	80.0	6.0	15.0	80.22	79.6809	0.5391	82.02	81.3936	0.6264

#### 3.5.2 2D and 3D Surface Plot of the Degradation (R) of IC and MB

A mathematical model based on the central composite design was employed to analyze the combined impact of composite mass (A), time (B), pH (C) and concentration (D) on the degradation of IC and MB. Figures 6a1–c2 and S52D and Figures 6d1–f2 and S63D show the effects of factors A, B, C and D on the degradation of IC and MB. Figure 6a1, a2, d1 and d2 show the combined effect of factors A and D, demonstrating that high composite masses of ZrO_2_@WO_3_-25/60 hybrid oxides in the photocatalysts improve the degradation efficiency of IC and MB. In contrast, high concentrations of IC and MB ranging from 15 to 20 ppm significantly affect degradation efficiency. This high efficiency is attributed to the high concentration of oxygen vacancies in the ZrO_2_@WO_3_-25/60 hybrid oxides. These vacancies are responsible for light absorption and the transfer of electrons from the valence band to the conduction band.^52^ This leads to the rapid recombination of photo-generated electron-hole pairs^54^ in the conduction and valence energy bands. These bands are responsible for oxidation and reduction reactions.^50^

**Figure 6:**
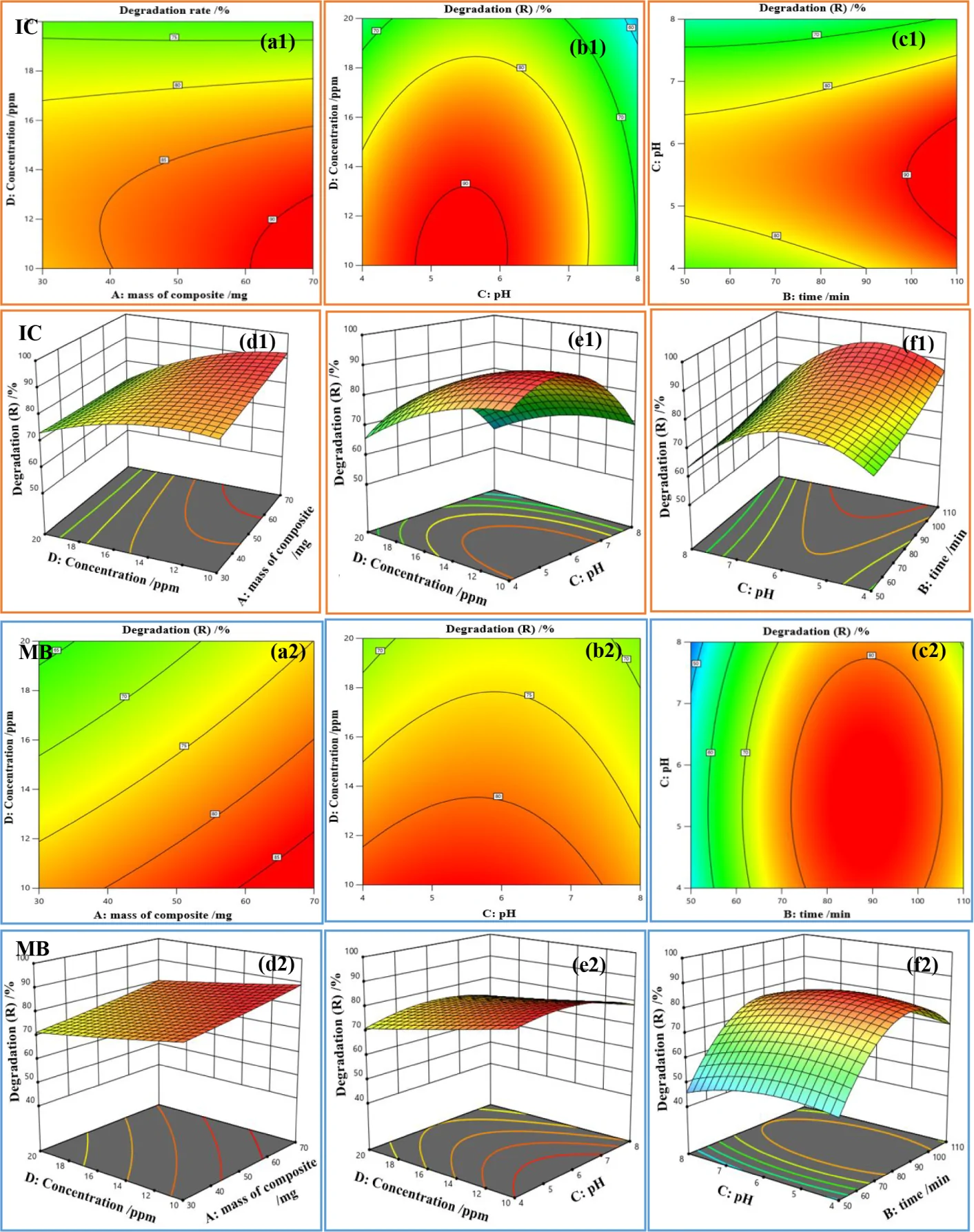
2D and 3D surface plot of the degradation (%) against effects of mass of composite vs. concentration a (1–2) and d (1–2), pH vs. concentration b (1–2) and e (1–2), and time vs. pH c (1–2) and f (1–2) for IC and MB, respectively.

Figure 6b1, b2, e1 and e2 indicate that the combined effect of factors C and D reduces the photocatalytic activity of ZrO_2_@WO_3_-25/60 hybrid oxides, whereas mass tends to increase degradation efficiency. The impact of pH on photocatalytic activity was most significant at moderate pH levels (5.9–6.4), with an optimal pH value. At pH values above 7.5, the IC and MB molecules exist in an anionic form due to the high concentration of hydroxide ions in the solution. Similarly, at pH values below 4.8, the IC and MB molecules exist in cationic form due to the high concentration of hydrogen ions in the solution. Consequently, at pH > 7.5, the negative charges created on the surface of the ZrO_2_@WO_3_-25/60 hybrid oxides and the IC^–^ /MB^–^ and OH^–^ forms tend to repel each other. Similar behaviors are observed at low (acidic) pH values due to repulsion between IC^+^ /MB^+^ and the photocatalysts’ positive surface. Figures 6c1, c2, f1 and f2 show that the combined effect of factors B and C enhances the photocatalytic activity of ZrO_2_@WO_3_-25/60 hybrid oxides. However, pH tends to reduce the degradation rate. Prolonged reaction times allow for efficient absorption of visible light during irradiation of IC, MB, and material molecules due to increased adsorption kinetics and large amounts of energy supplied to the system during adsorption.^53^ These observations imply that ZrO_2_@WO_3_-25/60 hybrid oxides are primarily driven by oxidation/reduction reactions responsible for photocatalytic activity via recombination of photo-generated electrons. The observed degradation efficiency is due to the ability of ZrO_2_@WO_3_-25/60 hybrid oxides to absorb visible light photons and separate charges thanks to their narrow bandgap energy, which produces the necessary charge carriers. These carriers have electrons with high micro-reactivity in the conduction band, thereby prolonging the lifetime of the excited charge carriers.^54^ The simultaneous presence of these factors enhances charge carrier interactions, photon absorption at the oxide surface, and redox reactions with the IC and MB molecules. This enhances the efficient degradation of IC and MB.^55^

#### 3.5.3 Photocatalytic Activity

The photocatalytic activity of ZrO_2_, the ZrO_2_@WO_3_-25/60 hybrid oxide, and a visible light source (photolysis) was evaluated with respect to the degradation of IC and MB under irradiation from an artificial visible light source and without light irradiation. The evaluation was conducted using a composite mass of 40 mg, an exposure time of 110 min, a pH of 6.5, and a concentration of 15 ppm. The results obtained are presented in Figure 7a and 7b for IC and MB, respectively. Figure 7a and 7b demonstrate that visible light irradiation alone is insufficient for the degradation of IC and MB. Consequently, degradation yields of 0.21% and 0.18% were obtained for IC and MB, respectively, after 110 min (see Figure 7b and 7d). ZrO_2_ combined with the visible light source, however, exhibited low photocatalytic activity in the degradation of the dyes; yields of 40% and 47% were obtained for IC and MB, respectively, after 110 min of visible light irradiation (see Figure 7b and 7d). Nevertheless, the combined effect of ZrO_2_ and WO_3_ significantly improves photocatalytic activity for dye degradation. The ZrO_2_@WO_3_-25 hybrid oxide was more effective at degrading the MB dye than the IC dye, achieving degradation efficiencies of 73% and 82%, respectively, after 110 min of exposure to visible light. In contrast, the ZrO_2_@WO_3_-60 hybrid oxide proved more effective at degrading the IC dye than the MB dye. Degradation efficiencies of 84% and 78% were achieved for IC and MB, respectively, after 110 min of visible light irradiation prior to the application of the mathematical model.

**Figure 7:**
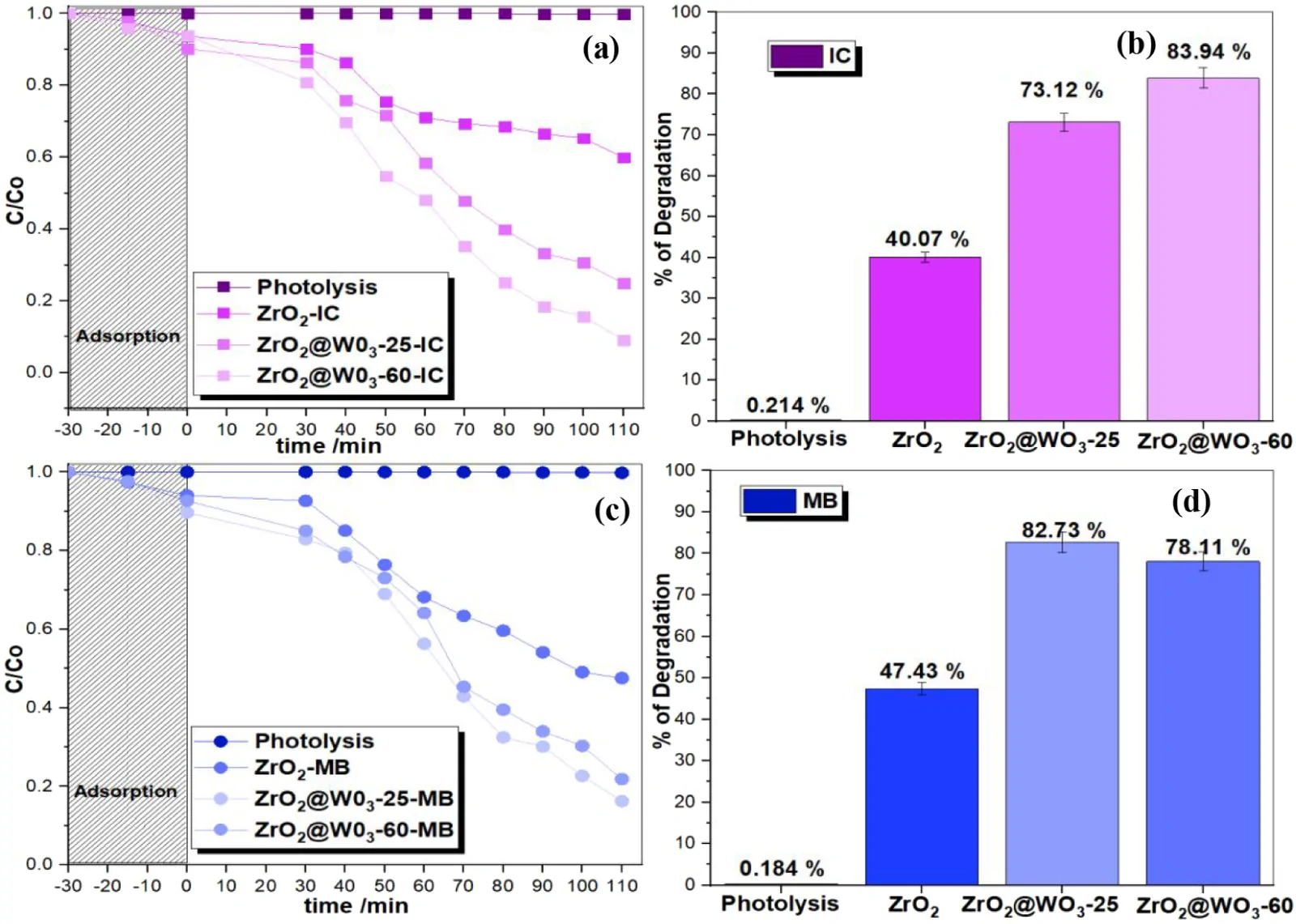
Photocatalytic activity for IC (a) and MB­(c), degradation rate for IC (b) and MB (d).

To demonstrate the effectiveness of hybrid oxides in degrading IC and MB, control experiments were carried out under the same conditions as the photocatalytic tests, but without light irradiation. The results obtained are presented in Figure S7d–g. Clearly, without exposure to light, the hybrid oxides exhibit very low degradation activity toward the two pollutants studied. Indeed, the observed degradation percentages remained below 20% for all materials tested. This slight decrease in dye concentration cannot be attributed to a photocatalytic process since the absence of photons prevents the formation of electron-hole pairs, which are necessary for generating the reactive species responsible for oxidizing organic molecules.^56,57^ Thus, the reduction observed is mainly due to the adsorption of dye molecules onto the surface of the hybrid oxides. Electrostatic interactions, surface bonds and the porosity of the materials promote the binding of IC and MB molecules, leading to an apparent decrease in their concentration in the solution. These results therefore confirm that the effective removal of pollutants observed under light irradiation is primarily due to the photocatalytic activity of the hybrid oxides rather than a simple adsorption phenomenon. Furthermore, the low degradation observed in the dark highlights the crucial role of light in the activation of the materials and in the production of the oxidizing species involved in the degradation of organic pollutants. The performance of the hybrid oxides is presented in the UV-visible curves (see Figure S7 from a-1 to c-2). Figure S7 shows that ZrO_2_@WO_3_-25/60 exhibits the best dye degradation performance, comparable to visible light (photolysis) and ZrO_2_. The low degradation rates observed with ZrO_2_ and the visible light source could be due to ZrO_2_’s inadequate absorption of visible light, as evidenced by the rapid recombination of charge carriers in PL analyses,^58,59^ as well as the absence of oxygen vacancies responsible for absorbing photons of photo-generated energy produced by the light source.^50^

To evaluate the performance of ZrO_2_@WO_3_-25/60 hybrid oxides effectively, a mathematical approach was adopted to investigate the factors likely to improve the photocatalysts’ performance simultaneously. The effects of pH, composite mass, reaction time and dye concentration were studied. UV-visible spectra before and after optimization (see Figure 8a and c, then b and d) clearly demonstrate that the combined impact of these factors significantly enhances the photocatalysts’ performance (ZrO_2_@WO_3_-25/60 hybrid oxides) during IC and MB degradation. The degradation yields of approximately 95% and 96% are comparable to the yields of 82% and 84% for MB and IC, respectively, on the ZrO_2_@WO_3_-25 and ZrO_2_@WO_3_-60 photocatalysts after 110 min of visible light irradiation with a compound mass of 59.9 mg, a pH of approximately 6.06 and a concentration of 10 ppm. For MB, an optimal degradation rate of 92.02% was achieved with a compound mass of 65.1 mg, a reaction time of 109 min, a pH of 5.96 and a dye concentration of 10 ppm. The optimal degradation performance indicates efficient photon absorption at the surface of the materials and dyes due to the high concentration of oxygen vacancies in the hybrid oxides.^52^ This leads to rapid electron recombination in the conduction bands that are responsible for redox reactions.^50,54^ The effect of pH was most favorable at a moderate pH of 5.9–6.4, with an optimal pH. At a pH of less than 4.8, the IC and MB molecules are in cationic form due to the acidic pH. At a pH greater than 7.5, they are in anionic form due to the high pH of the solution. Consequently, at pH > 7.5, the negative charges created on the surface of the ZrO_2_@WO_3_-25/60 hybrid oxides and the IC and MB molecules create electrostatic repulsion between the anionic IC^–^ /MB^–^ and OH^–^ forms. Effective absorption of visible light during irradiation of the surface of the dyes and the material is improved by a sufficient amount of oxide mass, an appropriate reaction medium pH, low concentrations and long reaction times, due to the large amounts of energy supplied to the system during adsorption and the increased adsorption kinetics.^53^

**Figure 8:**
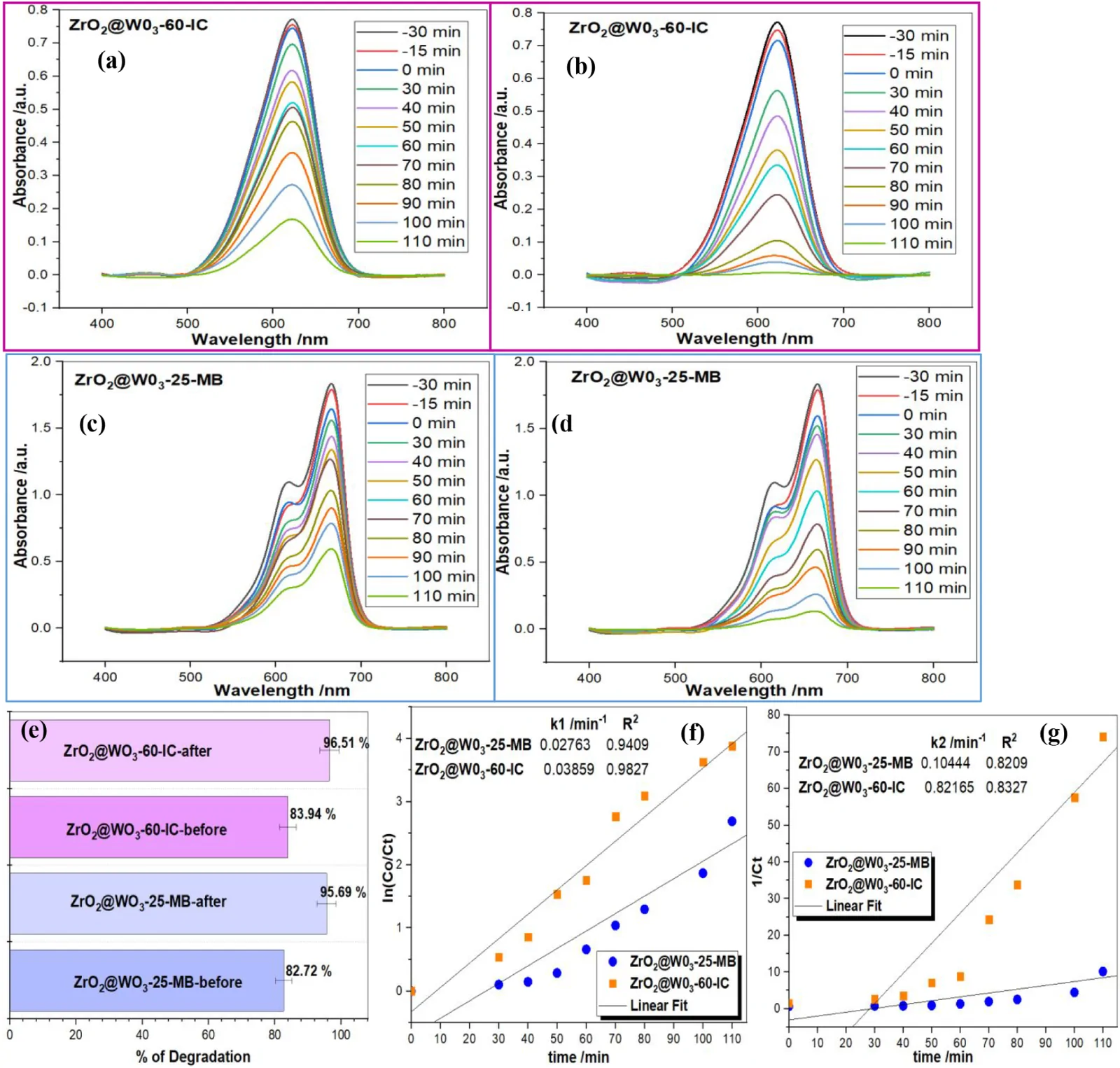
UV-visible curves before (a and c) and after (b and d) optimization for IC and MB, respectively; degradation rate (e); pseudo-first-order (f) and second-order (g) plots.

Pseudo-first-order and second-order kinetic models were employed to analyze the degradation kinetics. The rate constants (*k*_1_ and *k*_2_) for photodegradation were determined for the pseudo-first-order and second-order models (see Figure 8f and g). The rate constants for the pseudo-first-order model were obtained for the MB and IC dyes: ZrO_2_@WO_3_-25-MB (*k*_1_ = 0.027633 min^–1^) and ZrO_2_@WO_3_-60-IC (*k*_1_ = 0.03858 min^–1^). For the second-order model, the rate constants for the MB and IC dyes were found to be 0.010444 min^–1^ and 0.82165 min^–1^, respectively. Of the two kinetic models studied, the pseudo-first-order model provides a more accurate description of the degradation kinetics of MB and IC. This is evident from the significantly higher values of the rate constant *k*_1_ obtained using the pseudo-first-order model compared to those obtained using the pseudo-second-order model. These high rate constants indicate that the kinetic model is effective for both the dye and the hybrid oxide.^13^ The observed degradation efficiency is therefore likely due to the hybrid oxides’ ability to absorb visible light photons, their high charge separation and their enhanced redox reactions, which are all effective in degrading IC and MB.^55^

#### 3.5.4 Mineralization and Real Water Analysis

The factors that may influence the photocatalytic activity of the ZrO_2_@WO_3_-25 and ZrO_2_@WO_3_-60 catalysts were evaluated. The impact of anions (Cl^–^, NO_3_^–^, CO_3_^2–^, and SO_4_^2–^) was also assessed. Figure 9a (1–2) show the results obtained for IC and MB. Cl^–^ and SO_4_^2 –^ ions were found to have little to no influence on photocatalytic performance, while NO_3_^–^ and CO_3_^2 –^ ions were found to have a significant impact on the photodegradation of IC and MB. Degradation efficiencies of 81%, 84%, 64%, and 62% were observed for IC with the ZrO_2_@WO_3_-25 catalyst; degradation efficiencies of 83%, 82%, 68%, and 66% were observed for MB with the ZrO_2_@WO_3_-60 catalyst, respectively, for the anions Cl^–^, SO_4_^2 –^, CO_3_^2 –^, and NO_3_^–^. The low degradation efficiency of IC and MB in the presence of NO_3_^–^ and CO_3_^2 –^ anions may be related to their reducing capacity, which consumes electrons necessary for photodegradation.^9^ The impact of cations (Na^+^, K^+^, Mg^2+^, and Ca^2+^) on the photocatalytic activity of ZrO_2_@WO_3_-25 and ZrO_2_@WO_3_-60 catalysts was evaluated. Figure 9b (1–2) show the results. Na^+^ and Mg^2+^ ions were found to have a weak influence on the photocatalytic performance of the catalysts, comparable to K^+^ and Ca^2+^ ions. The latter exhibited a more noticeable influence during the photodegradation of IC and MB. The degradation efficiency for ZrO_2_@WO_3_-60 was 87%, 85%, 80%, and 69% for the cations Ca^2+^, Mg^2+^, Na^+^, and K^+^, respectively. For the ZrO_2_@WO_3_-25 catalyst, the degradation efficiencies were 85%, 85%, 78%, and 73% for the cations Ca^2+^, Mg^2+^, Na^+^, and K^+^, respectively.^9^ The influence of the K^+^ cation can be explained by its large ionic radius, which could block active sites necessary for light absorption during irradiation.

**Figure 9:**
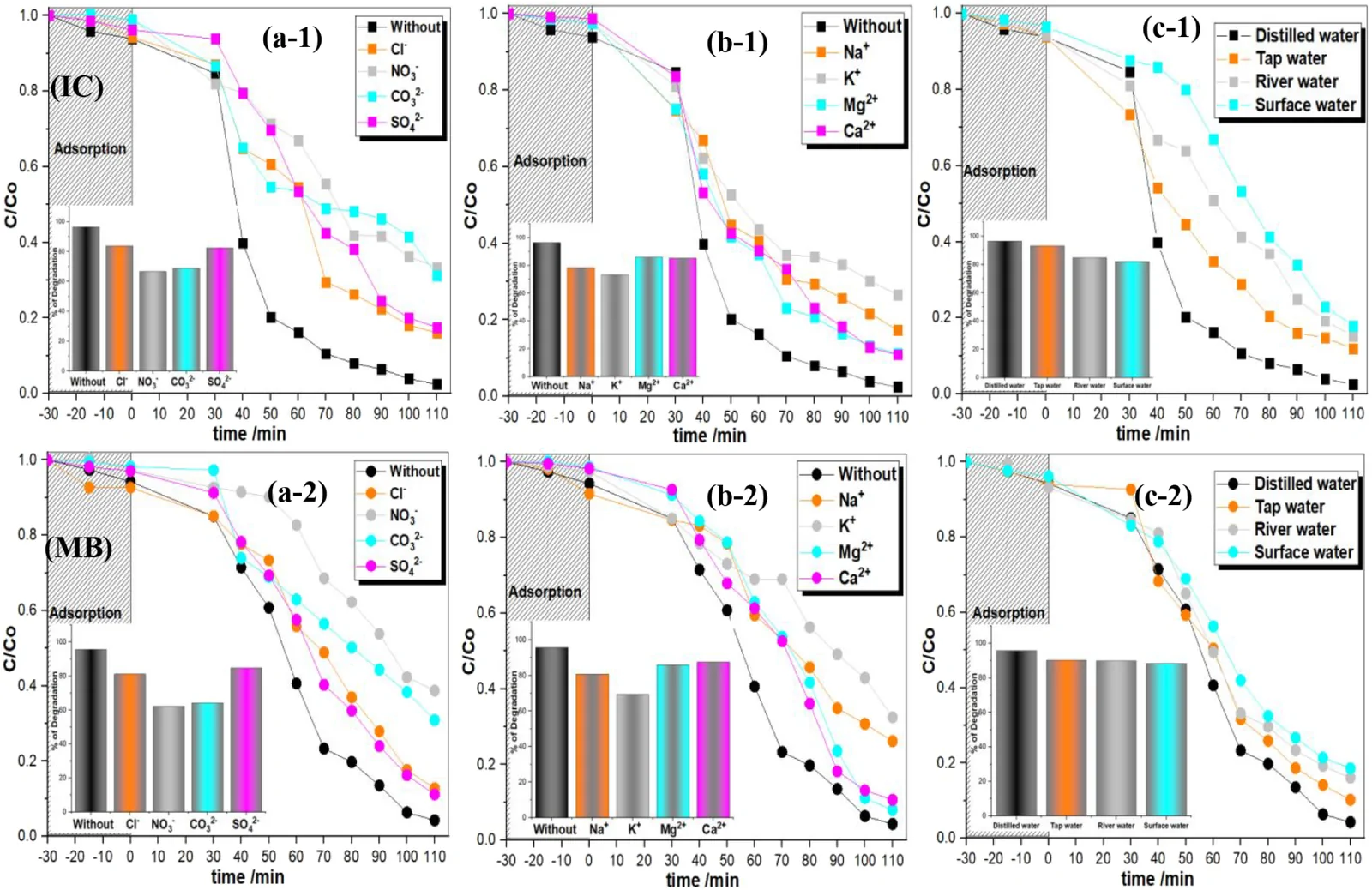
Impact of anions (Cl^–^, CO_3_^2 –^, NO_3_^–^, and SO_4_^2 –^) (a-1 and a-2), cations (Na^+^, K^+^, Mg^2+^, and Ca^2+^) (b-1 and b-2), and the water source (c-1 and c-2) on IC and MB, respectively.

The effect of the water source was evaluated to ensure the simultaneous presence of anions and cations. Figure 9c (1–2) show the obtained results. Surface water was found to significantly influence the photocatalytic activity of the ZrO_2_@WO_3_-25 and ZrO_2_@WO_3_-60 catalysts during the photodegradation of the IC and MB dyes, followed by river water, tap water, and distilled water. For ZrO_2_@WO_3_-60, the degradation efficiencies were 93%, 84%, and 82% during photodegradation of the IC dye. ZrO_2_@WO_3_-25 exhibited degradation efficiencies of 90%, 89%, and 88% during the photodegradation of MB for tap water, river water, and surface water, respectively. These efficiencies are comparable to those of distilled water, which achieved 96.51% for ZrO_2_@WO_3_-60 on the IC dye and 95.69% for ZrO_2_@WO_3_-25 on the MB dye. The strong influence of river and surface water is supported by the presence of anions and cations previously studied in both effluents,^60^ comparable to distilled and tap water, which have fewer anions and cations. The decrease in the degradation rate of ZrO_2_@WO_3_-25 and ZrO_2_@WO_3_-60 catalysts in the presence of ionic species is due to their inability to generate sufficient radicals after absorbing light necessary for decomposing the dye molecules in solution.

### 3.6 DFT and the Degradation Mechanism of IC and MB Dyes

#### 3.6.1 DFT Analysis

The active catalytic sites of the IC and MB dyes were evaluated through theoretical simulations. The optimized structure of the dyes was obtained using the Forcite module. The LUMO, HOMO and energy gap (ΔE) were then calculated using the DMoL3 module and the GGA/PW91 functional, which are both integrated into the Materials Studio software. The Fukui indices for electrophilic (*f* ^–^), radical (*f* ^0^) and nucleophilic (*f* ^*+*^) attacks were determined, along with the corresponding Hirshfeld charge distribution and frontier electron density for the IC and MB dyes (see Tables S1–S2) was used to identify sites vulnerable to attack by superoxide radicals (•O_2_^–^), holes (h^+^) and hydroxyl radicals (•OH).^61^ Figures 10a­(1–2), 10b­(1–2), 10c­(1–2) and 10d­(1–2) show the optimized 3D structures, electron density, LUMO energies, HOMO energies and energy gap (ΔE) for the IC and MB dyes, respectively. The IC and MB dyes exhibit ΔE values of 1.3 and 1.2 eV, respectively. However, the IC dye exhibits a distribution of the LUMO across the benzene and pyrrole rings, while the HOMO is oriented toward the pyrrole ring. MB, on the other hand, exhibits a distribution of the LUMO at the ends of the *N*-methyl groups and a symmetrically distributed HOMO. Analysis of Fukui indices for radical, electrophilic and nucleophilic attacks revealed the dyes’ most vulnerable sites during radical attack,.^12,33^ The atoms C18, O24, S21, S22, O25, O26, O27, O28, O29, and O30 of the IC dyes (Figure S8), as well as the atoms C6, S10, C17, C18, C19, and C20 of the MB dyes (Figure S9), have the highest *f* ^0^, *f* ^–^, and *f* ^*+*^ values. This suggests that these atoms are most vulnerable to attack by •O_2_^–^, h^+^ and photogenerated free •OH radicals by the ZrO_2_@WO_3_-25 and ZrO_2_@WO_3_-60 catalysts.^62^

**Figure 10:**
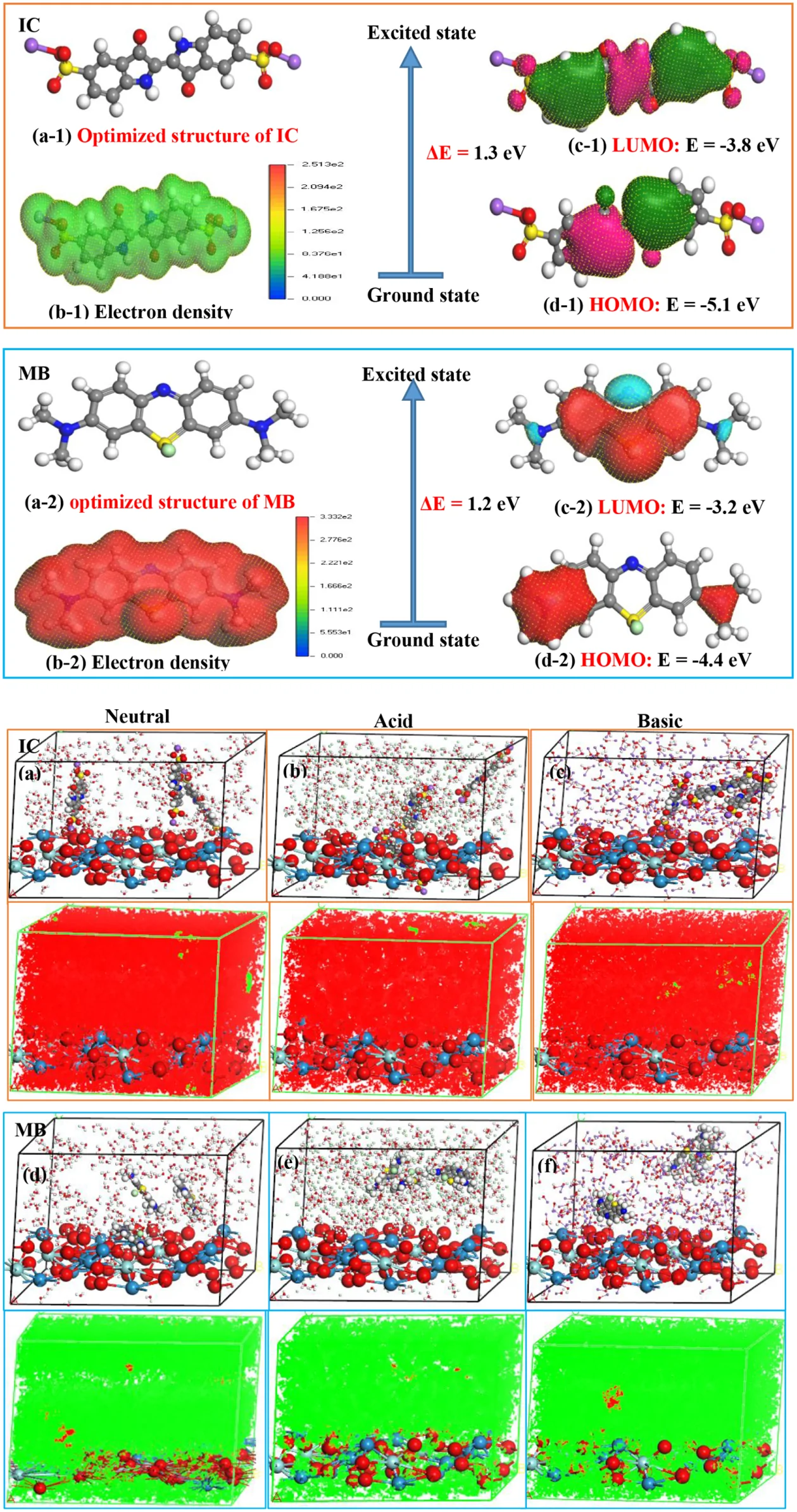
Optimized 3D structures a(1–2), electron density b(1–2), LUMO c(1–2), and HOMO d(1–2) for IC and MB dyes, respectively. Reduced 3D structure of ZrO_2_@WO_3_ loaded with three molecules of IC or MB in neutral (a and d), acidic (b and e), and basic (c and f) medium.

In order to thoroughly investigate the adsorption mechanism of IC and MB dyes on the ZrO_2_@WO_3_ photocatalyst, the photocatalyst’s structure was split and reconstructed within a cube with dimensions of a = 45 Å, b = 49 Å and c = 34 Å (see Figure S10). The X-ray diffraction patterns of ZrO_2_ and WO_3_, which were used for the DFT calculations, are presented in Figure S11. The reconstructed structure was then loaded with three molecules of IC and MB dyes, and the field profile was tracked following the addition of 300 H_2_O Figure 10a and d, 300 (H_3_O^+^ + Cl^–^) Figure 10b and e, and 300 (Na^+^ + OH^–^) Figure 10c and f, which correspond to neutral, acidic, and basic environments, respectively. As can be seen in the figures, the IC and MB molecules are oriented laterally toward the photocatalyst surface, promoting proper adsorption and enabling efficient absorption of visible light when the dye and material surfaces are irradiated. This is due to the large amounts of energy supplied to the system during adsorption, which accelerates the adsorption kinetics. The ZrO_2_@WO_3_ photocatalyst exhibits the highest adsorption energy in a neutral medium. This is corroborated by the highest distribution probability (P­(E)) for the IC and MB energies at approximately 0.2 (see Figure S12), which is a value comparable to that observed in acidic and basic media. These observations are further supported by the values of the dynamic descriptors related to the adsorption of IC and MB in various media on ZrO_2_@WO_3_ (see Table S3). This table shows that the individual adsorption energy (dEad/dNi) is highest in a neutral medium and then in a basic medium. These results are consistent with the pH effect discussed in Section 3.5.1. Indeed, moderate values of pH (5.9–6.4) were most favorable; at acidic pH values, the dye molecules exist in cationic form, whereas at basic pH values, they exist in anionic form. Consequently, at acidic pH, the positive charges created on the surface of the ZrO_2_@WO_3_-25/60 hybrid oxides and on the IC and MB molecules generate electrostatic repulsion. The simultaneous presence of these factors enhances the overall interactions between charge carriers and the absorption of photons by the oxides, IC and MB molecules, as well as promoting redox reactions. This leads to the effective degradation of these molecules.

#### 3.6.2 Scavengers Analysis

The influence of radical scavengers on the photodegradation of IC and MB was investigated using the following: p-benzoquinone (p-BQ) for •O_2_^–^; disodium ethylenediaminetetraacetate (EDTA-2Na) for h^+^; *tert*-butyl alcohol (*t*-BuOH) for •OH; and hydrogen peroxide (HP) for e.^63^ Figures 11a (1-11) show the results. It can be seen that p-benzoquinone significantly influences photocatalytic activity. Degradation efficiencies of 18% and 14% were obtained for ZrO_2_@WO_3_-60 and ZrO_2_@WO_3_-25 for MB and IC dyes, respectively. In contrast, the addition of *t*-BuOH resulted in a slight decrease in degradation efficiency, with values of 82% and 84% for ZrO_2_@WO_3_-25 and ZrO_2_@WO_3_-60, respectively, for the MB and IC dyes. Adding EDTA-2Na resulted in degradation efficiencies of 26% for ZrO_2_@WO_3_-25 and 29% for ZrO_2_@WO_3_-60. Adding HP increased the degradation efficiencies to 99% for ZrO_2_@WO_3_-25 on the MB dye and 96% for ZrO_2_@WO_3_-60 on the IC dye. Without radical scavengers, the efficiencies were 96.51% for ZrO_2_@WO_3_-60 on the IC dye and 95.69% for ZrO_2_@WO_3_-25 on the MB dye (see Figure 11b­(1–2)). These observations demonstrate that h^+^, ^•^O_2_^–^ and •OH) are responsible for degrading the IC and MB dyes.^10^ In light of these observations, it can be assumed that ZrO_2_@WO_3_-25/60 hybrid oxides are predominantly photon-driven,^64^ thereby promoting redox reactions through the recombination of photogenerated electrons. The observed degradation efficiency is due to the ability of the ZrO_2_@WO_3_-25/60 hybrid oxides to absorb visible light photons and separate charges. This is thanks to their narrow bandgap energy, which produces the necessary charge carriers and enhances redox reactions for the effective degradation of IC and MB.^55^ Thus, electrons photogenerated by ZrO_2_@WO_3_-60 after visible light absorption lead to the reduction of molecular oxygen, resulting in the formation of superoxide radicals.^54^ The IC and MB dyes are then excited to IC* and MB* under visible light irradiation due to photon absorption. This is followed by electron transfer from IC* or MB* to the ZrO_2_@WO_3_-60 catalyst. This leads to the formation of IC^•+^ or MB^•+^. The decomposition of IC^•+^ or MB^•+^ by superoxide radicals results in the formation of holes (h^+^) or •OH, which convert to H_2_O and CO_2_ according to eqs 8–14.

8 $$IC\;or\;MB+h\upsilon \to IC^{•+}\;or\;MB^{•+}+e^{-}$$

9 $$ZrO_{2}@WO_{3}+h\upsilon \to ZrO_{2}@WO_{3}\left(h^{+}+e^{-}\right)$$

10 ZrO2@WO3(e−)+O2→ZrO2@WO3+O•2−

11 O•2−+H+→HO2•

12 $$ZrO_{2}@WO_{3}\left(e^{-}\right)+H{O_{2}}^{•}+H^{+}\to ZrO_{2}@WO_{3}{(}^{•})+H_{2}O_{2}$$

13 $$ZrO_{2}@WO_{3}\left(e^{-}\right)+H_{2}O_{2}\to OH^{•}+OH^{-}$$

14 $$IC^{•}+or\;MB^{•+}+OH^{•}{/}^{•}O2^{-}\mathrm{degradation}\;\mathrm{products}$$

**Figure 11:**
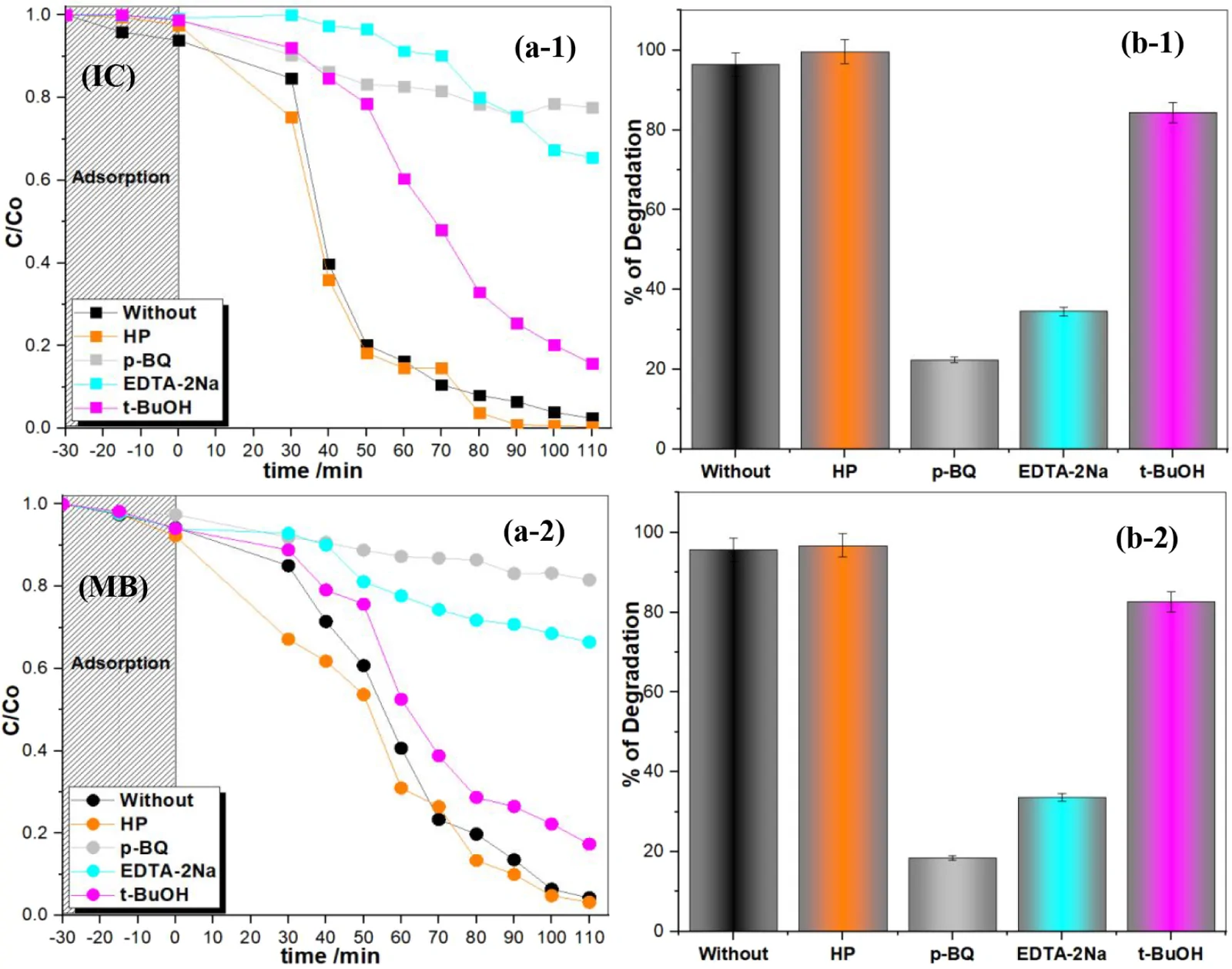
Scavengers (a-1 and a-2), degradation rate (b-1 and b-2), for IC and MB dyes, respectively.

#### 3.6.3 Degradation Pathway and Intermediates of IC and MB

Analysis of the LC-MS degradation spectra of IC and MB (Figure S13) indicates that ZrO_2_@WO_3_-25/60 metal hybrid oxides become effective after 110 min of oxidation. Photodegradation proved effective in completely mineralizing both dyes, as evidenced by the decrease in the m/z signals of IC (m/z = 423) and MB (m/z = 284) and the formation of a greater number of intermediate compounds. This suggests that the energetic action of the oxides absorbed light photons and that charge separation is high due to the narrow bandgap energy of the hybrid oxides. This allows for the production of charge carriers, such as holes, superoxide radicals and hydroxyl radicals, which are necessary for degrading the IC and MB dyes. Analysis of the LC-MS spectrum of IC shows a single IC anion named P0 (m/z = 423) and intermediates generated by degradation.^65^ IC degradation (see Figure 12a and b) begins with cleavage of the double bond in the pyrrole rings, followed by oxidation of the carbonyl groups to form intermediate P1 (m/z = 459) due to attack by hydroxyl radicals. P1 then undergoes double dehydroxylation to form P2 (m/z = 429), which is converted to P3 (m/z = 413) by further dehydroxylation.^66^ P4 (m/z = 257) is formed by cleavage of the C–C bond in the pyrrole ring,^33^ followed by hydroxylation and oxidation of the −OH groups to form carbonyl groups. The intermediates P6 (m/z = 226) and P5 (m/z = 241) are formed by the successive dehydroxylation of P4. This process disrupts the chromophore groups that are responsible for the blue color of the IC dye, as demonstrated by UV-Vis spectroscopy.^67^ Meanwhile, intermediate P2 is converted to P7 (m/z = 284) through the elimination of bisulfite groups (HSO_3_^–^), which occurs when the S–C bond is cleaved. This is followed by hydroxylation via attack by hydroxyl radicals and the formation of a carbon radical.^68^ Attack by •OH radicals on the carbons of the pyrrole ring leads to cleavage of the C–C bond in P7 and formation of a new C–OH bond. This contributes to formation of intermediate P8 (m/z = 151) and the C_8_H_8_NO_2_• radical.^69^ P8 is then oxidized to P9 (m/z = 149) via the formation of a carbonyl group. Attack of the carbonyl group by •OH leads to cleavage of the N–C bond (see Figure 12a) and formation of the −NH_2_ group by hydrogenation. This results in formation of P10 (m/z = 167). P11 (m/z = 123) is then formed by the decarboxylation (−CO_2_) and hydrogenation of P10 in the presence of H_2_SO_4_ in solution. P11 is then reduced to P12 (m/z = 112) and subsequently to P13 (m/z = 109). The benzene rings then cleave and small molecules (CO_2_, H_2_O, SO_4_^2 –^, NH_4_^+^ and NO_3_^–^ are formed.

**Figure 12:**
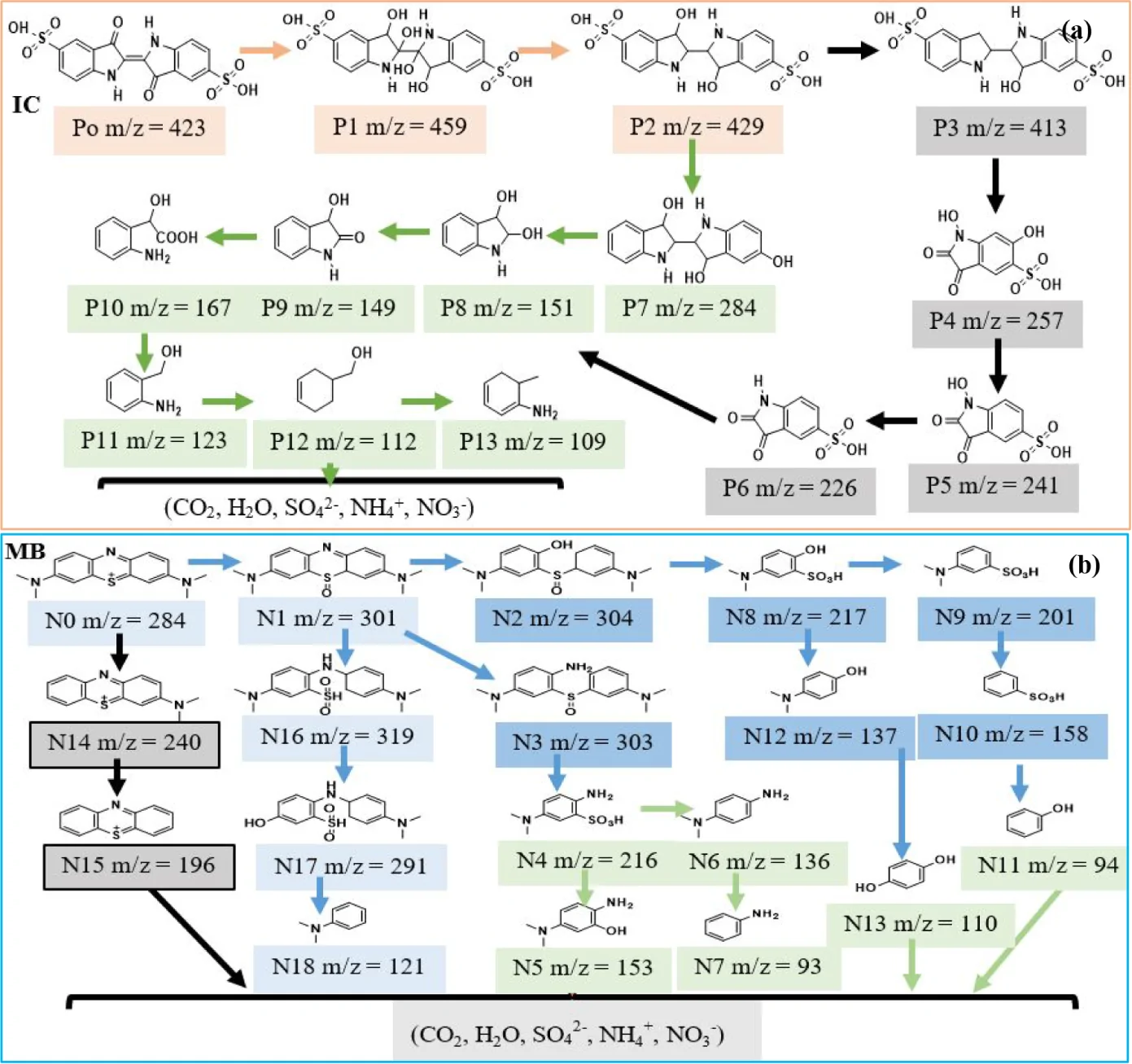
Degradation pathways for IC (a) and MB (b).

Analysis of the LC-MS spectrum of MB shows that degradation can occur through two pathways: MB to N0 (m/z = 284) (see Figure 12b). In pathway I (shown in blue), N1 (m/z = 301) is formed when superoxide radicals (•O_2_^–^) attack N0. Next, N1 is converted to N2 (m/z = 304) through attack by hydroxyl radicals (•OH). Cleavage of the N–C bond in the plane-symmetric ring, followed by hydrogenation, converts N1 to N3 (m/z = 303).^68^ Attack by superoxide radicals and hydroxyl radicals on N3 leads to the formation of N4 (m/z = 216). Then, N4 is reduced to N6 (m/z = 136), N5 (m/z = 153) and N7 (m/z = 93) via elimination of bisulfite groups, demethylation of *N*-methyl groups, and hydroxylation. In another pathway, attack by •OH on N2 results in cleavage of the S–C bond and formation of N8 (m/z = 216). N8 is then reduced to N9, N10, N11, N12 and N13 via dehydroxylation, demethylation of the *N*-methyl groups, elimination of the sulfite groups, and subsequent hydroxylation. Meanwhile, cleavage of the S–C bond at P1 by an attack from •OH leads to the formation of N16 (m/z = 319), which is subsequently reduced to N17 (m/z = 291) through demethylation and hydroxylation of the *N*-methyl groups. Cleavage of the C–N bond reduces N17 to N18 (m/z = 121) with formation of intermediate radicals. In pathway II (shown in black), N0 (m/z = 284) is first converted to N14 (m/z = 240) and then to N15 (m/z = 196) by successive demethylation.^69^ Subsequently, N15, N18, N14, N7, N11 and N13 are converted into small molecules (CO_2_, H_2_O, SO_4_^2–^, NH_4_^+^, NO_3_^–^) by decarboxylation, hydrogenation and cleavage of the benzene rings.

The toxicity of IC, MB and their intermediates was investigated using the Toxicity Estimation Software Tool (T.E.S.T.), in accordance with the consensus method.^33,34^ The following were assessed: fathead minnow LC50 (96 h), oral rat LD50 (mg/kg), Daphnia magna LC50 (48 h), bioaccumulation factor, mutagenicity and developmental toxicity.^70^ Figure 13a (1–2) shows that the IC compound and its by-products (P1, P2, P3, P4, P5 and P7), as well as the MB compound and its by-products (N14, N15, N16 and N17), are highly toxic due to their polycyclic structure. The IC by-products P13, P12 and P10, and the MB by-products N2, N3, N13, N11, N10, N12, N18 and N7, are toxic. However, intermediates P4, P8, P9, P10 and P11, as well as N18, N17, N15 and N14, were classified as harmful. These observations are supported by analyses of the oral LD_50_ in rats Figure 13b (1–13) and Daphnia magna Figure 13c (1–13). Analysis of the bioaccumulation factor Figure 13d (1–13) shows that all intermediates exhibit accumulation factors that increase the concentration of slightly toxic or nontoxic substances. This indicates that the compound under study is being degraded. Similarly, mutagenicity analyses (Figure 13e, 1–2) showed that all intermediates exhibited low mutagenicity and were considered negative, with the exception of P9 and P10 for IC and N14, N15, N16, N17 and N18 for MB, which exhibited high mutagenicity and were considered positive.^11^ Developmental toxicity analyses showed that intermediates P8, P9, P10 and P11 for IC and N17 and N8 for MB were nontoxic Figure 13f (1–2). All other intermediates were toxic, likely due to aromatic rings in their structures. These observations demonstrate that photodegradation of IC and MB results in the formation of intermediates that are either toxic or nontoxic due to oxidation, ring-opening, and cleavage of −OH, -NH_2_, −CH_3_, and −CO groups. This reduces the acute toxicity of IC and MB compounds and their derived intermediates without posing a future risk to organisms and ecosystems.

**Figure 13:**
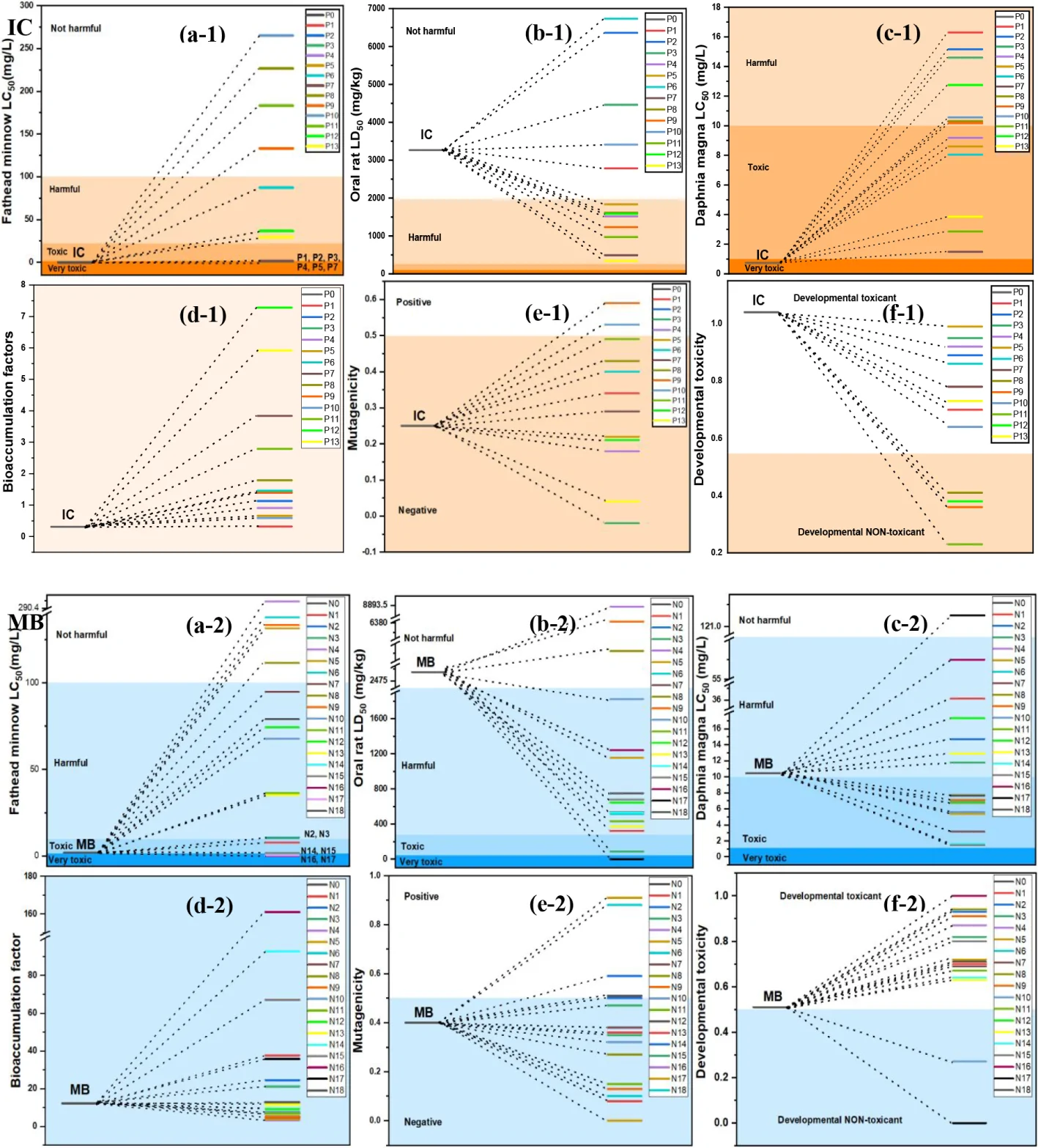
Fathead minnow LC_50_ (96 h) a(1–2), oral rat LD_50_ mg/kg b(1–2), Daphnia magna LC_50_ (48 h) c(1–2), bioaccumulation factor d(1–2), mutagenicity e(1–2), and developmental toxicity f(1–2).

### 3.7 Recyclability, Comparison with the Literature, and Stability

The reusability of the ZrO_2_@WO_3_-25 and ZrO_2_@WO_3_-60 photocatalysts was evaluated over five cycles. Degradation of IC and MB under visible light irradiation was observed, as shown in Figure 14 a and 14b. The photodegradation of the IC and MB dyes remained stable after five cycles of use. During MB removal, the ZrO_2_@WO_3_-25 photocatalyst exhibited degradation rates of 95.69%, 94.99%, 90.86%, 89.91% and 89.01%. In contrast, the ZrO_2_@WO_3_-60 photocatalyst exhibited degradation rates of 96.51%, 94.35%, 91.09%, 91.07% and 89.28% between the first and fifth cycles. The difference in performance between the first and fifth cycles was approximately 6% and 7%, respectively, demonstrating the photocatalytic performance of the developed hybrid oxides. The presence of WO_3_ in the hybrid oxides plays a significant role in light photon absorption and the efficient transfer of photoinduced electrons. The coexistence of ZrO_2_ oxides and monoclinic and hexagonal WO_3_ phases in ZrO_2_@WO_3_-25 and ZrO_2_@WO_3_-60 induces a structural transformation of the monoclinic ZrO_2_ phase into a more stable tetragonal phase.^33,34^ Consequently, there are more oxygen vacancies responsible for light absorption and the transfer of •O_2_^–^, electrons, and •OH radicals, which are necessary for redox reactions and induce recombination.^54^ Table S4 summarizes the degradation efficiency of dyes using existing oxides. As can be seen from this table, the developed hybrid oxides perform well in the photodegradation of IC and MB dyes. XRD analyses (Figure 14c) of the spent ZrO_2_@WO_3_-25 and ZrO_2_@WO_3_-60 catalysts reveal the same peaks as the initial photocatalysts due to preservation of the tungsten-doped samples’ crystalline structure. SEM analyses (Figure 14e and f) of the spent catalysts reveal WO_3_ particles that can be attributed to the hexagonal and monoclinic WO_3_ phases, which are comparable to those observed in the fresh catalysts. EDX analyses (Figure 14d) of the spent ZrO_2_@WO_3_-60 catalyst show a uniform distribution of W within the ZrO_2_ pores, as well as the presence of the elements C, O, Zr and W. Similar phenomena are observable for ZrO_2_@WO_3_-25 (see Figure S14), confirming that the composition of the samples remained unchanged after the degradation step. The fresh ZrO_2_@WO_3_-25 catalyst exhibited approximately 3% C, 20% O, 58% Zr and 19% W, whereas the spent catalyst showed approximately 4% C, 20% O, 60% Zr and 16% W. The relatively small decrease in W content following photocatalytic treatment, coupled with the almost unchanged O content and slight increase in relative Zr content, suggests that the catalyst retains its overall elemental composition during photocatalysis. This result provides preliminary evidence of good structural stability and limited loss of the tungsten-containing phase.

**Figure 14:**
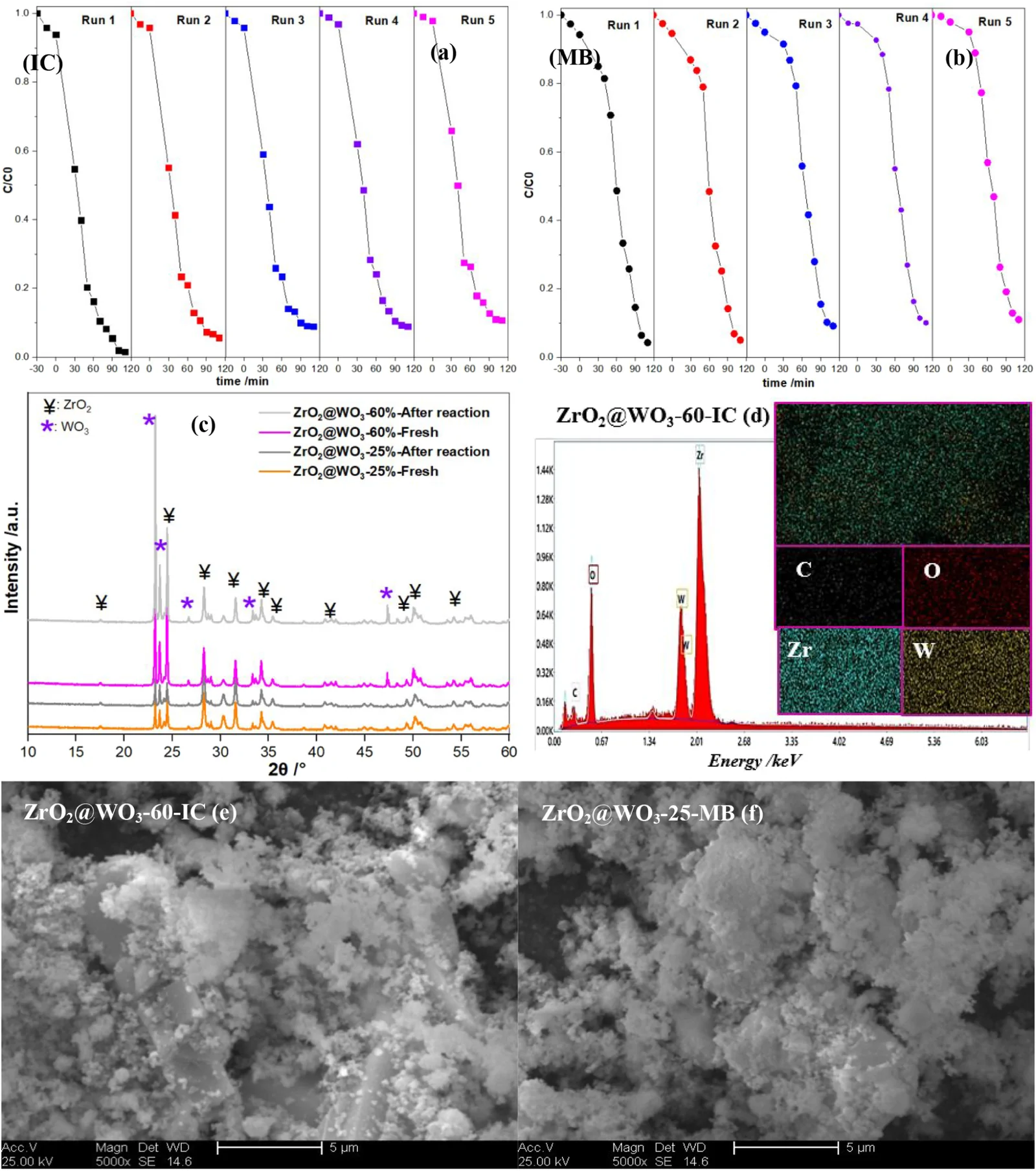
Recyclability (a and b), XRD patterns of ZrO_2_@WO_3_-25 and ZrO_2_@WO_3_-60 (c), EDX mapping of ZrO_2_@WO_3_-60 (d), SEM images of ZrO_2_@WO_3_-60 (e) and ZrO_2_@WO_3_-25 (f) after IC and MB degradation.

## 4 Conclusion

The aim of this work is to develop efficient and stable ZrO_2_@WO_3_-X hybrid photocatalysts for the photodegradation of MB and IC organic dyes, using a mathematical approach that combines hydrothermal-assisted pyrolysis with a (Zr/W)­UiO-66 MOF-type template. Analyses using XRD, TGA, FTIR, XPS, EDX, SEM, PL, UV-vis DRS, N_2_ sorption, EIS and Raman spectroscopy reveal the formation of t-ZrO_2_, m-ZrO_2_ and monoclinic WO_3_ oxides. Raman analysis reveals the presence of phases typical of tetragonal and monoclinic crystalline WO_3_, corresponding to the stretching modes of bridging oxygen atoms. According to mathematical modeling, the degradation yield of IC reached 95.11% and that of MB reached 92.82% under optimal conditions, with an R^2^ of 97.70%. DFT and MC simulation studies show that the LUMO is distributed across the benzene and pyrrole rings, while the HOMO is oriented toward the pyrrole ring for IC. MB exhibits a LUMO distribution at the ends of the *N*-methyl groups, with a symmetrical HOMO distribution across the dye plane. Analysis of Fukui indices for radicals indicates that the *N*-methyl sites are the most susceptible to attack by •O_2_^–^, H^+^ and •OH radicals. Analysis of radical scavengers indicates that H^+^, •O_2_^–^ and •OH species promote dye degradation. LC-MS analysis shows mineralization of the dyes via cleavage of the pyrrole rings, followed by oxidation of the carbonyl groups and formation of intermediates. The presence of WO_3_ in the hybrid oxides plays a significant role in absorbing light photons, transferring them, and subsequently separating photo-induced electrons efficiently. The toxicity of IC and MB were assessed using the fathead minnow LC50 (96 h), oral rat LD50 (mg/kg), Daphnia magna LC50 (48 h), bioaccumulation factor, mutagenicity and developmental toxicity. The materials proved to be stable over five cycles of use. XRD analysis of the spent catalysts shows the same reflections as the initial photocatalysts due to preservation of the tungsten-doped samples’ crystalline structure.

## Supplementary Material



## Notes

Methods, such as EDX elemental analysis and elemental mapping of ZrO_2_ and ZrO_2_@WO_3_-60, as well as TGA/DTG curves of UiO-66 and Zr/W-UiO-66; statistical validation plots for the IC degradation model, including residuals versus predicted values, residuals versus composite mass, Cook’s distance, leverage versus run, and DFBETAS for the intercept versus run; 2D and 3D response surface plots illustrating the effects of composite mass, reaction time, initial dye concentration, and pH on the degradation efficiency of IC and MB; UV-Vis spectra obtained prior to optimization, photocatalytic activity under dark conditions, and degradation efficiencies of IC and MB using ZrO_2_, ZrO_2_@WO_3_-25, and ZrO_2_@WO_3_-60; 3D structures and XRD patterns of ZrO_2_ and WO_3_ used for the DFT calculations; LC–MS spectra of IC and MB; a comparative summary of the degradation efficiencies reported for various photocatalysts (PDF)
