# Supplementary material for: Engineering Zr–W Mixed Oxide Heterojunctions from UiO-66-Type MOFs Templates: Bridging DFT and Mechanistic Insights into Organic Abatement
Source: ACS Omega. 2026 Sep 16;11(38):58306–29. doi: 10.1021/acsomega.6c08746 (PMC13628992; doi:10.1021/acsomega.6c08746)
Supplement: Supplementary file 1 [file ao6c08746_si_001.pdf]

Supporting Information

Engineering Zr–W Mixed Oxide Heterojunctions from UiO-66-Type MOFs Templates: Bridging DFT, Mechanistic Insights into Organic Abatement

Cyrille Ghislain Fotsop[a], Alexandra Lieb[a], Franziska Scheffler[a]

[a] Otto-von-Guericke-University Magdeburg, Chemical Institute, Chair for Industrial Chemistry, Universitätsplatz 2, 39106 Magdeburg, Germany

2.2.8. Characterization Method

The crystalline phases of the synthesized materials were identified by X-ray diffraction (XRD) recorded on a PANalytical MPD X'Pert Pro diffractometer (PANalytical's Empyrean, Almelo, The Netherlands) operating with Cu Kα radiation (λ = 1.544,426 Å). Fourier transform infrared (FTIR) measurements were performed on a FTIR spectrometer (Thermo Scientific Nicolet iS50, USA) in the 4000-200 cm^{-1} range. Crystal growth of the samples and surface morphology were characterized using a FE-SEM (XL30 ESEM from FEI, Hillsboro, equipped with a Genesis energy dispersive X-ray detector from EDAX, Mahwah, USA). Thermogravimetry and differential scanning calorimetry were carried out using a STA 449C Jupiter de Netzsch, Selb, Germany, (heating rate 5°C/min under N_2,). The textural characteristics were determined from the N_2 sorption using a 3Flex Share instrument (Micromeritics GmbH, Norcross, USA). UV-Vis DRS was performed using a Perkin Elmer Lambda 650S UV/Vis spectrometer. The photoluminescence (PL) emission spectra of the powder samples were recorded from 200 to 850 nm at room temperature using an Agilent Technologies Cary Eclipse Fluorescence Spectrophotometer (Cary Eclipse G9800A) with an excitation wavelength of 315 nm. X-ray photoelectron spectroscopy (XPS) analysis was performed on a Kratos Axis Ultra (DLD, USA) instrument. Raman analysis was performed using a LabRAM HR Evolution (Horiba, Oberursel, Germany) with a monochromatic laser at a wavelength of 532 nm. Zero-charge-point pH (pHZPC) was determined by measuring zeta potential. Experiments were conducted in the pH range of 2 to 10 using a Malvern Instruments Limited analyzer (Malvern, Worcestershire, WR14 1XZ, UK).

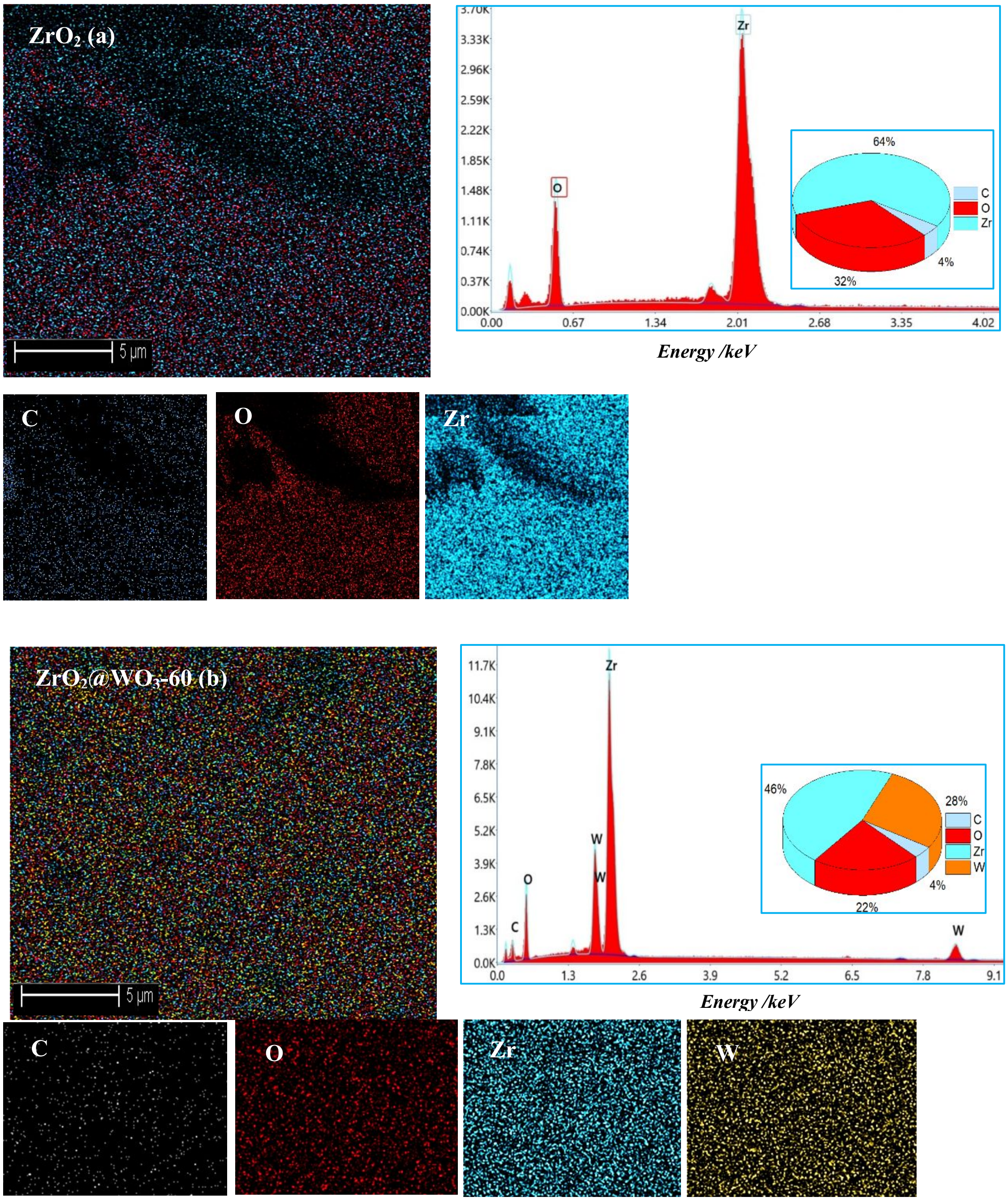

Fig. S1: EDX/mapping and EDX of ZrO_2 (a) and $ZrO_2@WO_3$-60 (b)

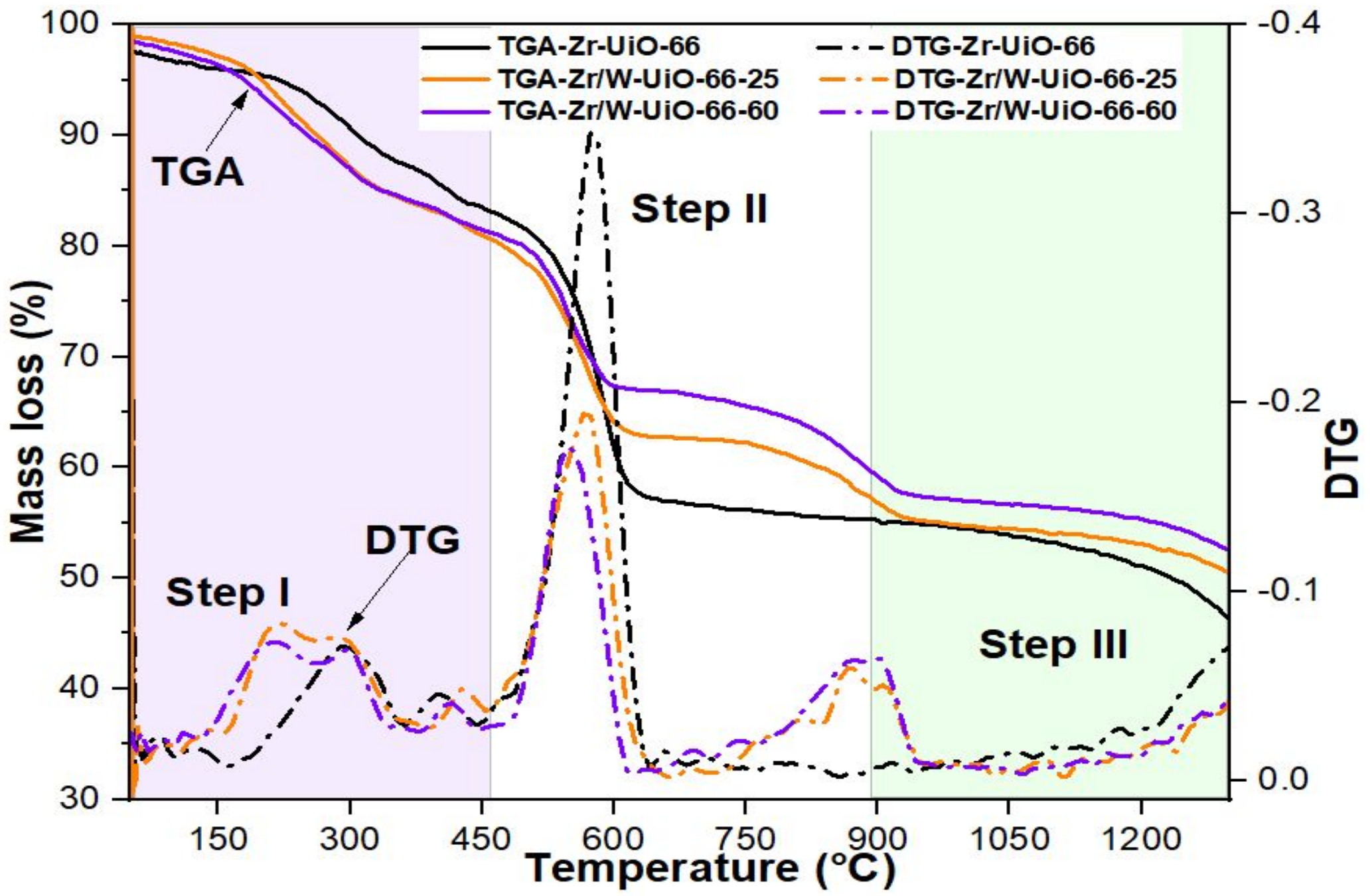

Fig. S2. TGA/DTG curves of UiO-66 and Zr/W-UiO-66

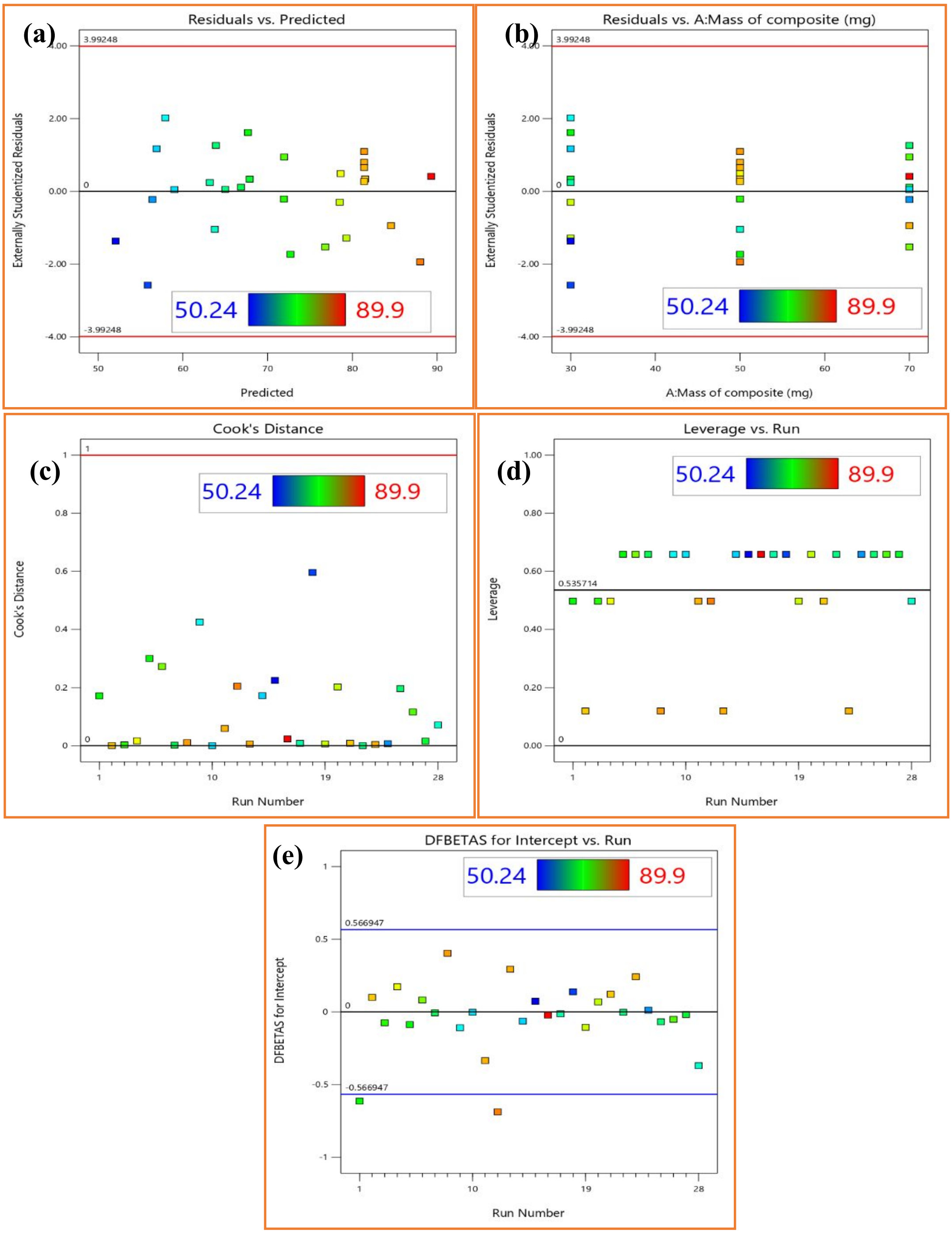

Fig. S3. The effect of factor of residual vs. predicted (a), residual vs. mass of composite (b), Cook's distance (c), Leverage vs. run (d), and DFBETAS for intercept vs. run (e) for the degradation of IC

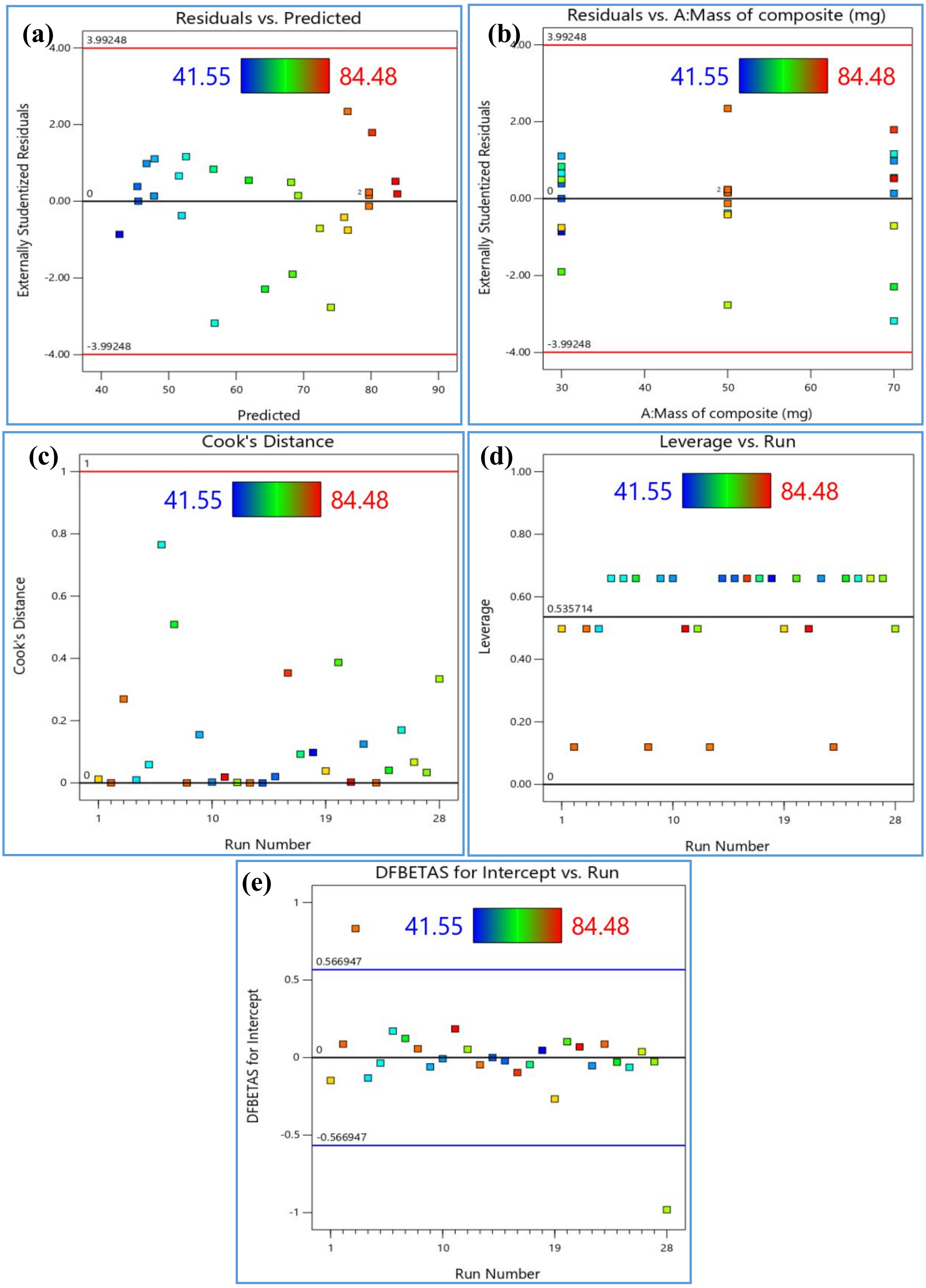

Fig. S4. The effect of factor of residual vs. predicted (a), residual vs. mass of composite (b), Cook's distance (c), Leverage vs. run (d), and DFBETAS for intercept vs. run (e) for the degradation of MB

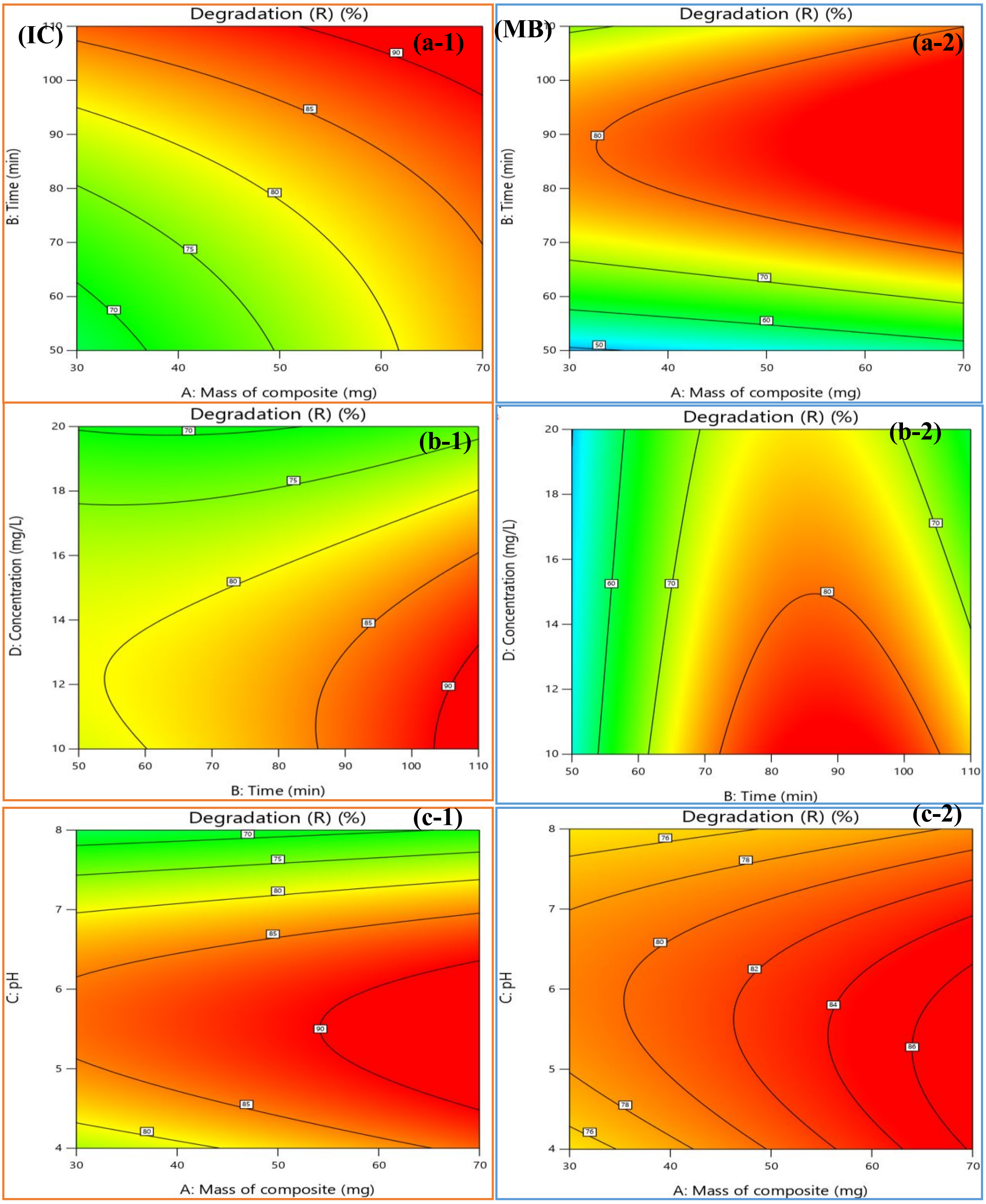

Fig. S5. 2D surface plot showing the degradation rate (%) against the effects of the mass of the composite versus time (a-1, a-2), time versus concentration (b-1, b-2) and the mass of the composite versus pH (c-1, c-2) for IC and MB, respectively.

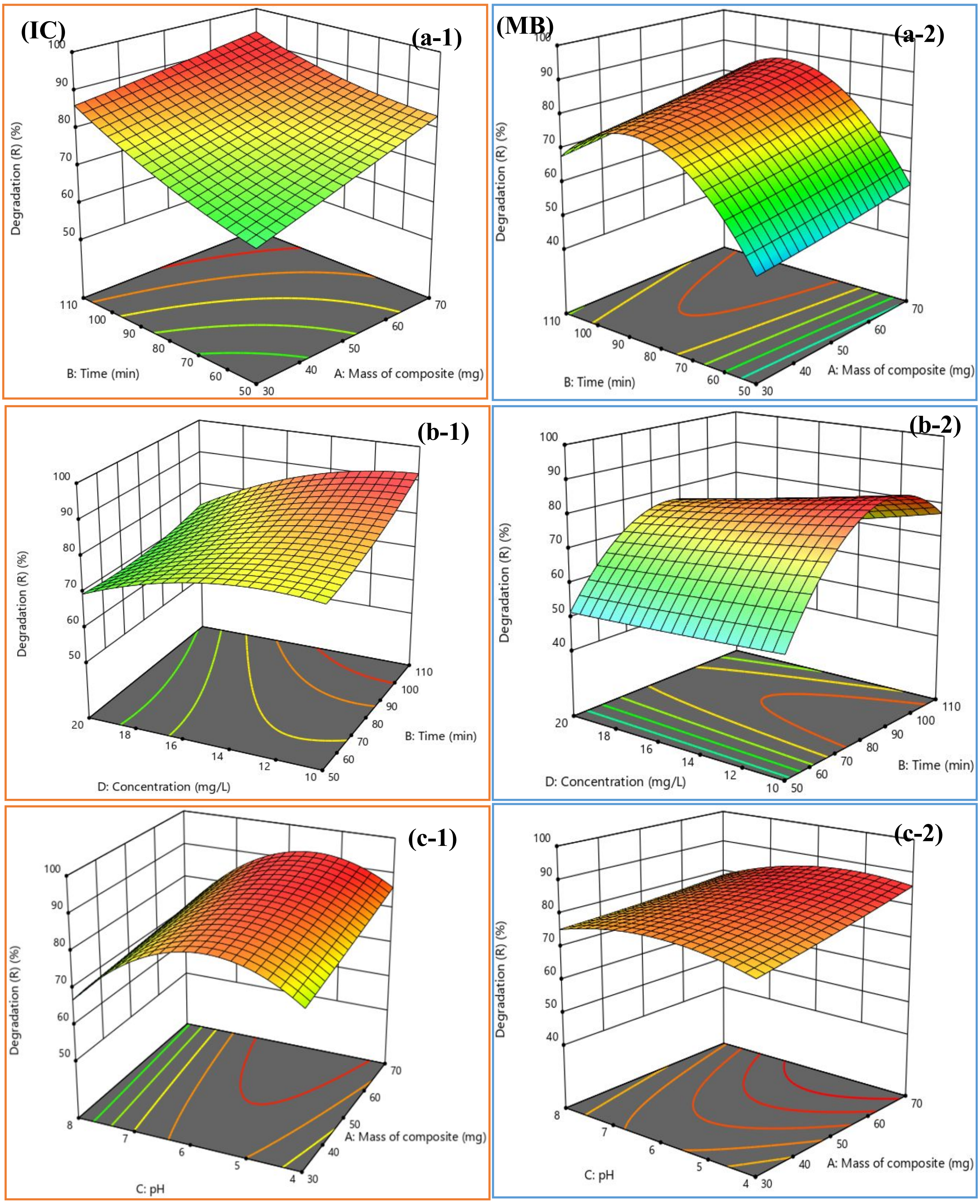

Fig. S6. 3D surface plot showing the degradation rate (%) against the effects of the mass of the composite versus time (a-1, a-2), time versus concentration (b-1, b-2) and the mass of the composite versus pH (c-1, c-2) for IC and MB, respectively.

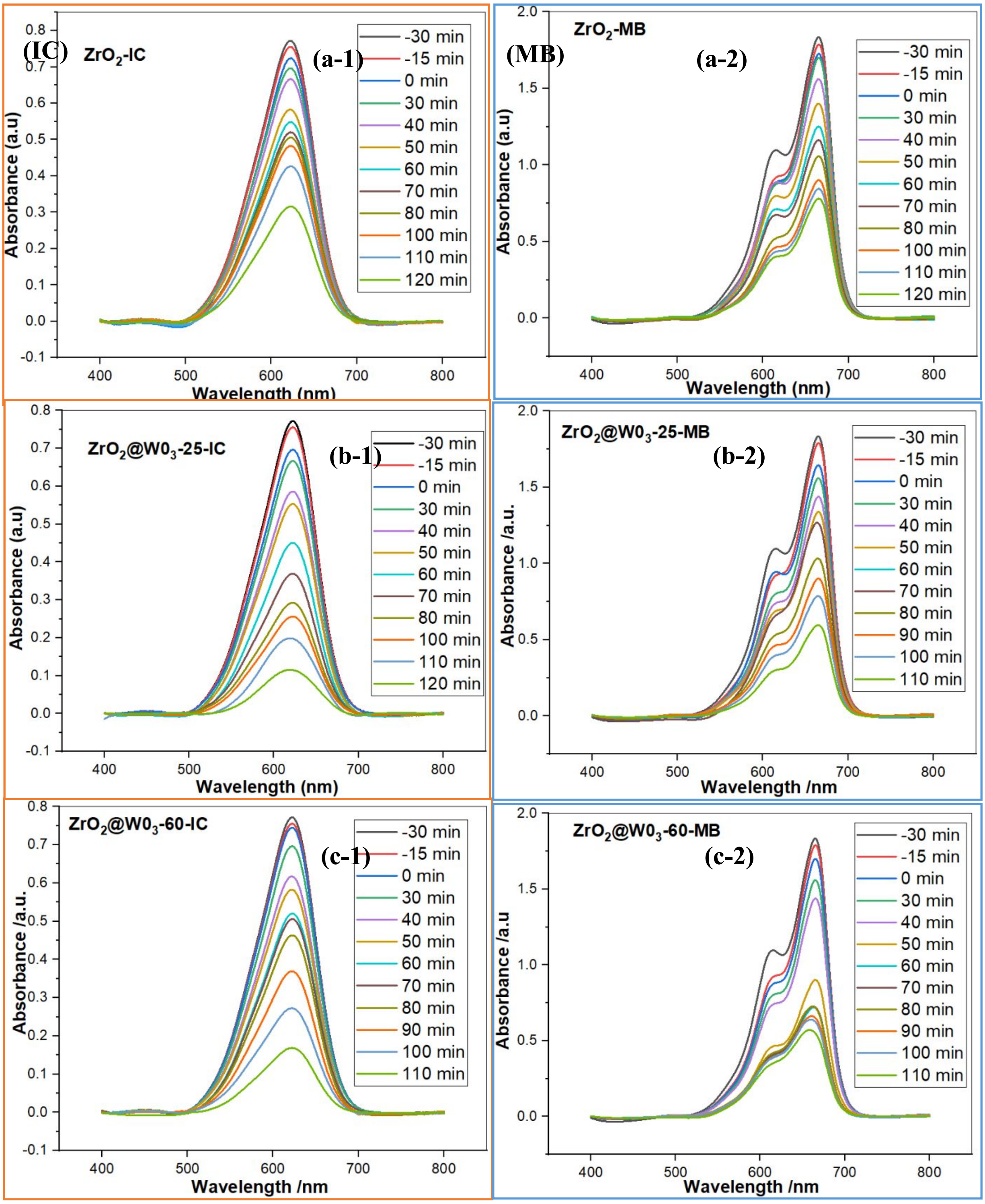
(IC)
ZrO2-IC
(a-1)
-30 min
-15 min
0 min
30 min
40 min
50 min
60 min
70 min
80 min
100 min
110 min
120 min
Absorbance (a.u)
Wavelength (nm)
(MB)
ZrO2-MB
(a-2)
-30 min
-15 min
0 min
30 min
40 min
50 min
60 min
70 min
80 min
100 min
110 min
120 min
Absorbance (a.u)
Wavelength (nm)
ZrO2@W03-25-IC
(b-1)
-30 min
-15 min
0 min
30 min
40 min
50 min
60 min
70 min
80 min
100 min
110 min
120 min
Absorbance (a.u)
Wavelength (nm)
ZrO2@W03-25-MB
(b-2)
-30 min
-15 min
0 min
30 min
40 min
50 min
60 min
70 min
80 min
90 min
100 min
110 min
Absorbance /a.u.
Wavelength /nm
ZrO2@W03-60-IC
(c-1)
-30 min
-15 min
0 min
30 min
40 min
50 min
60 min
70 min
80 min
90 min
100 min
110 min
Absorbance /a.u.
Wavelength /nm
ZrO2@W03-60-MB
(c-2)
-30 min
-15 min
0 min
30 min
40 min
50 min
60 min
70 min
80 min
90 min
100 min
110 min
Absorbance (a.u)
Wavelength /nm

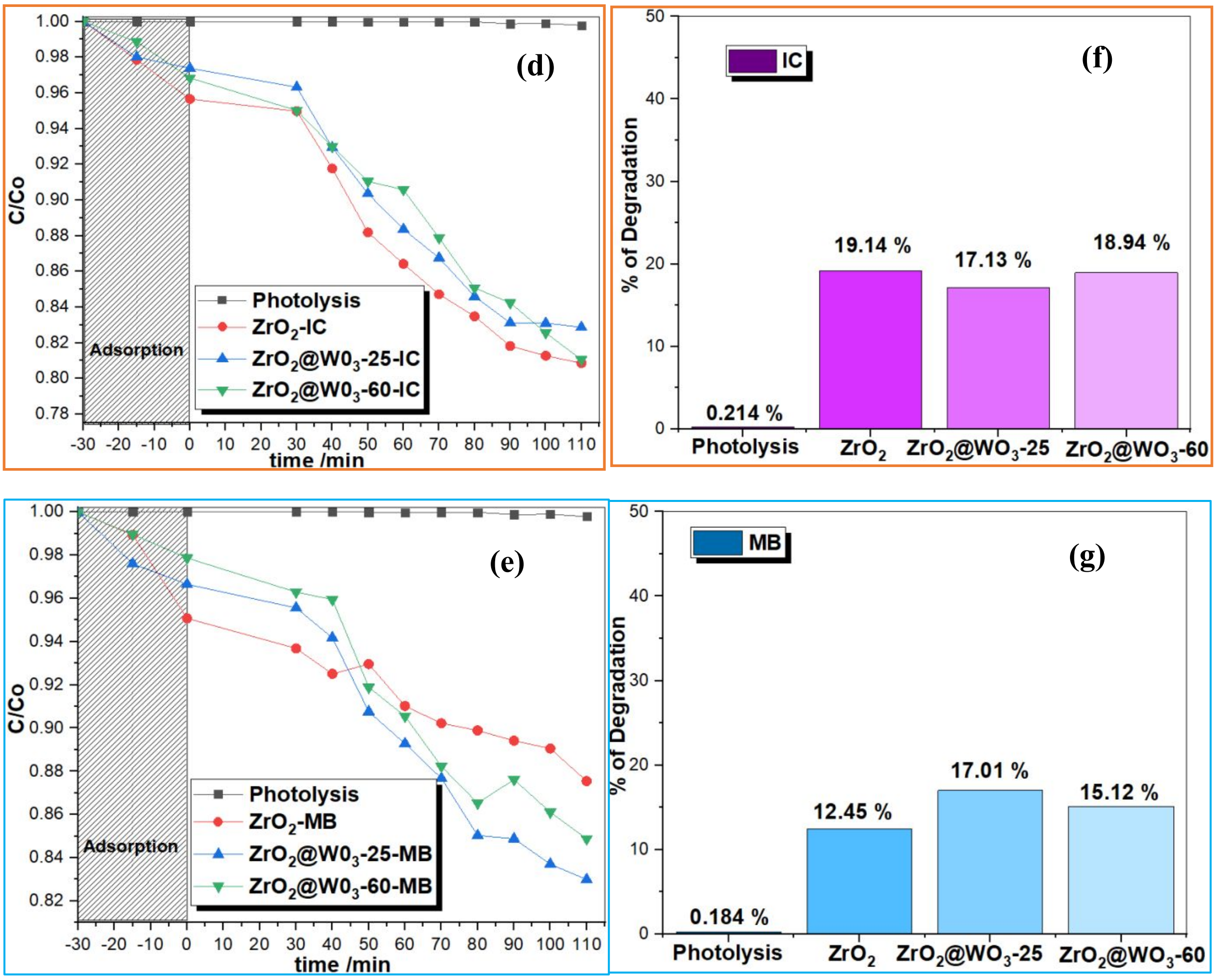

Fig. S7. UV-visible spectra before optimization: IC (a-1, b-1, c-1) and MB (a-2, b-2, c-2). Catalytic activity without light irradiation for IC (d) and MB (e); degradation rates for IC (f) and MB (g) on ZrO_2, ZrO_2@WO_3-25 and ZrO_2@WO_3-60.

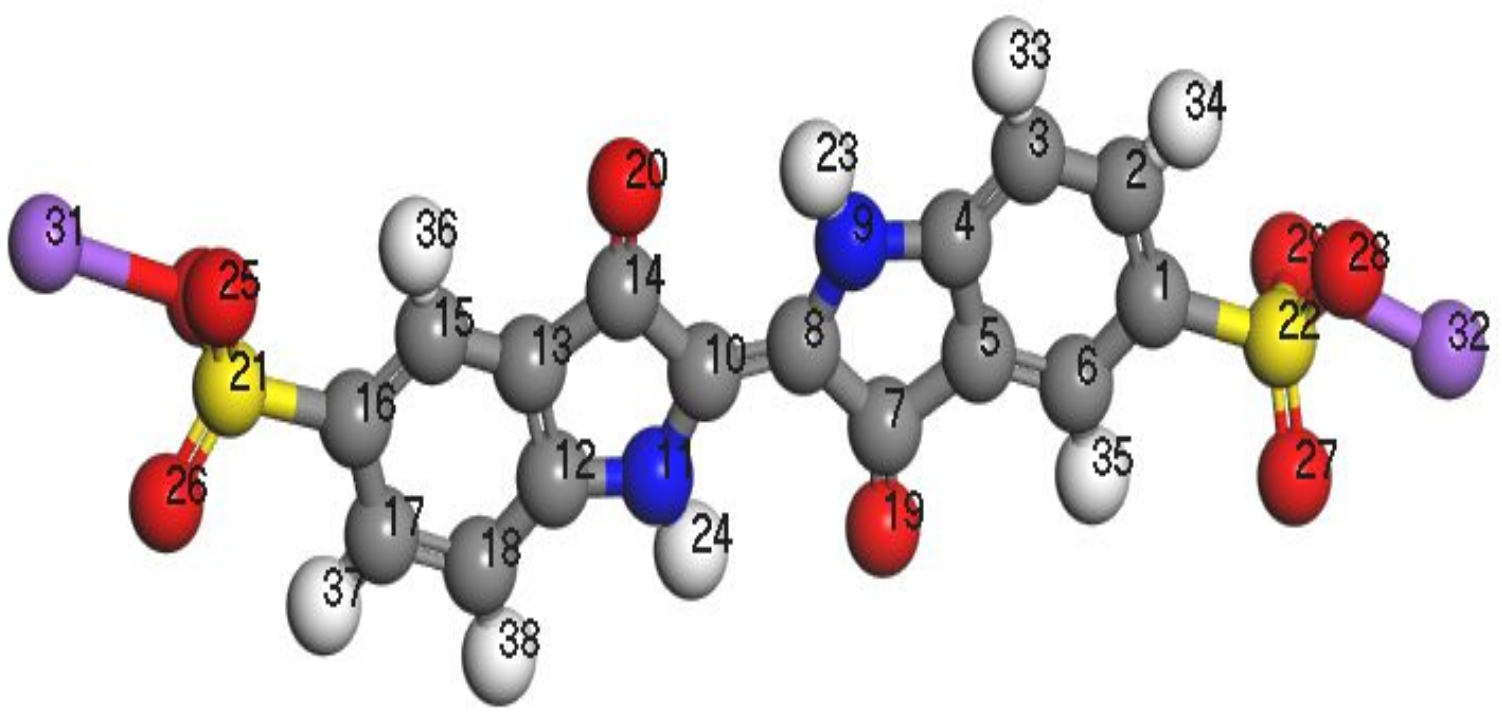

Fig. S8. IC structure

Tab. S1. Fukui Indices for

Radical Attack (Fukui(0))	Nucleophilic Attack (Fukui(+))	Electrophilic Attack (Fukui(-))

atom	Mulliken	Hirshfeld	atom	Mulliken	Hirshfeld	atom	Mulliken	Hirshfeld
C (1)	0.012	0.016	C (1)	0.012	0.016	C (1)	0.012	0.016
C (2)	0.022	0.024	C (2)	0.022	0.024	C (2)	0.022	0.024
C (3)	0.025	0.026	C (3)	0.025	0.026	C (3)	0.025	0.026
C (4)	0.018	0.016	C (4)	0.018	0.016	C (4)	0.018	0.016
C (5)	0.013	0.015	C (5)	0.013	0.015	C (5)	0.013	0.015
C (6)	0.019	0.019	C (6)	0.019	0.019	C (6)	0.019	0.019
C (7)	0.022	0.018	C (7)	0.022	0.018	C (7)	0.022	0.018
C (8)	0.016	0.015	C (8)	0.016	0.015	C (8)	0.016	0.015
N (9)	0.009	0.017	N (9)	0.009	0.017	N (9)	0.009	0.017
C (10)	0.016	0.015	C (10)	0.016	0.015	C (10)	0.016	0.015
N (11)	0.009	0.017	N (11)	0.009	0.017	N (11)	0.009	0.017
C (12)	0.018	0.016	C (12)	0.018	0.016	C (12)	0.018	0.016
C (13)	0.013	0.015	C (13)	0.013	0.015	C (13)	0.013	0.015
C (14)	0.022	0.018	C (14)	0.022	0.018	C (14)	0.022	0.018
C (15)	0.019	0.019	C (15)	0.019	0.019	C (15)	0.019	0.019
C (16)	0.012	0.016	C (16)	0.012	0.016	C (16)	0.012	0.016
C (17)	0.022	0.024	C (17)	0.022	0.024	C (17)	0.022	0.024
C (18)	0.025	0.026	C (18)	0.025	0.026	C (18)	0.025	0.026
O (19)	0.031	0.034	O (19)	0.031	0.034	O (19)	0.031	0.034
O (20)	0.031	0.034	O (20)	0.031	0.034	O (20)	0.031	0.034
S (21)	0.025	0.029	S (21)	0.025	0.029	S (21)	0.025	0.029
S (22)	0.025	0.029	S (22)	0.025	0.029	S (22)	0.025	0.029
H (23)	0.016	0.013	H (23)	0.016	0.013	H (23)	0.016	0.013
H (24)	0.016	0.013	H (24)	0.016	0.013	H (24)	0.016	0.013
O (25)	0.035	0.036	O (25)	0.035	0.036	O (25)	0.035	0.036
O (26)	0.039	0.039	O (26)	0.039	0.039	O (26)	0.039	0.039
O (27)	0.035	0.036	O (27)	0.035	0.036	O (27)	0.035	0.036
O (28)	0.039	0.039	O (28)	0.039	0.039	O (28)	0.039	0.039
O (29)	0.031	0.032	O (29)	0.031	0.032	O (29)	0.031	0.032
O (30)	0.031	0.032	O (30)	0.031	0.032	O (30)	0.031	0.032
Na(31)	0.110	0.104	Na(31)	0.110	0.104	Na(31)	0.110	0.104
Na(32)	0.110	0.104	Na(32)	0.110	0.104	Na(32)	0.110	0.104
H (33)	0.021	0.018	H (33)	0.021	0.018	H (33)	0.021	0.018
H (34)	0.020	0.017	H (34)	0.020	0.017	H (34)	0.020	0.017
H (35)	0.016	0.013	H (35)	0.016	0.013	H (35)	0.016	0.013
H (36)	0.016	0.013	H (36)	0.016	0.013	H (36)	0.016	0.013
H (37)	0.020	0.017	H (37)	0.020	0.017	H (37)	0.020	0.017
H (38)	0.021	0.018	H (38)	0.021	0.018	H (38)	0.021	0.018

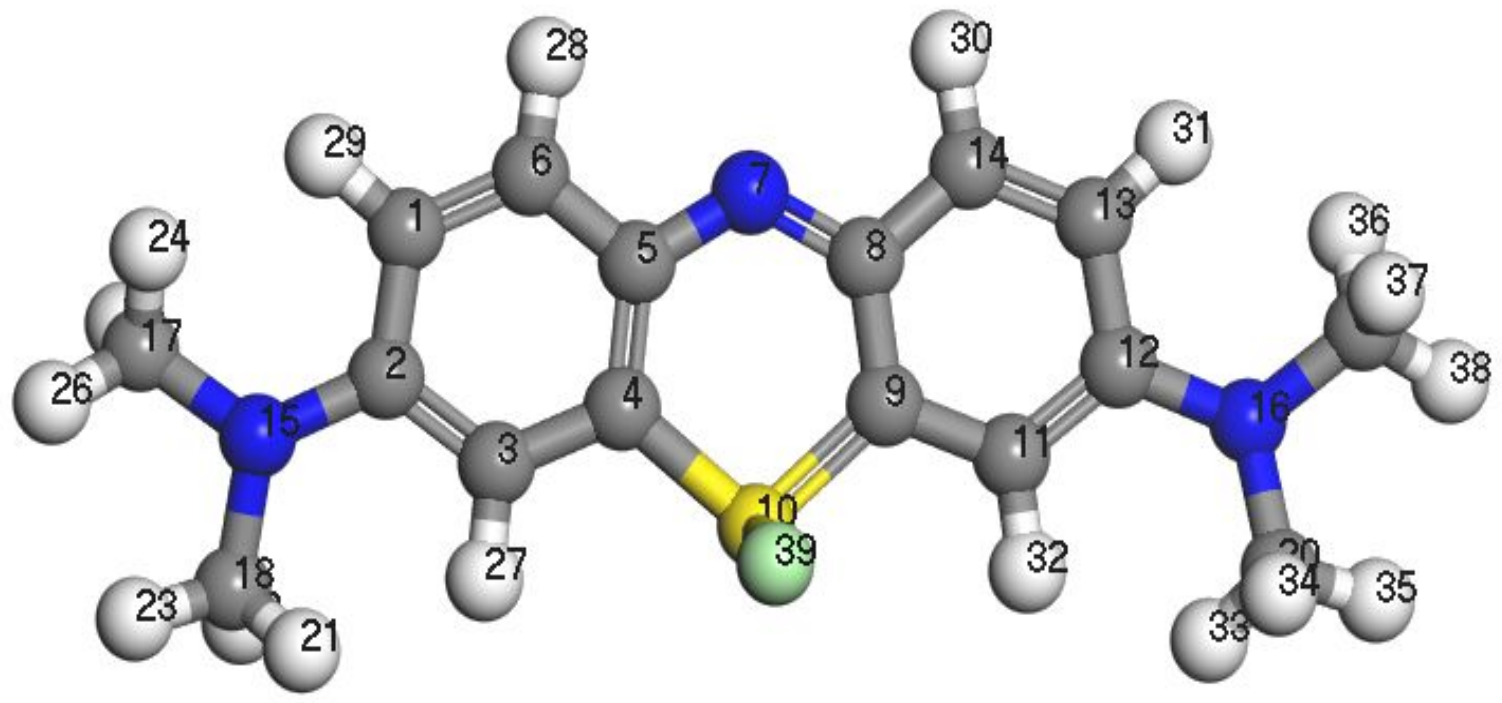

Fig. S9. MB structure

Tab. S2. Fukui Indices for

Radical Attack (Fukui(0))			Nucleophilic Attack (Fukui(+))			Electrophilic Attack (Fukui(-))		
**atom**	**Mulliken**	**Hirshfeld**	**atom**	**Mulliken**	**Hirshfeld**	**atom**	**Mulliken**	**Hirshfeld**
C (1)	0.026	0.028	C (1)	0.026	0.028	C (1)	0.026	0.028
C (2)	0.020	0.019	C (2)	0.020	0.019	C (2)	0.020	0.019
C (3)	0.021	0.022	C (3)	0.021	0.022	C (3)	0.021	0.022
C (4)	0.013	0.016	C (4)	0.013	0.016	C (4)	0.013	0.016
C (5)	0.018	0.018	C (5)	0.018	0.018	C (5)	0.018	0.018
C (6)	0.030	0.030	C (6)	0.030	0.030	C (6)	0.030	0.030
N (7)	0.022	0.026	N (7)	0.022	0.026	N (7)	0.022	0.026
C (8)	0.018	0.018	C (8)	0.018	0.018	C (8)	0.018	0.018
C (9)	0.013	0.016	C (9)	0.013	0.016	C (9)	0.013	0.016
S (10)	0.037	0.039	S (10)	0.037	0.039	S (10)	0.037	0.039
C (11)	0.021	0.022	C (11)	0.021	0.022	C (11)	0.021	0.022
C (12)	0.020	0.019	C (12)	0.020	0.019	C (12)	0.020	0.019
C (13)	0.026	0.028	C (13)	0.026	0.028	C (13)	0.026	0.028
C (14)	0.030	0.030	C (14)	0.030	0.030	C (14)	0.030	0.030
N (15)	0.010	0.021	N (15)	0.010	0.021	N (15)	0.010	0.021
N (16)	0.010	0.021	N (16)	0.010	0.021	N (16)	0.010	0.021
C (17)	0.040	0.041	C (17)	0.040	0.041	C (17)	0.040	0.041
C (18)	0.038	0.039	C (18)	0.038	0.039	C (18)	0.038	0.039
C (19)	0.040	0.041	C (19)	0.040	0.041	C (19)	0.040	0.041
C (20)	0.038	0.039	C (20)	0.038	0.039	C (20)	0.038	0.039
H (21)	0.022	0.021	H (21)	0.022	0.021	H (21)	0.022	0.021
H (22)	0.025	0.023	H (22)	0.025	0.023	H (22)	0.025	0.023
H (23)	0.028	0.025	H (23)	0.028	0.025	H (23)	0.028	0.025
H (24)	0.024	0.023	H (24)	0.024	0.023	H (24)	0.024	0.023
H (25)	0.026	0.024	H (25)	0.026	0.024	H (25)	0.026	0.024
H (26)	0.028	0.026	H (26)	0.028	0.026	H (26)	0.028	0.026
H (27)	0.018	0.015	H (27)	0.018	0.015	H (27)	0.018	0.015
H (28)	0.026	0.023	H (28)	0.026	0.023	H (28)	0.026	0.023
H (29)	0.023	0.020	H (29)	0.023	0.020	H (29)	0.023	0.020
H (30)	0.026	0.023	H (30)	0.026	0.023	H (30)	0.026	0.023
H (31)	0.023	0.020	H (31)	0.023	0.020	H (31)	0.023	0.020

H (32)	0.018	0.015	H (32)	0.018	0.015	H (32)	0.018	0.015
H (33)	0.022	0.021	H (33)	0.022	0.021	H (33)	0.022	0.021
H (34)	0.024	0.023	H (34)	0.024	0.023	H (34)	0.024	0.023
H (35)	0.028	0.025	H (35)	0.028	0.025	H (35)	0.028	0.025
H (36)	0.024	0.023	H (36)	0.024	0.023	H (36)	0.024	0.023
H (37)	0.026	0.024	H (37)	0.026	0.024	H (37)	0.026	0.024
H (38)	0.028	0.026	H (38)	0.028	0.026	H (38)	0.028	0.026
Cl(39)	0.070	0.069	Cl(39)	0.070	0.069	Cl(39)	0.070	0.069

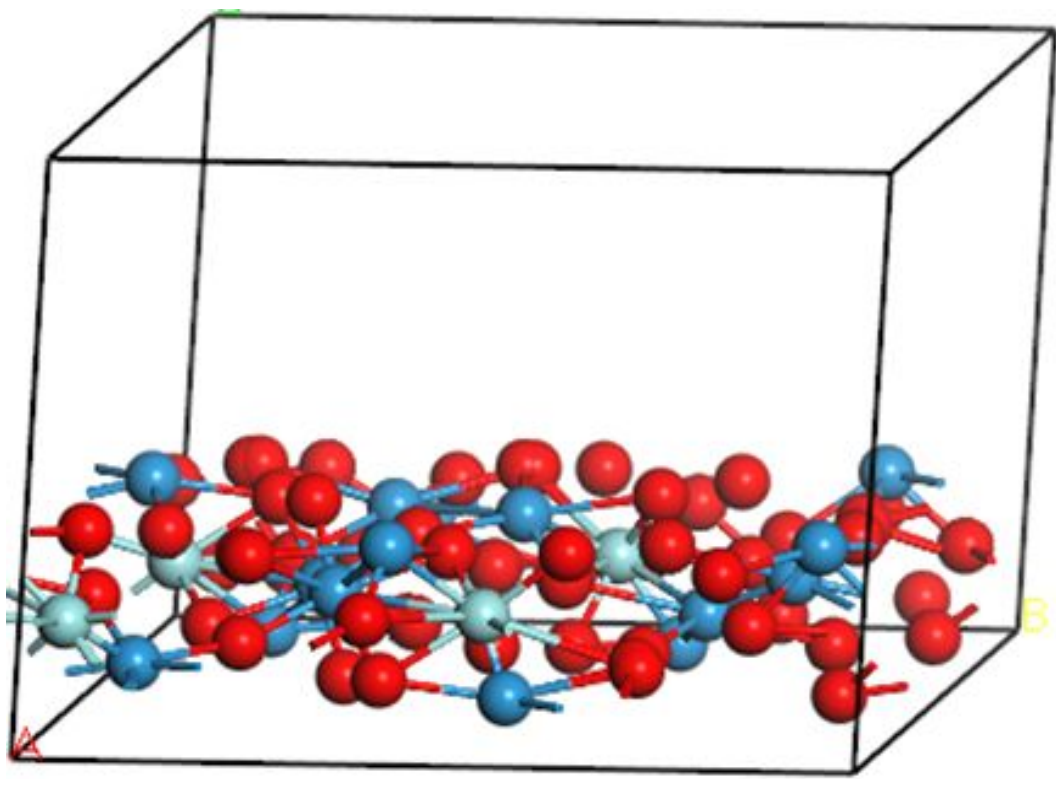

Fig. S10. Reduced 3D structure of ZrO_2@WO_3

(a)

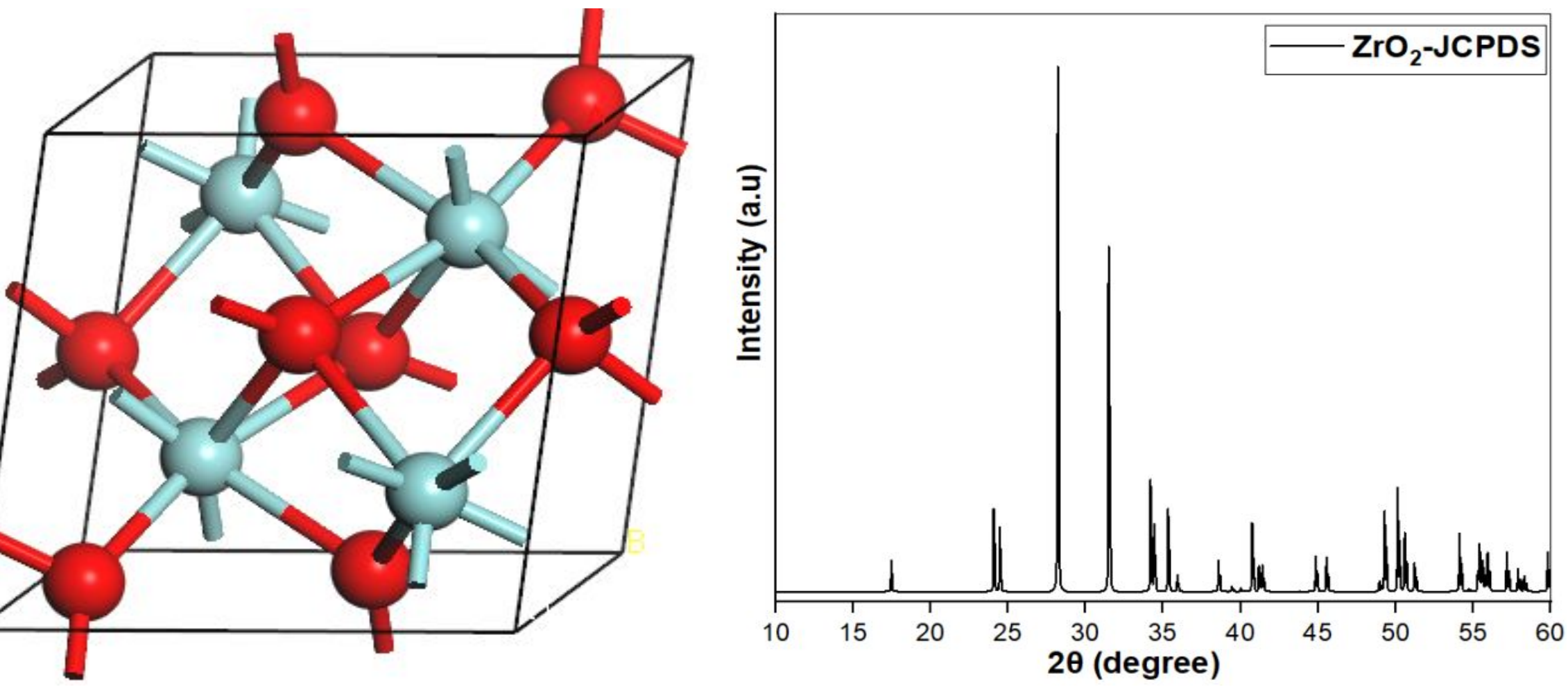

(b)

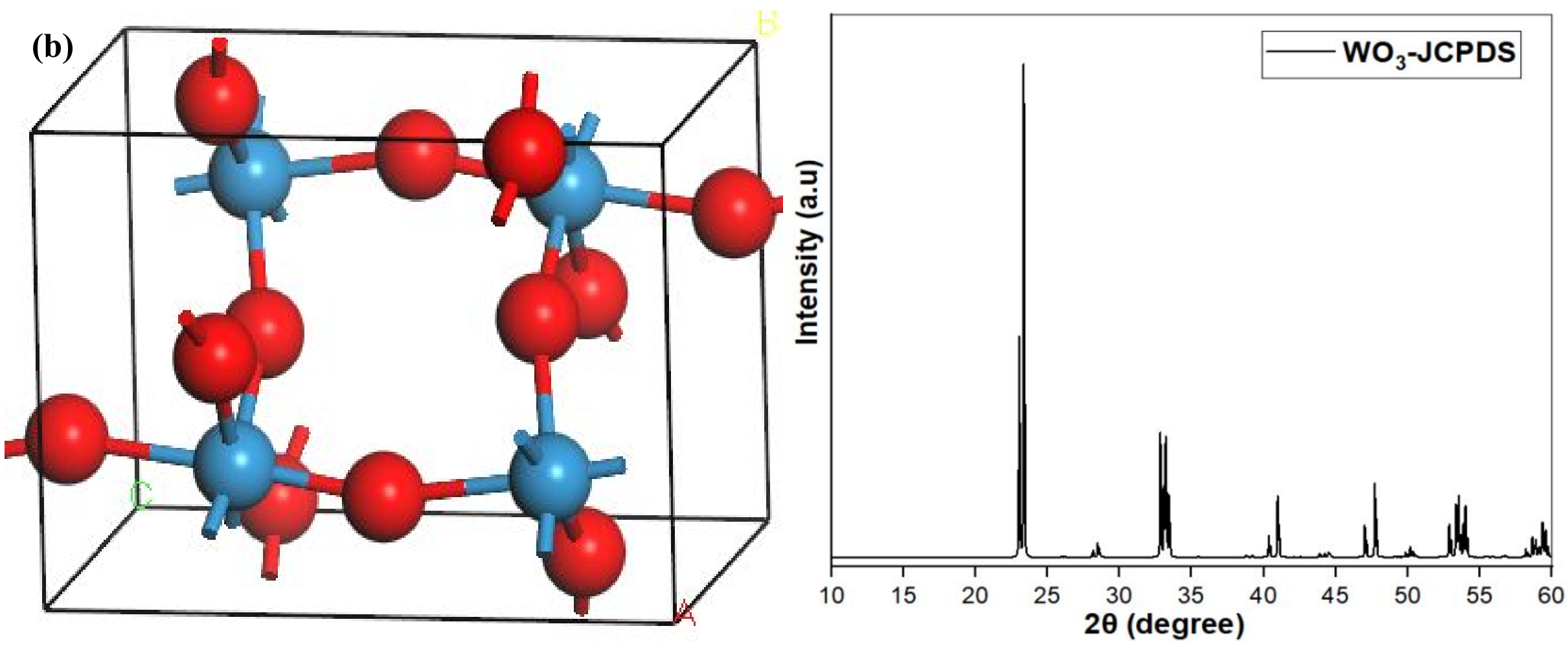

Fig. S11. 3D structure and XRD pattern of ZrO_2 (a) and WO_3 (b) used in the DFT calculations

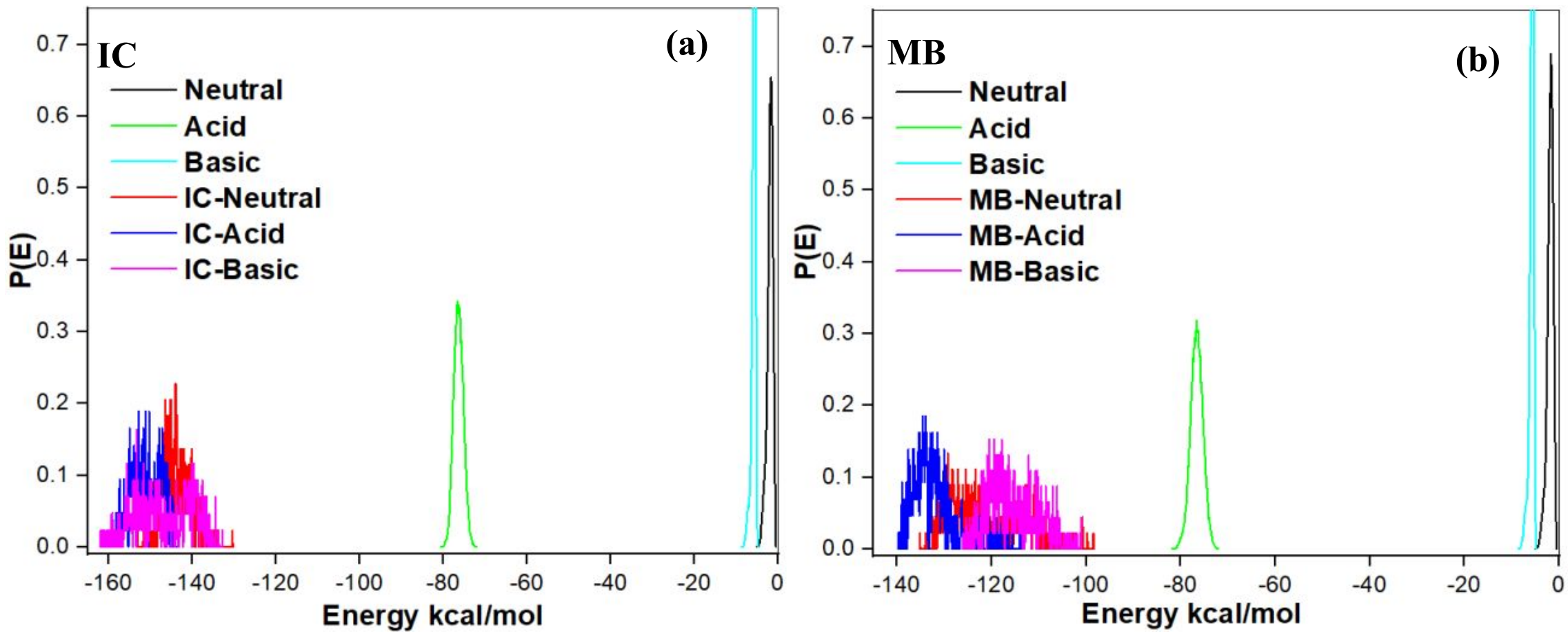

Fig. S12. Probability of energy distribution for IC (a) and MB (b) in different medium

Tab. S3. Dynamic adsorption descriptors for IC and MB on $ZrO_2@WO_3$ in different medium (kcal/mol)

			**IC**			
	**Total energy**	**Adsorption energy**	**Rigid adsorption energy**	**Deformation energy**	**medium: dEad/dNi**	**IC : dEad/dNi**
**Neutral**	508.14	-901.77	-346.14	-555.63	-0.606	-142.03
**Acidic**	-380.51	-30032.94	-1089.08	-28943.86	-73.03	-146.71
**Basic**	584.42	-2543.95	-270.69	-2273.26	-4.96	-142.18
			**MB**			
	**Total energy**	**Adsorption energy**	**Rigid adsorption energy**	**Deformation energy**	**medium: dEad/dNi**	**MB : dEad/dNi**
**Neutral**	-123.84	-818.11	-352.92	-465.17	-0.613	-115.66
**Acidic**	-1.09E+03	-3.00E+04	-1.17E+03	-2.89E+04	-7.28E+01	-1.31E+02
**Basic**	-34.96	-2447.69	-262.15	-2185.53	-4.93	-112.39

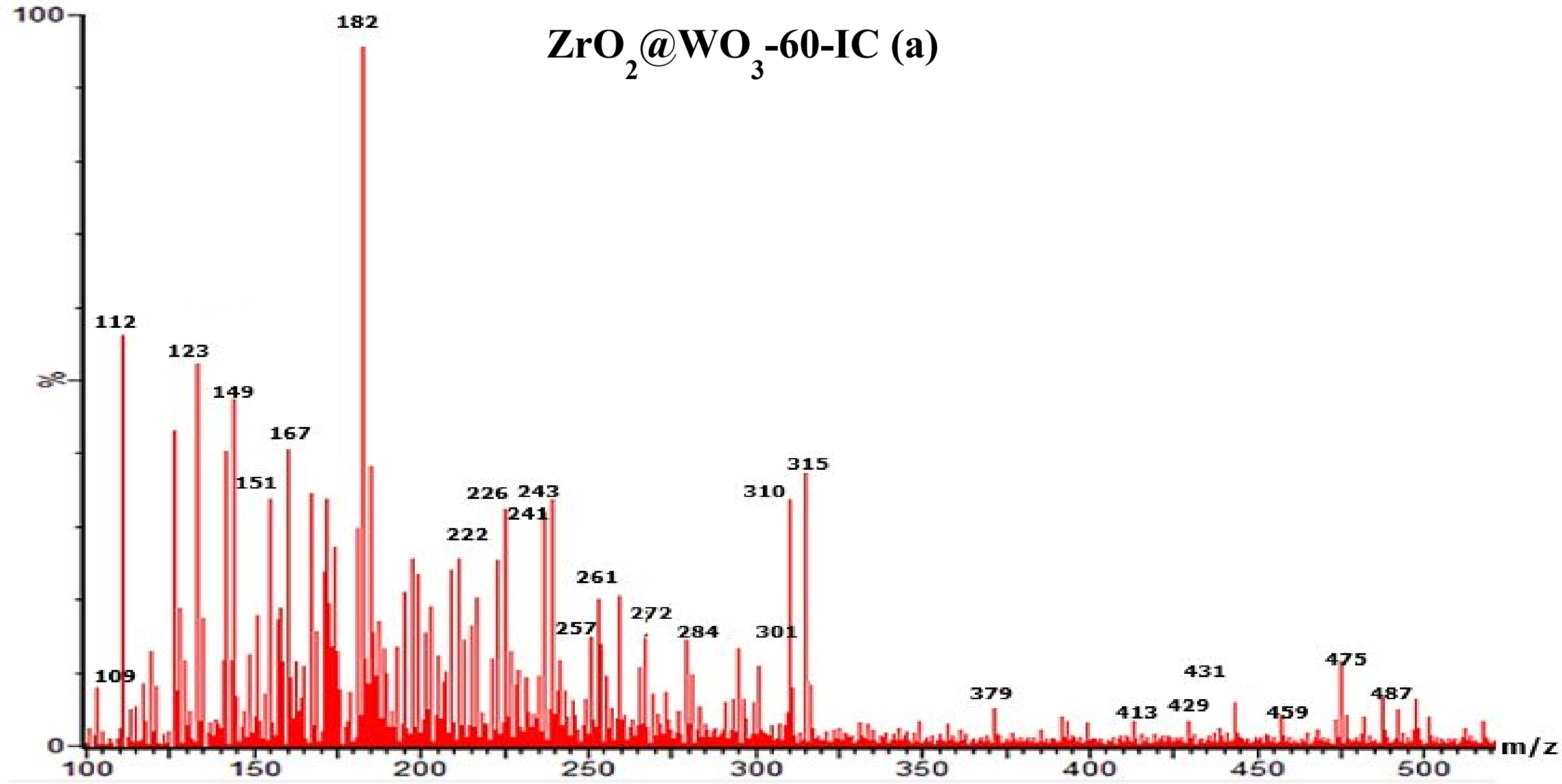

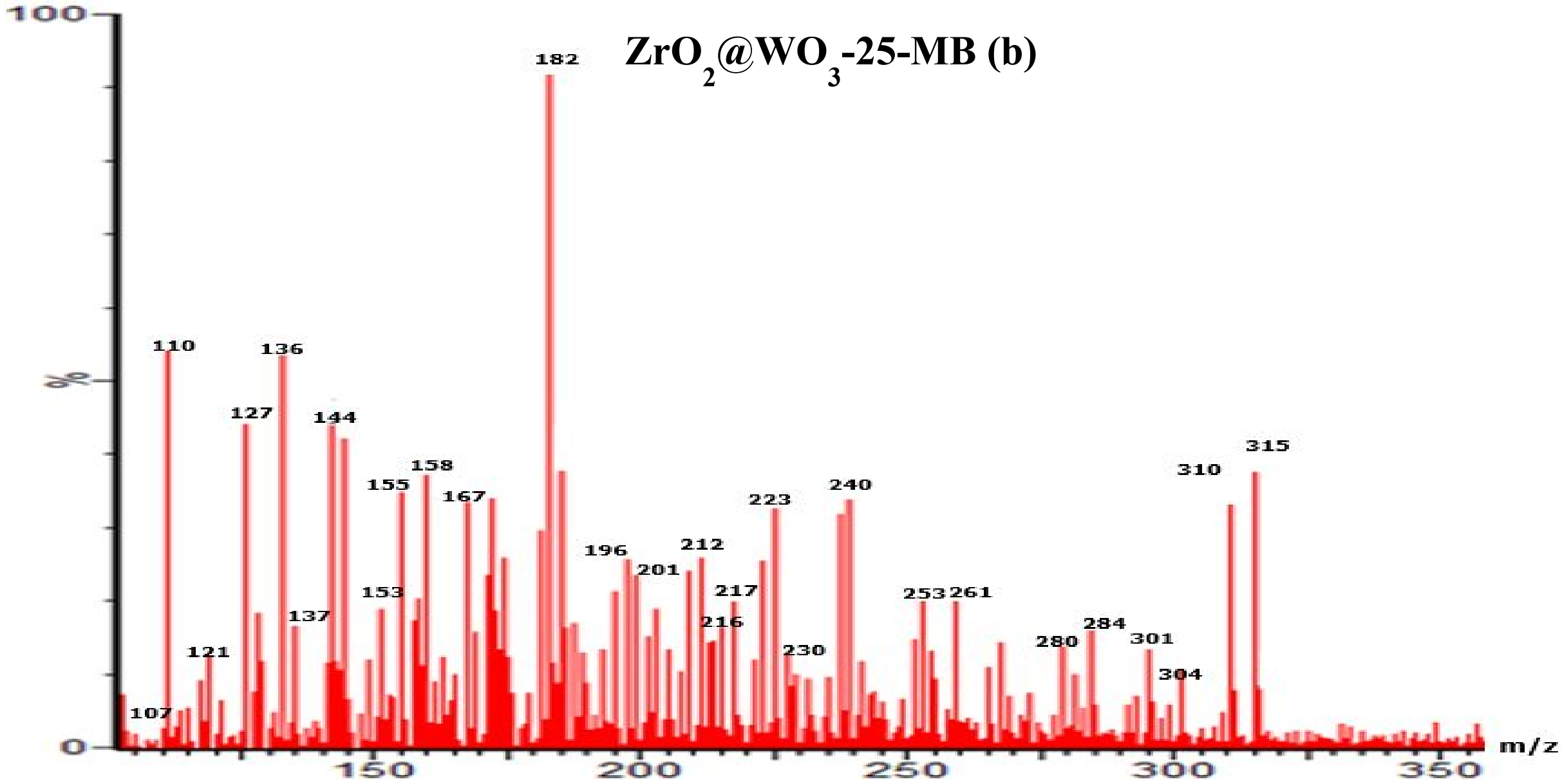

Fig. S13. LC-MS spectra of IC (a) and MB (b)

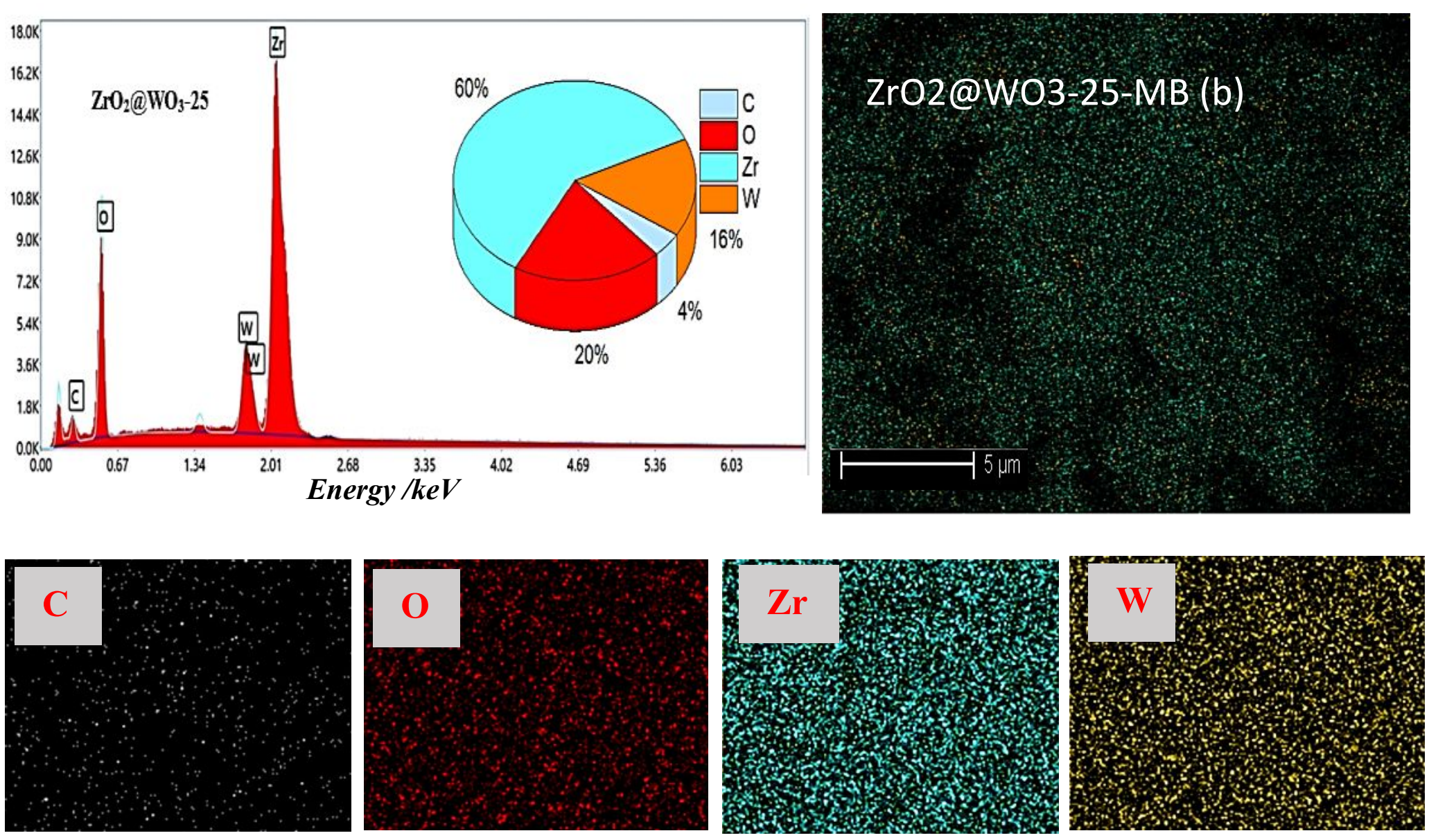

Fig. S14. EDX-EDX/mapping of ZrO_2@WO_3-25 after MB degradation

Tab. S4. Summary of degradation efficiency of some catalysts

Samples	Synthesis approach	Shape	Dye	Light source	% Degradation efficiency (time /min)	References
TiO_2-ZnO/rGO	hydrothermal	Rod/flower	Indigo carmine	UV	~ 95 (80)	[1]
TiO_2	Sol - gel	Donut-like	Methylene blue	UV	67 (200)	[2]
MgO@g-C_3N_4	Ultrasonic	Spherical	Indigo carmine	UV	97 (60)<br>28 (60)<br>10 (60)	[3]

TiO_2	Solvent-free Precipitation	Spherical	Methylene blue	Sunlight	>95 (120)	[4]
NiO	Abelmoschus esculentus seed extract	Spherical	Methylene blue	UV	80(240)	[5]
ZnO	P. austroarabica leaf extract	Spherical	Congo red<br>Methylene blue	UV	93(10)<br>95(15)	[6]
SnO_2	Waste cotton peel extract	Spherical	Methylene blue<br>Methyl orange	UV	30 – 80<br>96(40 – 100)	[7]
PbO	ageretia thea leaf extract	Quasi-spherica	Crystal violet	Sunlight	69(480)	[8]
ZrO_2@WO_3-25	hydrothermal	Hexagonal/ Spherical	Methylene blue	UV	95(110)	**this work**
ZrO_2@WO_3-60	hydrothermal	Hexagonal/ Spherical	Indigo carmine	UV	96(110)	**this work**

References

(1) Fadillah, G.; Hidayat, R.; Saleh, T. A. Hydrothermal Assisted Synthesis of Titanium Dioxide Nanoparticles Modified Graphene with Enhanced Photocatalytic Performance. *J. Ind. Eng. Chem.* **2022**, *113*, 411–418. https://doi.org/10.1016/j.jiec.2022.06.016.

(2) Kudhier, M. A.; Sabry, R. S.; Al-Haidarie, Y. K. Novel and Simple Method to Synthesize Donut-like TiO 2 with Photocatalytic Activity. *Mater. Sci. Semicond. Process.* **2018**, *73*, 35–41. https://doi.org/10.1016/j.mssp.2017.05.034.

(3) Toghan, A.; Modwi, A. Boosting Unprecedented Indigo Carmine Dye Photodegradation via Mesoporous MgO@g-C3N4 Nanocomposite. *J. Photochem. Photobiol. Chem.* **2021**, *419*, 113467. https://doi.org/10.1016/j.jphotochem.2021.113467.

(4) S. Muniandy, S.; Mohd Kaus, N. H.; Jiang, Z.-T.; Altarawneh, M.; Lee, H. L. Green Synthesis of Mesoporous Anatase TiO_2 Nanoparticles and Their Photocatalytic Activities. *RSC Adv* **2017**, *7* (76), 48083–48094. https://doi.org/10.1039/C7RA08187A.

(5) Aslam, M.; Abdullah, A. Z.; Rafatullah, M.; Fawad, A. Abelmoschus Esculentus (Okra) Seed Extract for Stabilization of the Biosynthesized TiO2 Photocatalyst Used for Degradation of Stable Organic Substance in Water. *Environ. Sci. Pollut. Res.* **2022**, *29* (27), 41053–41064. https://doi.org/10.1007/s11356-021-18066-1.

(6) Alahdal, F. A. M.; Qashqoosh, M. T. A.; Manea, Y. K.; Salem, M. A. S.; Khan, A. M. T.; Naqvi, S. Eco-Friendly Synthesis of Zinc Oxide Nanoparticles as Nanosensor, Nanocatalyst and Antioxidant Agent Using Leaf Extract of P. Austroarabica. *OpenNano* **2022**, *8*, 100067. https://doi.org/10.1016/j.onano.2022.100067.

(7) Narasaiah, B. P.; Banoth, P.; Sohan, A.; Mandal, B. K.; Bustamante Dominguez, A. G.; De Los Santos Valladares, L.; Kollu, P. Green Biosynthesis of Tin Oxide Nanomaterials Mediated by Agro-Waste Cotton Boll Peel Extracts for the Remediation of Environmental Pollutant Dyes. *ACS Omega* **2022**, *7* (18), 15423–15438. https://doi.org/10.1021/acsomega.1c07099.

(8) Talha Khalil, A.; Hameed, S.; Afridi, S.; Mohamed, H. E. A.; Shinwari, Z. K. Sageretia Thea Mediated Biosynthesis of Metal Oxide Nanoparticles for Catalytic Degradation of Crystal Violet Dye. *Mater. Today Proc.* **2021**, *36*, 397–400. https://doi.org/10.1016/j.matpr.2020.04.687.
